# Loss of DET1 in *High Pigment 2* Tomato Prevents High Temperature Repression of Anthocyanin Biosynthesis in Fruit Through HY5 Stabilization

**DOI:** 10.1111/pce.15586

**Published:** 2025-05-11

**Authors:** Jacopo Menconi, Noemi La Monaca, Irene Cataldo, Pietro Maria Niccolini, Pierdomenico Perata, Silvia Gonzali

**Affiliations:** ^1^ PlantLab, Institute of Plant Sciences Scuola Superiore Sant'Anna Pisa Italy; ^2^ Department of Biology University of Pisa Pisa Italy

**Keywords:** anthocyanins, climate change, constitutive photomorphogenic 1 (COP1), deetiolated 1 (DET1), elongated hypocotyl 5 (HY5), heatwaves, high pigment 2 (hp2), nutraceutical value, photomorphogenesis, thermomorphogenesis, tomato

## Abstract

Global warming impacts several aspects of plant physiology, with important negative effects on crop yield and production of secondary metabolites, such as anthocyanins. The anthocyanin content of vegetables and fruits has attracted public interest in the last two decades due to its health benefits, leading to the development of novel anthocyanin‐enriched plant varieties, including purple tomato lines. In purple tomato fruits anthocyanin biosynthesis is largely regulated by light through HY5, whose levels are in turn controlled by COP1‐targeted destabilization, and increasing temperatures strongly impair anthocyanin accumulation in the fruit peel. Interestingly, two different COP1‐encoding genes exist in tomato and one of them is further involved in alternative splicing, giving origin to polypeptides characterized by different lengths and, possibly, functions. High temperatures trigger HY5 degradation under light through nuclear relocation and interaction with DET1 of both COP1 tomato factors. *high pigment 2* (*hp2*) tomato plants bear a nonfunctional *det1* allele and show exacerbate photomorphogenesis due to HY5 stabilization. In this paper, we show that the COP1‐DET1‐HY5 switch is crucial for the high temperature‐induced repression of anthocyanin synthesis in purple fruits. The loss of DET1 indeed impedes COP1 activity in degrading HY5, allowing sustained anthocyanin accumulation under light even under high temperatures. All COP1 tomato factors seem to require DET1 to target HY5 to proteolysis, but only *COP1‐like X1 isoform* gene is also transcriptionally regulated by HY5, whereas the expression of *COP1 homolog* is not affected by the mutation of DET1. Furthermore, whereas the expression of *COP1 homolog* is stable and independent from temperature, the canonical transcript of *COP1‐like X1 isoform*, possibly producing the polypeptide containing all the functional domains, is also enhanced by higher temperatures. The introgression of the *hp2* mutation in purple tomato lines can thus counteract the high temperature‐induced reduction in anthocyanin accumulation, representing a key strategy to preventing global warming‐related loss of quality in tomatoes.

## Summary statement

The introgression of the natural photomorphogenic mutation hp2 (det1) in anthocyanin‐enriched tomato lines prevents anthocyanin repression in fruits at high temperatures.

## 1 Introduction

Anthocyanins are water‐soluble polyphenolic compounds which in the plant kingdom confer colours ranging from red to purple and blue (Glover and Martin 2012; Gonzali and Perata 2020; Cappellini et al. 2021). In addition to their ability to attract pollinators and seed dispersers in flowers and fruits, these pigments function as photoprotective screens to absorb excess light, as shields against UV‐B radiation, and as scavengers of free radicals (Winkel‐Shirley 2002; Butelli et al. 2008; Zhang et al. 2014; Cappellini et al. 2021). Anthocyanins have recently drawn particular interest in several plant species also as nutraceutical compounds (Colanero et al. 2020). Many efforts have thus been made to enrich the anthocyanin content in commonly consumed vegetables and fruits to boost their nutraceutical power and contribute to a healthier diet for consumers.

In the anthocyanin pathway, the biosynthetic genes encode enzymes that catalyse the chemical reactions leading from phenylalanine to anthocyanins and are controlled by specialized transcription factors (TFs), which are in turn encoded by regulatory genes (Povero et al. 2011; Brugliera et al. 2013; Vu and Lee 2019). These genes belong to multigene families and produce regulatory proteins that act alone or within complexes. The most important is the MYB‐bHLH‐WDR (MBW) complex, which is composed of R2R3‐MYB, bHLH and WD40 factors and mainly activates the late biosynthetic genes (LBGs) of the pathway (Colanero et al. 2020; Sun et al. 2020a).

Tomato (*Solanum lycopersicum)* is among the most eaten vegetables worldwide and has been recently improved to synthesize and accumulate high amount of anthocyanins in its fruits (Butelli et al. 2008; Gonzali et al. 2009; Mazzucato et al. 2013). The breeding approach for the production of anthocyanin‐enriched tomato fruits has exploited novel alleles introgressed from wild tomato relatives. *Anthocyanin fruit (Aft)* (Georgiev 1972) and *atroviolacea (atv*) (Rick et al. 1968) represent the most powerful loci used thus far. The *Aft* trait, introgressed from *Solanum chilense*, allows anthocyanin production in the peel of tomatoes under high light or low temperatures, resulting in purple‐spotted fruits (Mes et al. 2008). The *Aft* allele restores a correct version of the *AN2‐like* gene, encoding the R2R3‐MYB factor which acts as the master regulator of anthocyanins in the fruit peel and is mutated and nonfunctional in *S. lycopersicum* (Colanero *et al*. 2019; Sun et al. 2020a; Yan et al. 2019). The *atv* phenotype, introduced from *Solanum cheesmaniae*, is characterized by a higher anthocyanin concentration in the vegetative tissues of the plant (Jones. 2003). The *atv* locus includes a gene encoding MYB‐ATV, which is an R3‐MYB that acts as a negative feedback regulator of anthocyanin biosynthesis (Cao et al. 2017). The *atv* allele leads to a truncated and nonfunctional version of MYB‐ATV, thus derepressing the anthocyanin pathway once activated (Colanero et al. 2018). The synergistic effects of the *Aft* and *atv* alleles give rise to complete purple pigmentation of the fruit peel of tomatoes under suitable environmental conditions (Mes et al. 2008; Povero et al. 2011).

The biosynthesis of anthocyanins may require high light activation, making it tightly connected with light signalling and one of the most studied effects of photomorphogenesis in plants (Araguirang and Richter 2022; Ma et al. 2021; Yang et al. 2022). This is also the case of *Aft*/*atv* purple tomatoes, which can accumulate diverse amounts of anthocyanins in their peel according to light intensity (Mes et al. 2008; Gonzali et al. 2009).

ELONGATED HYPOCOTYL 5 (HY5) and CONSTITUTIVELY PHOTOMORPHOGENIC 1 (COP1) are two major nuclear regulators of light signalling that act downstream of photoreceptors (Jarillo and Cashmore 1998; Hoecker 2017; Xu 2019; Cañibano et al. 2021). HY5 is a bZIP TF, which plays a crucial role in light signalling by mediating photoreceptor responses to promote photomorphogenesis: it accumulates upon light exposure and targets many light responsive genes by binding to their promoters (Gangappa and Botto 2016; Xiao et al. 2022). In *Arabidopsis thaliana* HY5 can up‐ or downregulate almost 3000 different genes, including many of the structural and regulatory genes of the anthocyanin pathway, thus leading to many light‐mediated physiological responses and stimulating anthocyanin production (Shin et al. 2013). The positive effect exerted by HY5 on anthocyanin synthesis was also described in other species (Li et al. 2012; Liu et al. 2018; Qiu et al. 2019). In Arabidopsis COP1 is an E3 ubiquitin ligase that acts as a negative regulator of photomorphogenesis by degrading most of the positive modulators of light signalling, such as HY5 and HY5 HOMOLOGUE (HYH), through ubiquitination and subsequent proteolysis under dark conditions (Ang et al. 1998; Holm et al. 2002; Osterlund et al. 2000). The function of COP1 as ubiquitin ligase is impeded by light, thus leading to the accumulation of TFs that promote light responses. COP1 functions together with SUPPRESSOR OF PHYA‐105 (SPA) proteins within a protein complex called COP1‐SPA (Zhu et al. 2008; Hoecker 2017). For the COP1‐SPA complex to perform its E3 ubiquitin ligase activity, the presence of a second complex, named CDD in plants and composed of the proteins CONSTITUTIVELY PHOTOMORPHOGENIC 10 (COP10), DE‐ETIOLATED 1 (DET1) and DAMAGED DNA BINDING‐PROTEIN 1 (DDB1) is needed (Jarillo and Cashmore 1998; Yanagawa et al. 2004). The CDD complex may act as a substrate adaptor module for a multilevel CULLIN 4 (CUL4)‐based E3 ligase complex, and mutations in any of its components also result in nonworking COP1‐SPA complexes (Yanagawa et al. 2004; Delker et al. 2014; Gangappa and Kumar 2018; Ponnu and Hoecker 2021).

A well‐known class of tomato mutants, which are characterized by high pigment content and photomorphogenic phenotypes, includes the *high pigment 1 (hp1)* and *high pigment 2 (hp2*) lines, which exhibit high levels of flavonoids, carotenoids and chlorophylls. *hp1* and *hp2* mutants represent knockout alleles of the *DDB1* and *DET1* genes, respectively (Mustilli et al. 1999; Lieberman et al. 2004). Mutations in either *DDB1* or *DET1* confer hypersensitivity to light alongside photomorphogenesis‐related phenotypes, such as higher chlorophyll, flavonoid and carotenoid contents, shorter hypocotyls, and alterations in the expression of several light‐related genes (Levin et al. 2006; Sestari et al. 2014).

*ddb1* and *det1* mutants with similar phenotypes due to exaggerated light responses have also been isolated in *Arabidopsis thaliana* (Cañibano et al. 2021; Das et al. 2021). In this species, *det1* mutants have been shown to harbour nonworking COP1‐SPA complexes, resulting in the accumulation of COP1 targets, such as HY5 (Cañibano et al. 2021; Das et al. 2021). Recently, Cañibano et al. (2021) demonstrated how DET1 promotes HY5 degradation and turnover through a direct association with COP1. COP1, which is mostly renowned for its dark‐responsive activities, can also respond to increased temperatures by being partially relocated into the nucleus. This shows that light and temperature signals converge to COP1, and additively function to control its activity (Park et al. 2017; Nieto et al. 2022). At warm temperatures, COP1, relocated into the nucleus, can destabilize HY5, accelerating its breakdown irrespective of light: HY5 and COP1 together thus produce a light‐controlled and a thermoregulated switch, acting on many HY5 downstream processes, including the anthocyanin biosynthetic pathway (Delker et al. 2014; Maier and Hoecker 2015; Park et al. 2017; Cañibano et al. 2021; Bian et al. 2022).

Climate change and, in particular, global warming impact not only on the yield of crops but also on their quality, typically by altering plant secondary metabolism, which produces chemical substances such as phenolics, terpenoids, and alkaloids, which can affect food quality as nutrients and can also be used in plant‐based medicine or as chemical sources (Ahmed et al. 2019; Srivastava et al. 2021; Qaderi et al. 2023). While certain phenolic compounds have a beneficial impact on plants under heat stress and can therefore increase in quantity under such conditions, others, including many flavonoids, may respond unfavourably and decrease in quantity. For instance, flavonols such as quercetin and kaempferol may undergo breakdown under high temperatures (Ahmed et al. 2019; Qaderi et al. 2023). The impacts extend to terpenoid metabolism and alkaloid synthesis, each of which involves individual reactions to heat stress. In tea cultivation, temperature fluctuations inversely correlate with tea quality and influence catechins and antioxidant compounds (Ahmed et al. 2019). The metabolic pathways and gene regulation of Arabidopsis plants are modified during temperature stress (Howarth and Ougham 1993; Larkindale et al. 2005; Florian et al. 2014). The influence of temperature on the ripening of grape berries is multidimensional: the increase in sugar content induced by high temperatures during ripening is indeed accompanied by the alteration of secondary metabolites, particularly anthocyanins, phenolics and aroma compounds, which drastically decrease (Van Leeuwen et al. 2018; Rienth et al. 2021). Understanding these variations is crucial for anticipating and regulating the implications of climate change on crop growth and secondary metabolite production.

Low temperatures usually induce the accumulation of anthocyanins in plants due to the beneficial role of these compounds as free radical scavengers: therefore, the regulation of the synthesis of these pigments has often been studied under cold temperatures (Zhang et al. 2011; Zhang et al. 2019). Also in purple tomatoes the accumulation of anthocyanins is positively affected by cool temperatures during ripening, and some of the TFs which act as positive regulators of the pathway are induced by cold (Kiferle et al. 2015; Qiu et al. 2016). No studies have been conducted so far in warm conditions: thus, the threat of global warming on the quality of purple tomatoes is still unexplored and could counteract all the breeding efforts made to develop these novel lines. This prompted us to study how high temperatures can impact the synthesis of anthocyanins in tomato fruits to address a possible climate‐related problem.

## 2 Methods

### 2.1 Plant Materials and Growth Conditions

The tomato genotypes *Aft/Aft* x *atv/atv (Aft/atv)* and *Aft/Aft* x *atv/atv* x *hp2/hp2 (Aft/atv/hp2)* in the MicroTom background were used. The seeds of *Aft/atv/hp2* were kindly donated by Prof. L.E.P. Peres from Universidade de São Paulo (Brazil) (Sestari et al. 2014). The seeds of *Aft/atv* and *hp2* were obtained by backcrossing *Aft/atv/hp2* with the cv. MicroTom and selecting for the *Aft/atv* and the *hp2* genotypes in the segregating F2 and F3 generations. The seeds of the novel *Aft/atv/hp2* tomato line with large fruits were obtained by crossing the donor MicroTom line *Aft/atv/hp2* with the recurrent female parent line *Aft/atv ‘*SunBlack’ described by Mazzucato et al. (2013). Seeds were germinated in rock‐wool plugs (Grodan, https://www.grodan.com/) soaked in a nutritive solution (Kiferle et al. 2013). Two‐week‐old seedlings were transplanted in pots containing a 70:30 soil/expanded clay mixture and placed: (i) for control tests, in a growth chamber with 23°/20°C day/night temperature, 12 h photoperiod, 110 µmol photons m^−2^ s^−1^, and 40% relative humidity; ii) for warm tests, in a growth chamber with 30°/26°C day/night temperature, 12 h photoperiod, 110 µmol photons m^−2^ s^−1^, and 40% relative humidity. For *A. thaliana* experiments, seeds of *Col‐0* background and of *cop1‐6* mutant line were placed in a 70:30 soil/expanded clay mixture, vernalized in the dark for 48 h at 4°C, and then germinated in a growth chamber with 23°/20°C day/night temperature, 8 h photoperiod, 150 µmol photons m^−2^ s^−1^, and 40% relative humidity. For *A. thaliana* seedlings experiments, seeds were sterilized for 1 min in 70% ethanol followed by 1 min in 20% bleach, rinsed six times with sterile water and then placed in Petri dishes onto half‐strength MS media pH 5.7 supplemented with 90 mM glucose. Seeds were vernalized in the dark for 48 h at 4°C and then germinated in the growth chamber for Arabidopsis plants above described. The *Aft/atv/hp2* line in SunBlack genotype and the SunBlack line (Povero et al. 2011) were grown under the same conditions used for MicroTom plants but under a light intensity of 210 µmol photons m^−2^ s^−1^.

### 2.2 Gene Cloning and Plasmid Construction

Full‐length coding sequences (CDS) of tomato *Solyc12g005950* (*COP1 homolog*) (AAC98912.1), *Solyc11g011980* (*COP1‐like isoform X1*) (XP_004250243.1), both long and short transcripts (PQ041227), and *Solyc08g061130* (*HY5*) were amplified by PCR using the ‘Phusion High‐Fidelity DNA Polymerase' (Thermo Fisher Scientific, https://corporate.thermofisher.com) and the oligonucleotide primers reported in Supporting Information S1: Table S1, starting from RNA extracted from the fruit peel using the ‘Spectrum Plant Total RNA Kit’ (Merck, https://www.merckgroup.com), treated with DNase and reverse‐transcribed with SuperScript IV Reverse Transcriptase (Thermo Fisher Scientific). The promoters of tomato *Solyc09g091510* (*CHS1*), *Solyc05g053550* (*CHS2*), *Solyc02g083860* (*F3H*), and *Solyc09g065100* (*AN1*) were amplified by PCR using the ‘Phusion High‐Fidelity DNA Polymerase’ and the oligonucleotide primers reported in Supporting Information S1: Table S1, starting from DNA extracted from the fruit peel using the ‘Wizard Genomic DNA Purification Kit’ (Promega). The amplicons were individually cloned and inserted into the pENTR/d‐TOPO vector (Thermo Fisher Scientific) and sequenced (Eurofins Genomics https://eurofinsgenomics.eu/). The promoters of *AN2like* and *DFR* were obtained in previous studies (Kiferle et al. 2015; Colanero et al. 2020). The entry clones were recombined with different destination vectors via Invitrogen Gateway recombination cloning technology (Thermo Fisher Scientific) as described below. Multiple sequence alignments were performed using ClustalW (https://www.ebi.ac.uk/Tools/msa/clustalo) sequence analysis software.

### 2.3 Phylogenetic Analysis

Nucleotide and protein sequence similarities were analysed with the Blast tool from *Solgenomics.org*. Phylogenetic analysis was performed on the Phylogeny.fr platform (http://www.phylogeny.fr/index.cgi) (Dereeper et al. 2008) using default programs and parameters. MUSCLE was used for multiple sequence alignment, and PhyML was used for phylogenetic analysis.

### 2.4 Protein Structure Modelling

Protein structures were assessed bioinformatically by using the SWISS‐MODEL service (Guex et al. 2009; Bienert et al. 2017; Waterhouse et al. 2018), which operates through homology modelling. Briefly, amino acid sequences of the proteins of interest were used to automatically identify template models in the available database. Models of the proteins of interest were then built based on the most similar proteins found, and the models with the highest accuracy were chosen. Models for COP1 homolog and COP1‐like isoform X1 were built based on the AlphaFold (Varadi et al. 2022) v2 DB models of *A. thaliana* COP1_ARATH (ATCOP1) and *Solanum chilense* A0A6N2BFB5.1. A (RING‐type domain‐containing protein), respectively.

### 2.5 Anthocyanin Quantification

Fifty micrograms of fruit peel were ground and extracted overnight in 300 µL of HCl 1% (v/v) in methanol. Extracts were recovered, diluted with 200 µL of distilled water, and one volume of chloroform was added to remove chlorophylls through mixing and centrifugation (2 min at 14 000x*g*). Anthocyanin‐containing aqueous phase was recovered and, after addition of 600 µL of an aqueous solution containing 40% HCl 1% (v/v) in methanol, absorbances at 530 and 657 nm were determined spectrophotometrically and subtracted. Anthocyanins were expressed as mg petunidin‐3‐(p‐coumaroyl)‐rutinoside‐5‐glucoside g^−1^ fresh weight. Mean values were calculated from six biological replicates (obtained by pooling different fruits from different plants) for *Aft/atv* and *Aft/atv/hp2* plants in cv. MicroTom, and from four biological replicates (obtained by sampling one fruit from a different plant for each replicate) for the *Aft/atv/hp2* line in SunBlack genotype and the SunBlack line. Only the light exposed upper part of the fruit peel was sampled.

### 2.6 Yeast One‐Hybrid Assay (Y1H)

Y1H was performed using the ‘Grow “n” Glow One‐Hybrid System’ (Mobitech #GNGK03) according to the manufacturer instructions, but with modifications to the structures of the original plasmids. Briefly, pGNG2 bait vector containing the *GFPuv* gene and the pJG4‐5 prey vector containing the *B42* activation domain were first made Gateway compatible. For pGNG2, vector was cut using appropriate restriction enzymes, as reported in Supporting Information S1: Table S6; a Gateway compatible cassette containing the *ccdB* and *chloramphenicol resistance* genes flanked by ATTR1 and ATTR2 sites was amplified from the p2GWL7 plasmid (Karimi et al. 2002) using the primers reported in Supporting Information S1: Table S6; then, the Gateway cassette was directly cut with the appropriate restriction enzymes and ligated to obtain the pGNG2_GW plasmid. For pJG4‐5, the Gateway cassette was first mutagenized through overlapping PCR using the primers reported in Supporting Information S1: Table S6 to remove an internal restriction site; next it was cut with the appropriate restriction enzymes and ligated with the pJG4‐5 to obtain the pJG4‐5_GW plasmid. The *AD‐HY5* was produced by recombining the entry vector containing the CDS of *HY5* into the destination vector pJG4‐5_GW. The plasmids *pCHS1:GFP*, *pCSH2:GFP*, *pF3H:GFP*, *pDFR:GFP*, *pAN2like:GFP* and *pAN1:GFP* were produced by recombining the entry vector containing the relative CDS into the destination vector pGNG2_GW. Yeast was transformed using the LiAc/SS carrier DNA/PEG method (Gietz and Schiestl 2007). For quantitative Y1H assay each transformed yeast strain obtained was inoculated in liquid media and grown overnight at 30°C, then back diluted to OD 1.0, centrifuged at 15 000x*g* for 30 s, the supernatant discarded, and the pellet resuspended in sterile water to a final OD of 1.0. The GFP level of each yeast strain was quantified using a TriStar Microplate Reader (Berthold).

### 2.7 Transient Transformation of Tomato Protoplasts

Leaf protoplasts were isolated following the protocol of Shi et al. (2012) from 3‐week‐old MicroTom wild‐type (WT) and *hp2* plants, grown as reported above. Polyethylene glycol‐mediated protoplast transformation was carried out as described in Yoo et al. (2007).

### 2.8 Degradation Assays in Tomato Protoplasts

Degradation assays with the dual‐luciferase system were performed with the *Renilla reniformis* (Renilla) and *Photinus pyralis* (Firefly) luciferase (Luc) enzymes. The *35S:COP1 homolog*, *35S:COP1‐like isoform X1 long*, and *35S:COP1‐like isoform X1 short* effector constructs were produced as reported in Colanero et al. (2018), with the CDS cloned in this study. A reporter construct consisting of a *Firefly Luc‐tagged HY5* under the control of the *35S* promoter (*35S:HY5‐Firefly_Luc*) was produced by recombining the entry vector containing the CDS of *HY5* into the destination vector p2GWL7 (Karimi et al. 2002). The *35S:Renilla_Luc* vector was used to normalize the luminescence values detected in the protoplasts (Weits et al. 2014). Effector and reporter plasmids were cotransfected in protoplasts (5 µg for each effector plasmid, 5 µg for the *35S:HY5‐Firefly_Luc* reporter construct and 2.5 µg of the *35S:Renilla_Luc* construct), and incubated overnight under dark. Protoplasts were lysed and Firefly and Renilla luciferase activities were sequentially measured through the ‘Dual‐Luciferase Reporter Assay System’ (Promega) with a Lumat LB 9507 Tube Luminometer (Berthold Technologies, NY, USA). Data were expressed as relative luciferase units (RLU = Firefly_Luc/Renilla_Luc).

### 2.9 Interaction Assays in Tomato Protoplasts

Interaction assays with the split‐luciferase system were performed with Renilla Luc enzyme reconstituted from the N‐terminal and C‐terminal halves codified by the Renilla *NLuc* and *CLuc* CDS. The *35S:COP1 homolog‐NLuc*, *35S:COP1‐like isoform X1 long‐NLuc* and *35S:COP1‐like isoform X1 short‐NLuc* constructs were produced by recombining the entry vector containing the relative CDS into the destination vector pDuExAc6 (Kato and Jones 2010). The *35S:CLuc‐HY5* construct was produced by recombining the entry vector containing the *HY5* CDS into the destination vector pDuExDn6 (Kato and Jones 2010). Effector plasmids were cotransfected in protoplasts (5 µg for each plasmid) and incubated overnight under dark. Luminescence levels were measured using the ‘Renilla Luciferase Assay System’ (Promega) and a Lumat LB 9507 Tube Luminometer (Berthold Technologies) and normalized to the protein content of each sample determined through the Bradford protein assay (Bio‐Rad). Data were expressed as relative luciferase activity µg^−1^ protein (RLU/µg protein). The vectors pDuExAc6 and pDuExDc6 (Kato and Jones 2010), cotransfected together or in different combinations with the effector constructs, were used as negative controls (Figure 4D, Supporting Information S1: Figure S7).

### 2.10 RNA Isolation, cDNA Synthesis, and Quantitative RT‒PCR Analysis

**Figure 1:**
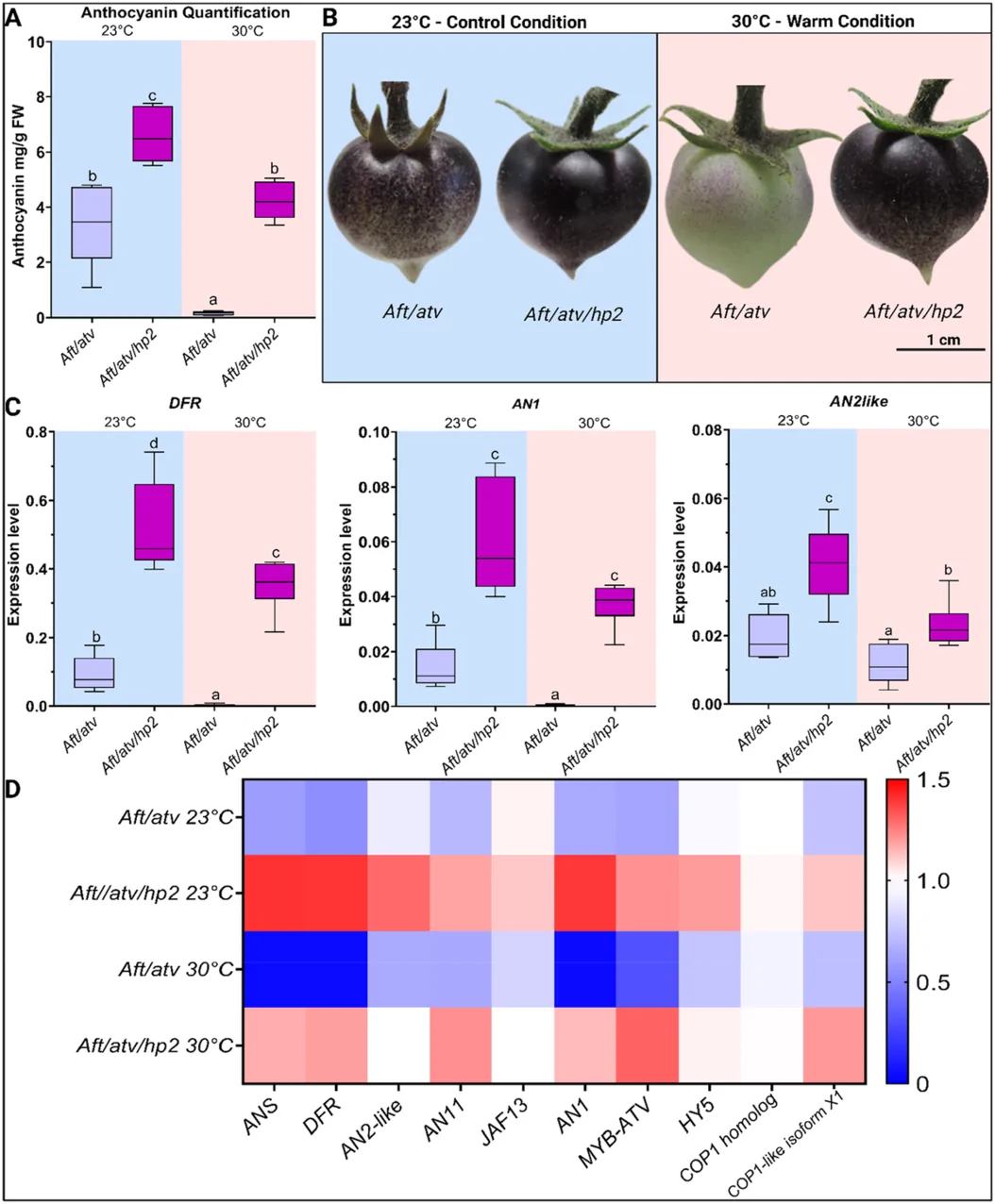
Anthocyanin synthesis in tomato fruit is repressed by high temperatures. (A) Anthocyanin quantification in peel from fruits at the mature green stage of Aft/atv and Aft/atv/hp2 lines grown at control (23°C) and warm (30°C) temperatures. (B) Phenotypes of Aft/atv and Aft/atv/hp2 fruits at control and warm temperatures. (C) Gene expression analysis in peel from fruits at the mature green stage of Aft/atv and Aft/atv/hp2 lines grown at 23°C and 30°C. Anthocyanin biosynthetic (DFR) and regulatory (AN1, AN2‐like) genes were analysed. (D) Gene expression analysis of biosynthetic (ANS, DFR) and regulatory (AN2, AN2‐like, AN11, JAF13, AN1, MYB‐ATV) genes of the anthocyanin pathway and of genes involved in photomorphogenesis (HY5, COP1 homolog, COP1‐like isoform X1) in peel from fruits as in C. For each gene, expression data were normalized by dividing each value by the global mean of all samples, followed by Log2(x+1) transformation. The corresponding bar plots with statistical analyses for each gene are shown in Supporting Information S1: Figure S2. Expression levels are represented as a heatmap by the colour and intensity of the boxes according to the colour scale. In C and D, data are means of six biological replicates ± SD. In C, letters indicate statistically significant differences as assessed by ANOVA and Tukey's post‐hoc test, *p* ≤ 0.05.

Total RNA extracted from the peel of fruits at the mature green stage with the ‘Spectrum Plant Total RNA Kit’ (Merck) was reverse‐transcribed into cDNA using the ‘Maxima First Strand cDNA Synthesis Kit for RT‒qPCR, with dsDNase’ (Thermo Fisher Scientific). Quantitative RT‒PCR was performed with a QuantStudio 3 Real‐Time PCR system (Applied Biosystems, Thermo Fisher Scientific) using the ‘PowerUp SYBR Green Master Mix’ (Thermo Fisher Scientific) and the primers listed in Supporting Information S1: Table S2. The expression levels of each target gene relative to those of the reference genes *Elongation Factor 1‐alpha* (*EF1A*) and *ACTIN*‐2 (*ACT*‐2) were quantified using the geometric averaging method (geNorm) (Vandesompele et al. 2002). Data reported in the heatmap in Figure 1F and in the bar plot in Supporting Information S1: Figure S2 were normalized as follows: for each gene, expression data were normalized by dividing the value of each biological replicate by the global mean of all samples, followed by Log2(x+1) transformation. Data were then displayed as relative expression levels.

### 2.11 Cellular Localization of COP1 Proteins

The entry vectors containing the CDS of *COP1 homolog COP1‐like isoform X1 long* and *COP1‐like isoform X1 short* were recombined with the Gateway compatible destination vector p2FGW7 (Karimi et al. 2002). Protoplasts were isolated as described, transformed with 5 µg of DNA for each plasmid, and incubated in the dark at 23°C or 30°C for 16 h before subsequent analysis. For light‐induced relocation assays, after 16 h in the dark, the protoplasts were moved to light conditions, consisting of 110 µmol photons m^−2^ s^−1^ either at 23°C or 30°C for 6 h before analysis. *35S:NLS‐mCherry*, obtained by recombining the *NLS‐mCherry* sequence from Shukla et al. (2019) into the p2GW7 vector, was used as a nuclear marker. The fluorescence of GFP and RFP was imaged immediately after removing the samples from the incubator with a Nikon Eclipse Ti‐5 video‐confocal microscope (Nikon, https://www.nikon.it) using suitable filters.

### 2.12 Arabidopsis Floral Dipping for Complementation Assays

COP1 complementation assays were performed by stable transformation of the *A. thaliana COP1* knockout *(cop1*‐*6)* mutant with the CDS of each of the two *COP1* genes from *S. lycopersicum*. Plants were transformed through the floral dip method as described by Zhang et al. (2006) with the pO7WG2 vector (Hamedeh 2019) containing the CDS of *COP1 homolog* or *COP1‐like isoform X1 long*. Seeds from dipped plants were screened for RFP signals with a Nikon Eclipse Ti‐5 video confocal microscope (Nikon) using suitable filters. RFP‐positive seeds were used for phenotyping and genotyping experiments with the primers listed in Supporting Information S1: Table S5.

### 2.13 Protein Extraction and Nuclear‐Cytoplasmatic Separation

Protein extraction and cellular fractionation were performed as described by Law et al. (2013) with some adjustments. 120 mg of frozen plant material was ground using liquid nitrogen, transferred to a 50 mL Falcon tube, mixed with 6 mL g^−1^ of NIB buffer (60 mM HEPES pH 8.0, 1 M sucrose, 5 mM KCl, 5 mM MgCl_2_, 5 mM EDTA, 0.6% Triton‐X100, 1 mM PMSF, 1 mM pepstatin, 1 miniComplete/10 mL) and homogenized with a frozen spatula. The solution was filtered through two layers of Miracloth (Millipore‐Sigma) into a new Falcon tube and centrifuged at 2790×*g* at 4°C for 20 min. The supernatant, consisting of the cytoplasmic fraction, was discarded. The pellet was resuspended by gently swirling in 1 mL of EB2 buffer (0.25 M sucrose, 10 mM Tris‐HCl pH 8, 10 mM MgCl_2_, 1 mM EDTA, 1% Triton‐X100, 5 mM BME, 1 mM PMSF, 1 mM pepstatin, 1 miniComplete/10 mL), transferred to a 1.5 mL tube and centrifuged at 7000×*g* for 10 min at 4°C. The supernatant was discarded, and the last steps of resuspension and centrifugation were repeated two more times to remove the remaining cytoplasmic fractions. The pellet was resuspended by mixing in 100 µL of NLB buffer (50 mM Tris‐HCl pH 8, 10 mM EDTA, 1% SDS, 1 mM PMSF, 1 mM pepstatin, 1 miniComplete/10 mL), and centrifuged once more at 11 000x*g* for 2 min. The supernatant, consisting of the nuclear‐enriched‐lysate fraction, which was clear of debris and DNA, was collected and stored at −80°C for further experiments.

### 2.14 Western blot Immunostaining

Total protein content was quantified using the ‘Pierce BCA Protein Assay Kit’ (Thermo Fisher Scientific). Twenty micrograms of nuclear extracts were denatured at 95°C for 5 min in XT Sample Buffer (Bio‐Rad) and 0.8 M DTT. The samples were loaded onto polyacrylamide gels (NuPage Bis‐Tris Gels, Thermo Fisher Scientific), separated through SDS‐PAGE, and transferred onto a PVDF membrane (Bio‐Rad) using the TransBlot Turbo Transfer System (Bio‐Rad). The membranes were incubated with rabbit polyclonal anti‐HY5 (PhytoAB PHY0748S) or anti‐H3 (Elabscience E‐AB‐22081) antibodies, which were all diluted 1:1000. HRP‐conjugated goat anti‐rabbit IgG (Agrisera AS09 602) or HRP‐conjugated rabbit anti‐mouse IgG (Merck Millipore AP160A) were used as the secondary antibody. Clarity Max Western ECL Substrate (Bio‐Rad) and a ChemiDoc MP Imaging System (Bio‐Rad) were used to detect signals. Image Lab software (Bio‐Rad) was used to quantify the intensity of the detected bands. The HY5 quantification data from each biological replicate were normalized to the bands corresponding to histone H3, which was used as a housekeeping and localization marker.

### 2.15 Stable Transformation and Regeneration of Tomato

CRISPR/Cas9 vectors were generated using Golden Gate entry vectors from Lowder et al. (2015) and the Gateway compatible binary vector pK7WG2 (Karimi et al. 2002) with the guide RNA described in Supporting Information S1: Table S3. *Agrobacterium tumefaciens* cells from the GV3101 (MP90) strain hosting the recombinant binary vector were used to transform MicroTom *Aft*/*atv* plants with the protocol described in Zuluaga et al. (2008). Transgenic regenerating plants were selected for kanamycin resistance and the presence of the pK7WG2 construct detected by PCR using the oligonucleotides listed in Supporting Information S1: Table S4. Positive *hy5* knockout lines were identified by amplification and sequencing of a 300 bp genomic fragment of *HY5* containing the supposed cut site for the guide RNA using the primers reported in Supporting Information S1: Table S4. The editing output and efficiency were calculated with the Inference of CRISPR Edits (ICE) tool from Synthego (https://ice.synthego.com/).

### 2.16 Statistics

Statistical analyses were performed with GraphPad Prism 10.00 (www.graphpad.com/scientific-software/prism/). The data were analysed by *t* tests or one‐way ANOVA, with differences measured using Tukey's honest significant difference (HSD) multiple comparisons test.

### 2.17 Figure Preparation

Figures were created with BioRender.com.

## 3 Results

### 3.1 Anthocyanin Biosynthesis in Tomato Fruit Peel is Abolished at High Temperatures

To study the effects of temperature on anthocyanin production in purple tomato lines, we grew *Aft/atv* plants, which can produce purple fruits, under two different temperature conditions. In fruits grown at 23°C, set as the ‘control condition’ in our experimental set‐up, anthocyanin biosynthesis resulted switched on and led to the development of visible purple pigmentation at the mature green stage, particularly concentrated in the upper areas of the peel, which were more exposed to light. On the other hand, at 30°C, here defined as the ‘warm condition’, anthocyanin biosynthesis in the same fruits was strongly inhibited (Figure 1A,B, Supporting Information S1:Figure S1). This high temperature‐induced repression was accompanied by the downregulation of the expression of various anthocyanin biosynthetic and regulatory genes (Figure 1C,D), such as the LBGs *DFR* and *ANS*, the *R2R3‐MYB* regulator *AN2‐like*, and the bHLH‐encoding factor *AN1. AN2‐like* and *AN1* encode two proteins essential for activating the pathway in tomato fruit through the MBW complex (Colanero et al. 2020). Other genes which resulted downregulated at 30° C were the *bHLH JAF13*, and the negative *R3‐MYB* regulator *MYB‐ATV* (Figure 1D). Overall, these data revealed a substantial negative influence exerted by high temperatures on the anthocyanin pathway.

Tomato fruits obtained from *Aft/atv/hp2* plants, harbouring the *hp*2 mutation, displayed a two‐fold increase in anthocyanin content compared with those of *Aft/atv* plants when both lines were grown at 23°C, also showing an almost uniform pigmentation of the epicarp (Figure 1A,B). Remarkably, the *Aft/atv/hp2* fruits also displayed a strong tolerance against the inhibitory effects of high temperature on anthocyanin production, showing a 25‐fold increase in anthocyanin content with respect to the *Aft/atv* fruits when the two lines were grown at 30°C (Figure 1A,B). The analysis of the expression levels of the regulatory and biosynthetic genes of the anthocyanin pathway confirmed the phenotypic data, showing that at 23°C the expression of these genes was significantly higher in the *Aft/atv/hp2* fruits than in the *Aft/atv* ones, and the same pattern was maintained, although with expressions partially shifted downwards, at 30°C (Figure 1C,D).

**Figure 2:**
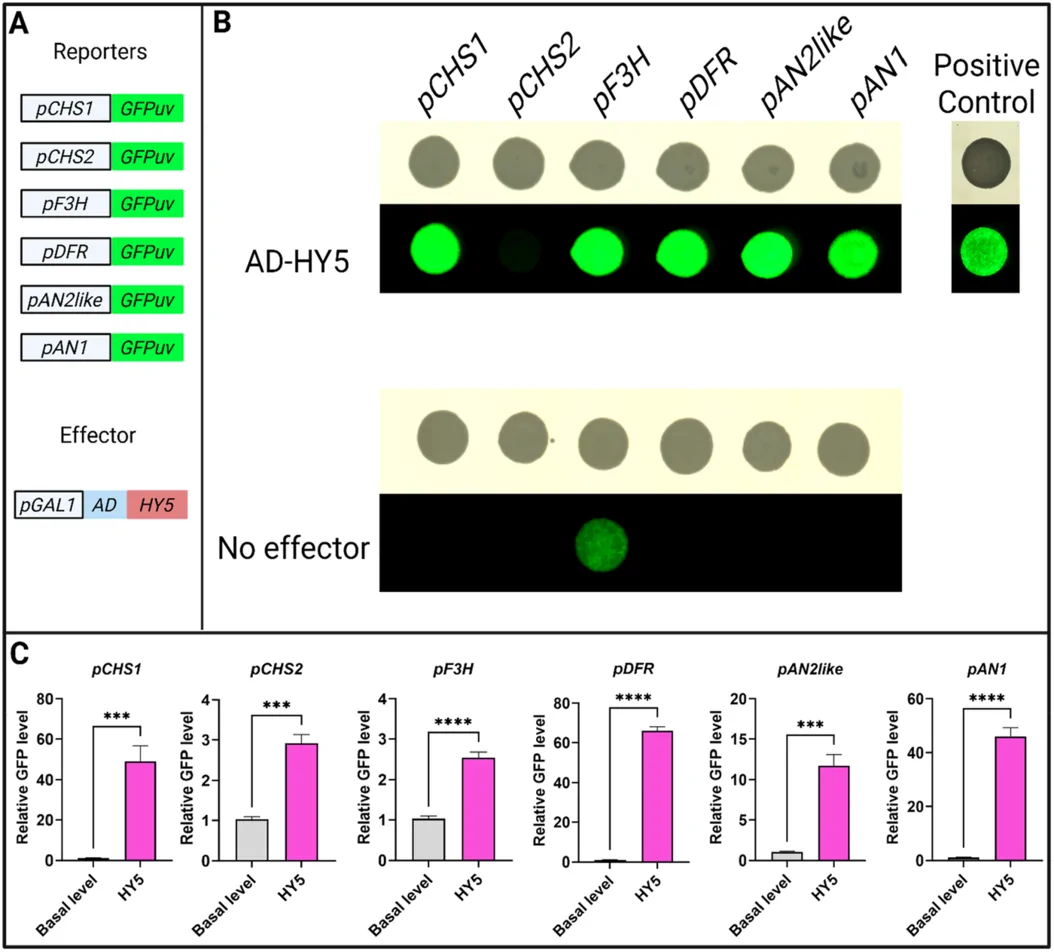
HY5 binds promoters of anthocyanin related genes**.** Yeast one‐hybrid assay of HY5 against the promoters of anthocyanin related genes. (A) Reporter constructs containing promoter sequences of CHS1, CHS2, F3H, DFR, AN2like and AN1 fused to the GFPuv gene and effector construct consisting of HY5 fused to the B42 activation domain. (B) EGY48 yeast strain transformed with the corresponding reporter and effector constructs and grown on SD/‐U/‐T or SD/‐U. Yeast colonies were then photographed under white light and under GFP exciting wavelength. Yeast transformed with only reporter constructs were used as negative controls. (C) Quantitative yeast one‐hybrid assay on the obtained transformed yeast colonies grown in liquid SD/‐U/‐T or SD/‐U media and quantified for GFP level with a plate reader. Data are expressed as relative GFP level with the basal value of the reporter set to 1 and are means of three biological replicates ± SD. Unpaired *t*‐test was carried out and *** and **** asterisks indicate significant differences (*p* ≤ 0.001 and *p* ≤ 0.0001, respectively). [Color figure can be viewed at wileyonlinelibrary.com]

The *hp2* mutation leads to several changes in the physiology of the plant, in part due to the supposed increased stability of HY5 which confers hyperphotomorphogenic responses and consequent higher anthocyanin content. HY5 is known to bind specific ACE and G‐box elements in the promoters of its targets (Jungeun et al. 2007 *;* Song et al. 2008). To confirm this, we set up a Y1H assay to test whether HY5 can physically bind to the promoters of genes involved in anthocyanin synthesis, all containing several HY5‐binding sites (Supporting Information S1: Figure S3). Reporter constructs, consisting of promoters of anthocyanins related genes (*CHS1*, *CHS2*, *F3H*, *DFR*, *AN2like*, *AN1)* driving the expression of *GFP*, were cotransfected in yeast with an effector construct consisting of *HY5* fused with the *B42* activation domain (Figure 2A). Transformed yeast colonies were screened for GFP fluorescence (Figure 2B) and inoculated in selective liquid media, in which GFP levels were quantified (Figure 2C). The results showed that HY5 was clearly able to bind to the promoter of *CHS1, DFR, AN2like* and *AN1*, with activation levels ranging from 15‐ to 70‐ fold increase compared to the basal level of the relative promoters. The promoter of *F3H* showed to be subject to autoactivation in yeast cells and only reduced additional activation by HY5, while the promoter of *CHS2* was activated by HY5 at barely detectable levels.

### 3.2 COP1, a Negative Regulator of HY5, Is Duplicated in Tomato

Since the only difference between the tomato genotypes under study was the *hp2* mutation, we focused our attention on two key regulators of photo‐ and thermomorphogenesis, COP1 and HY5. By using the Arabidopsis COP1 (AtCOP1) sequence as a reference, a BLAST search revealed two possible homologous proteins in *S. lycopersicum*, namely'COP1 homolog’ (Solyc12g005950*)* and ‘COP1‐like isoform X1’ (Solyc11g011980*)*. A third tomato protein, COP1‐like (Solyc11g005190), was found in the literature as another possible homolog (Liu et al. 2004).

**Figure 3:**
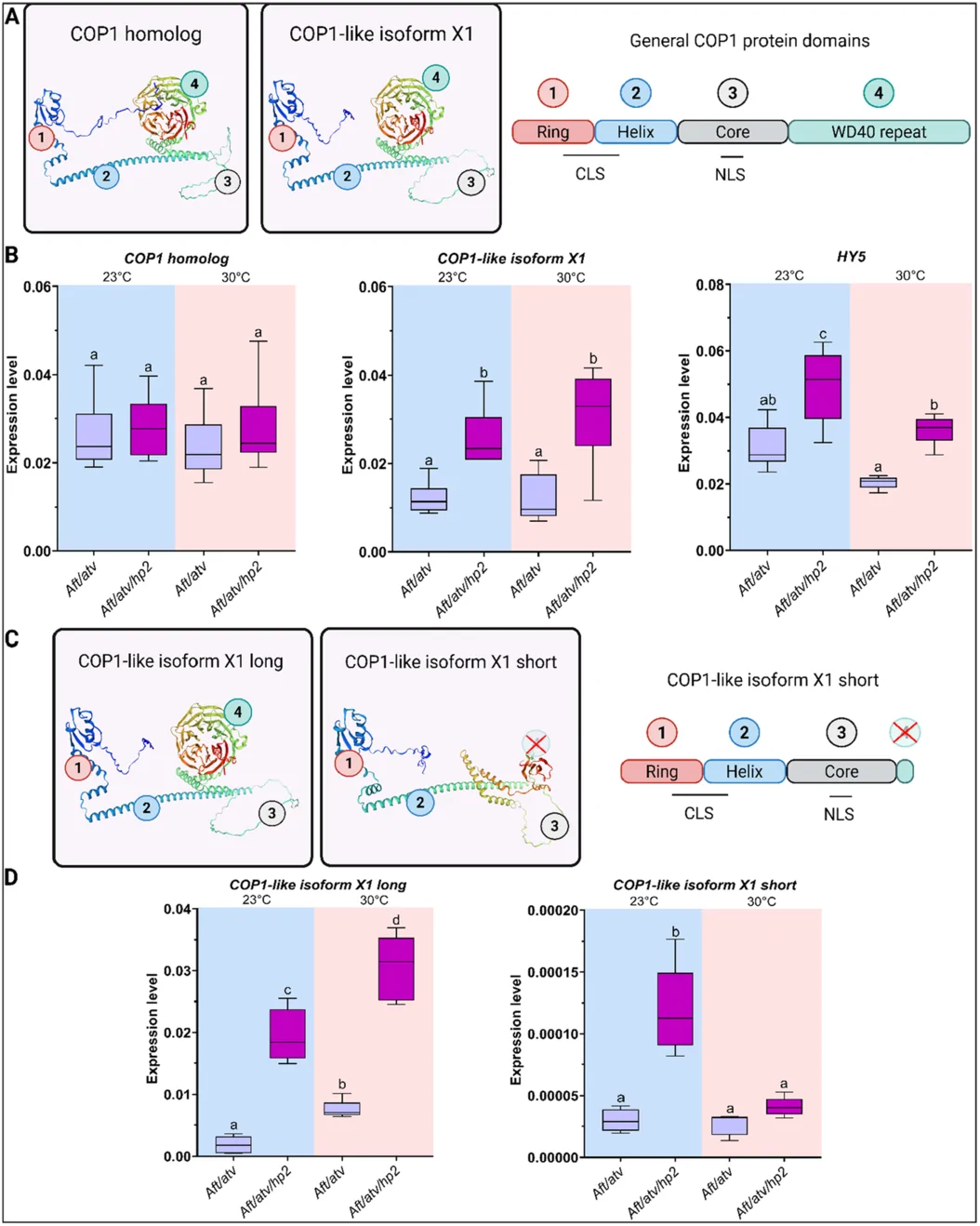
The expressions of COP1 homolog and HY5 are not altered by high temperatures in *S. lycopersicum*. (A) *S. lycopersicum* harbours two copies of COP1, named COP1 homolog and COP1‐like isoform X1, coding for highly similar proteins (left) showing the typical domains of *Arabidopsis thaliana*'s COP1 factor (right). (B) Gene expression levels of COP1 homolog, COP1‐like isoform X1 and HY5 in peel from fruits at the mature green stage of Aft/atv and Aft/atv/hp2 lines grown at 23°C and 30°C. (C) COP1‐like isoform X1 undergoes alternative splicing, producing a long canonical transcript and a short one (left), which produces a polypeptide that completely loses the WD40 domain (right). (D) Gene expression levels of COP1‐like isoform X1 long and COP1‐like isoform X1 short in tomato fruit peel of Aft/atv and Aft/atv/hp2 lines grown at 23°C and 30°C. In B and D, data are means of six biological replicates ± SD. Letters indicate statistically significant differences as assessed by ANOVA and Tukey's post‐hoc test, *p* ≤ 0.05. [Color figure can be viewed at wileyonlinelibrary.com]

An in‐depth phylogenetic analysis, backed by BLAST and protein modelling, led us to conclude that only the first two proteins are similar to AtCOP1, suggesting that they are possible AtCOP1 counterparts (Figures 3A, Supporting Information S1: Figure S1). COP1 homolog and COP1‐like isoform X1 are indeed members of the WD40 protein family, showing the typical WD40 repeats as well as a high similarity of the Ring, Helix and Coil domains with those of AtCOP1 (Figure 3A, Supporting Information S1: Figures S1 and S4). Further structural inspections offered evidence validating COP1‐like as a homolog of AtRUP1 (Supporting Information S1: Figure S4), a hypothesis also supported by functional characterization (Yang et al. 2022). In accordance with these data, we focused on *COP1 homolog* and *COP1‐like isoform X1* genes.

The expression of *COP1 homolog* did not differ in the fruit peel under different temperatures or genotypes (Figures 1D and 3B). On the other hand, *COP1‐like isoform X1* expression was dependent on the presence of the *hp2* mutation: the *Aft/atv/hp2* line consistently manifested a twofold increase in *COP1‐like isoform X1* expression compared to that of the *Aft/atv* line, irrespectively of temperature (Figures 1D and 3B). Similarly, the expression levels of *HY5* were twofold greater in the fruits of *Aft/atv/hp2* than in those of *Aft/atv*, and in both cases only slightly reduced by the increase of temperature (Figures 1D and 3B).

Interestingly, when the CDS of *COP1‐like isoform X1* was cloned from cDNA, a second, shorter transcript was detected. We deduced from sequencing and sequence alignments that this shorter variant likely originated from an alternative splicing process that removed parts of exons 7 and 9 and the entire exon 8 (Supporting Information S1: Figure S6). The resulting CDS was predicted to produce, due to a premature stop codon, a shorter protein characterized by the loss of most of the WD40 domain (Figures 3C and Supporting Information S1: Figure S5). When considering the individual expression levels of the two transcripts of *COP1‐like isoform X1*, the *hp2* mutation enhanced the expression of the long transcript, which was also more expressed at 30°C in both genotypes, whereas the short one, always transcribed at very low levels, at 23°C was more expressed in the *Aft/atv/hp2* fruits than in the other line and negatively affected by temperature in the presence of the *hp2* mutation (Figure 3D).

### 3.3 Tomato COP1 Factors Regulate Anthocyanin Biosynthesis Through the Degradation of HY5

We examined the capacity of the putative tomato COP1 proteins to degrade HY5. To perform its functions, COP1 requires some cofactors, which, if mutated, inhibit the functionality of all COP1‐SPA complexes. One of these cofactors is DET1, which is nonfunctional in the *hp2* mutants. We thus set up a transactivation assay using MicroTom WT protoplasts, which correctly expressed all the cofactors of COP1, and *hp2* protoplasts, which produced a nonworking DET1 protein (Levin et al. 2006; Delker et al. 2014). Luc‐tagged HY5 was co‐expressed with each of the possible COP1 factors ‐ COP1 homolog, COP1‐like isoform X1 long, or its alternative splicing variant COP1‐like isoform X1 short.

**Figure 4:**
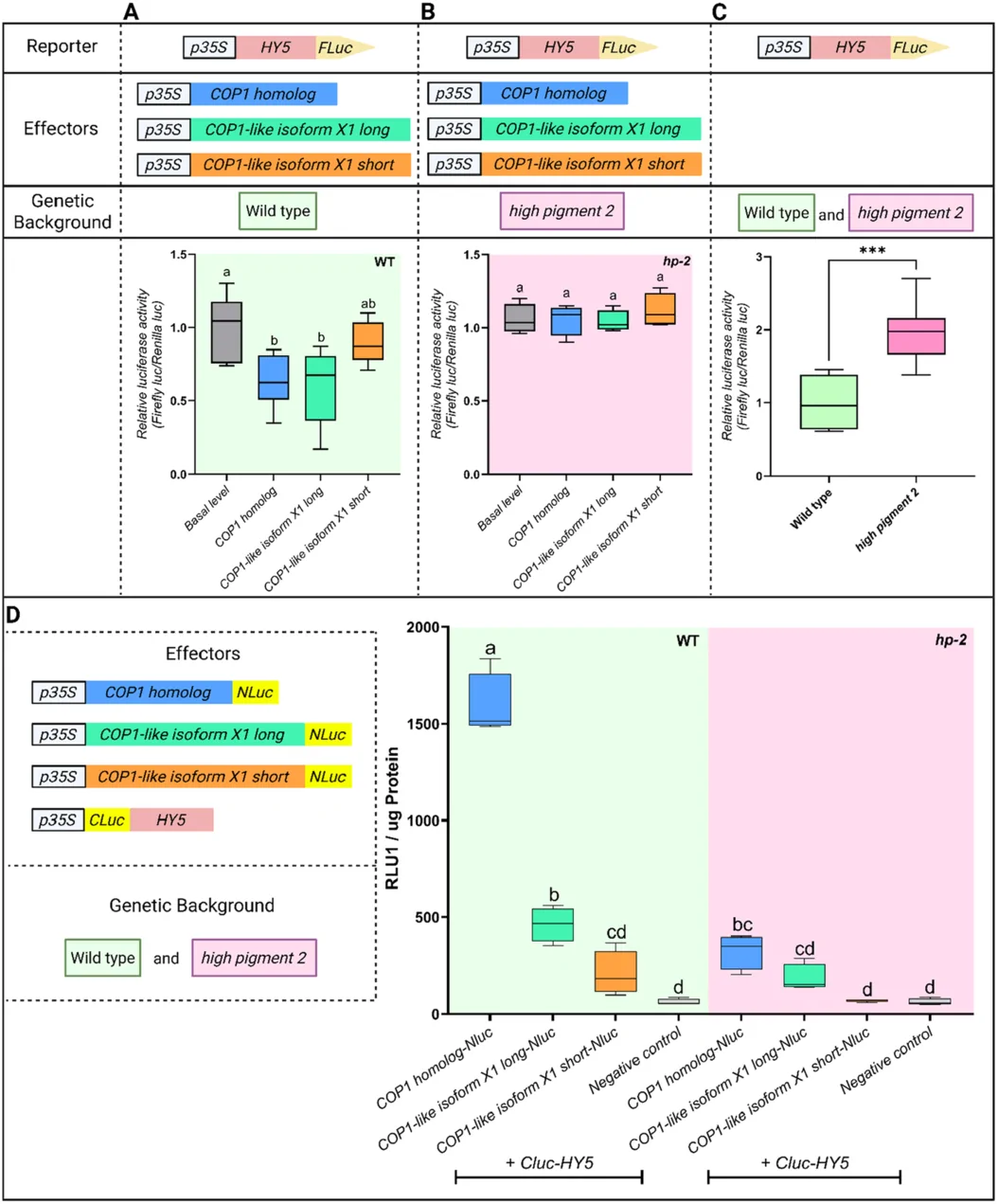
Tomato COP1 proteins require DET1 to destabilize HY5 and interact with it. Scheme recapitulating the protoplast dual‐luciferase assays showing the reporter and effector plasmids and the genetic backgrounds of the protoplasts used. Relative luciferase activity of HY5‐LUC reporter when co‐expressed with different tomato COP1 proteins in WT (A) and hp2 (B) protoplasts. Data are expressed as relative luciferase activity (RLU) (Firefly_Luc/Renilla_Luc) with the basal level of the HY5‐LUC reporter set to 1, and are means of eight biological replicates ± SD. One‐way ANOVA with Tukey's HSD post‐hoc test was performed. Different letters indicate significant differences at *p* ≤ 0.05. (C) Relative luciferase activity of HY5‐LUC reporter when expressed alone in WT and hp2 protoplasts. Data are expressed as RLU with the value of the HY5‐LUC reporter in WT protoplasts set to 1 and are means of eight biological replicates ± SD. Unpaired *t*‐test was carried out and *** asterisks indicate significant difference (*p* ≤ 0.001). (D) Split‐luciferase assay in tomato protoplasts to detect the interaction of the different tomato COP1 proteins with HY5. Data are expressed as RLU, with the Renilla level normalized on the total content of proteins and the value of the negative control set to 1, and are means of four biological replicates ± SD. One‐way ANOVA with Tukey's HSD test was performed. Different letters indicate significant differences at *p* ≤ 0.05. [Color figure can be viewed at wileyonlinelibrary.com]

When expressed in WT protoplasts, both COP1 homolog and COP1‐like isoform X1 long stimulated HY5 degradation, with similar efficacy (Figure 4B). In contrast, the short form of COP1‐like isoform X1 did not change the HY5 levels (Figure 4B). In *hp2* protoplasts, the same assay failed to produce any difference in the amount of HY5: the co‐expression of all COP1 proteins with HY5 indeed had no effect on the luciferase signal (Figure 4C). Furthermore, the basal luciferase activity of the Luc‐tagged HY5 was higher in *hp2* protoplasts than in WT (Figure 4D), suggesting that HY5 resulted more stable in the presence of the mutation *hp2*.

Next, we examined the capacity of the tomato COP1 proteins – COP1 homolog, COP1‐like isoform X1 long, and its alternative splicing variant COP1‐like isoform X1 short ‐ to interact with HY5 in a split‐luciferase assay. Each COP1 protein was fused to the N‐terminal fragment of Renilla luciferase and co‐expressed together with HY5 fused with the C‐terminal fragment of Renilla luciferase. Constructs were expressed both in MicroTom WT and *hp2* protoplasts. When expressed in WT protoplasts, both COP1 homolog and COP1‐like isoform X1 long were able to interact with HY5, with COP1 homolog displaying a greater efficiency, in the order of 3‐4‐fold increase, than COP1‐like isoform X1 long (Figure 4D). The short form of COP1‐like isoform X1 showed the weakest interaction with HY5 (Figure 4D). In *hp2* protoplasts, the same assay resulted in weak interactions with HY5 of all COP1 proteins. COP1 homolog and COP1‐like isoform X1 long were still able to bind to HY5, but with 4‐ and twofold reductions, respectively, in the interaction levels. The short form of COP1‐like isoform X1 was completely unable to interact with HY5, as the luciferase signal was identical to the negative control (Figure 4D).

### 3.4 High Temperature Induces COP1 Nuclear Localization and HY5 Degradation

In Arabidopsis, COP1 activity was shown to be negatively regulated by light, in part through relocation of COP1 to the cytoplasm, a mechanism that allows the accumulation in the nucleus of HY5 and other COP1 targets under light (Subramanian et al. 2004; Lee et al. 2017; Park et al. 2017; Kang et al. 2021). Additional experiments also showed how the nuclear levels of COP1 increase with the onset of high temperatures under light (Park et al. 2017). It is therefore possible that also in tomato COP1 relocation into the nucleus at high temperatures, even under light exposure, triggers the degradation of light‐related factors, such as HY5, thus repressing anthocyanin biosynthesis.

**Figure 5:**
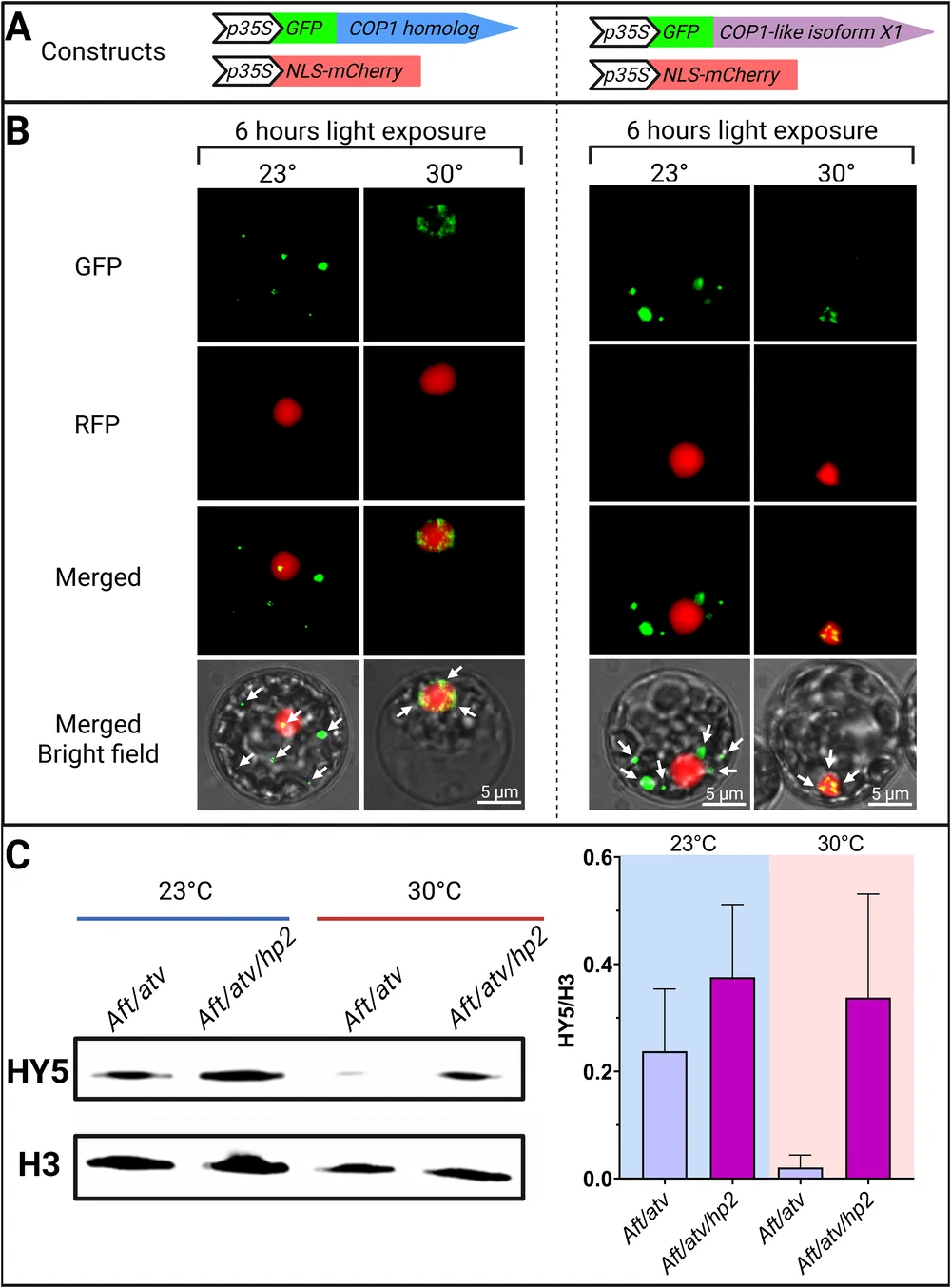
Tomato COP1 proteins are relocated into the nucleus at high temperatures thus destabilizing HY5. (A) GFP‐COP1 homolog and GFP‐COP1‐like isoform X1 fusion proteins were co‐expressed with the nuclear‐localization signal (NLS)‐mCherry protein in tomato protoplasts. (B) GFP, RFP, merged GFP + RFP and merged bright field + GFP + RFP images of protoplasts are shown. White arrows indicate COP1 localization foci. (C) Endogenous HY5 protein levels in nuclear fractions isolated from peel of Aft/atv and Aft/atv/hp2 fruits developed at different temperatures. A representative Western Blot probed with specific antibodies for HY5 and histone H3 is shown on the left. The relative quantification of HY5 protein levels normalized on histone H3 levels is shown on the right. Data are expressed as HY5/H3 and are means of three biological replicates ± SD. The unedited and uncropped membranes of the Western Blot with protein ladders are shown in Supporting Information S1: Figure S9. [Color figure can be viewed at wileyonlinelibrary.com]

To prove this mechanism, the possible relocation of tomato COP1 proteins in response to temperature was assayed. GFP‐tagged COP1 proteins (COP1 homolog or the long isoform of COP1‐like X1) were co‐expressed in protoplasts together with an RFP nuclear marker. At 23°C in the dark, the nuclear localization of both COP1 proteins was evident (Supporting Information S1: Figure S8A,B). However, after 6 h of light exposure at 23°C, a clear subsequent relocation of COP1 factors outside the nucleus was observed (Figure 5B). In contrast, at 30°C, both in the dark and in the light, COP1 homolog and COP1‐like isoform X1 remained nuclear (Figure 5B). While localizing COP1 homolog and COP1‐like isoform X1, it was possible to observe some nuclear speckles and cytoplasmatic bodies typically described in AtCOP1 localization studies. Notably, at 23°C under light we observed a third type of localization of both COP1 homolog and COP1‐like isoform X1, with the proteins distributed throughout the whole nucleus, although with a weak signal (Supporting Information S1: Figure S8C,D).

We quantified the protein levels of HY5, a major COP1 target, to determine whether they changed according to the cellular relocation of COP1 at different temperatures. Western blot analysis was carried out on nuclear protein fractions extracted from peel of *Aft/atv* and *Aft/atv/hp2* fruits grown at 23°C or at 30°C immunostained with anti‐HY5 antibodies. The results indicated extremely low HY5 levels in the nuclear fractions of *Aft/atv* fruits grown at 30°C compared with those present in fruits grown at 23°C (Figure 5C). On the other hand, the *Aft/atv/hp2* fruits showed higher HY5 protein levels at 23°C than the *Aft/atv* fruits at the same temperature, and, more notably, they distinctly showed HY5 in the nuclei at 30°C (Figure 5C).

### 3.5 The Tomato COP1 Proteins Complement COP1 in Arabidopsis and *HY5* Knockout Mimics the High‐Temperature Phenotype

To confirm the functions of tomato COP1 proteins in vivo, we performed a complementation assay in Arabidopsis *cop1‐6* mutants, a widely used knockout line for *AtCOP1* (Stacey et al. 2000; Ma et al. 2002). These mutants have a peculiar phenotype, characterized by photomorphogenic traits, such as the development and opening of cotyledons, even in the dark, strong anthocyanin enrichment, and inhibition of elongation and growth. All these features resemble fusca‐like phenotypes and are attributed to the overaccumulation of HY5 (Stacey et al. 2000).

**Figure 6:**
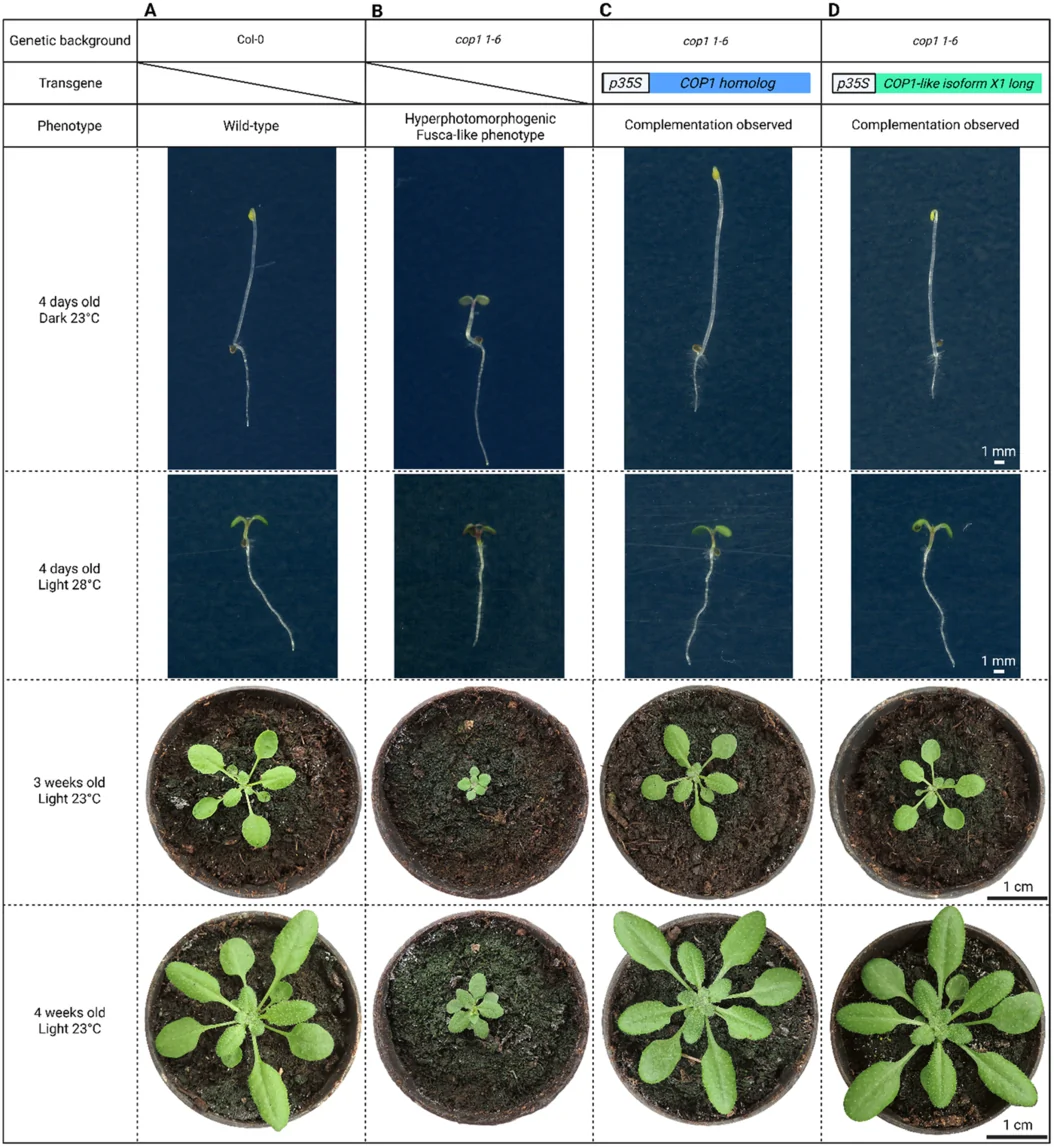
Complementation assay of *Arabidopsis thaliana* cop1‐6 knock‐out (ko) mutants with tomato COP1 homolog and COP1‐like isoform X1. Representative scheme of the complementation assays showing the genetic construct and the background used (Arabidopsis cop1‐6 and wild type Col‐0). 4‐day‐old seedlings, 3‐week‐old and 4‐week‐old wild type Col‐0 plants (A), cop1‐6 plants (B) and cop1‐6 plants complemented with tomato COP1 homolog (C) or COP1‐like isoform X1 long (D) grown under different light and temperature conditions. [Color figure can be viewed at wileyonlinelibrary.com]

**Figure 7:**
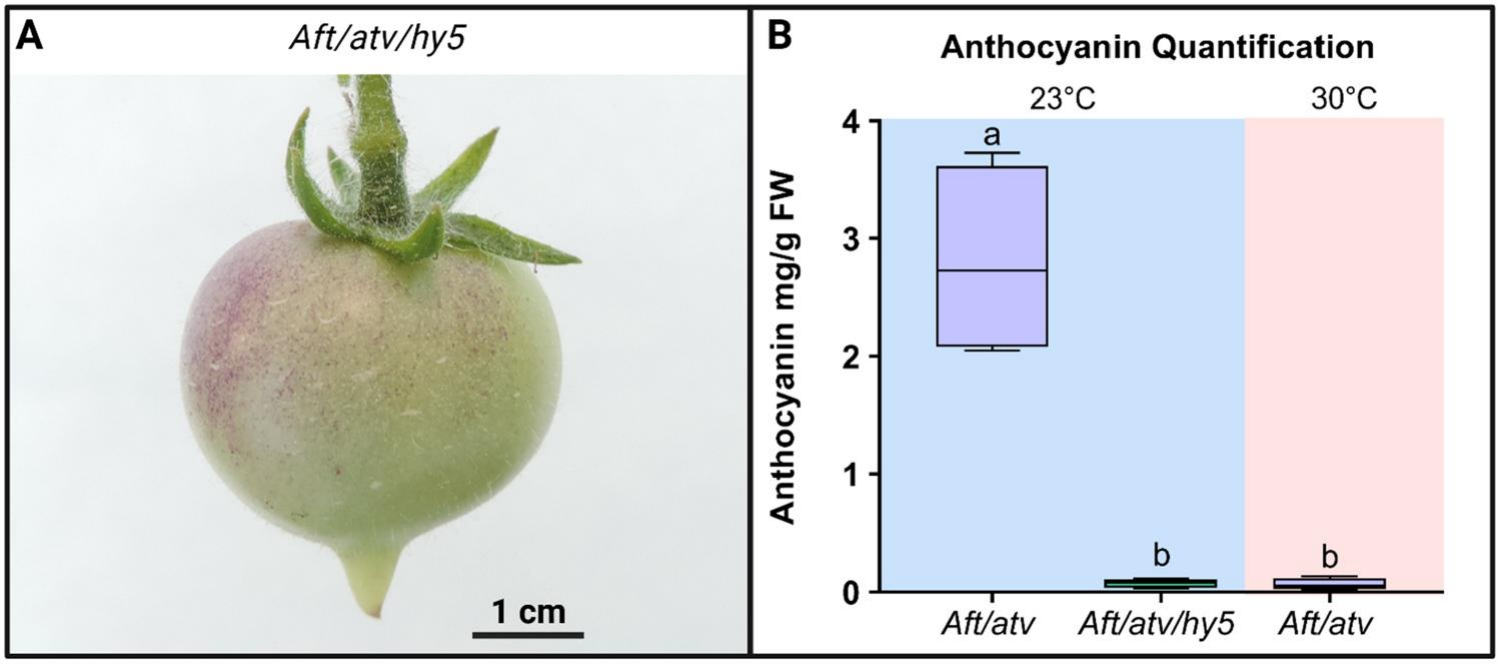
HY5 knockout mimics the high‐temperature phenotype. (A) Representative fruit of an Aft/atv/hy5 plant at the mature green stage grown at control temperatures (23°C). (B) Anthocyanin quantification in peel from fruits at the mature green stage of Aft/atv and Aft/atv/hy5 plants grown at 23°C and of Aft/atv plants grown at warm temperatures (30°C). Data are means of four biological replicates ± SD; statistically significant differences were assessed by ANOVA and Tukey's post‐hoc test, *p* ≤ 0.05. [Color figure can be viewed at wileyonlinelibrary.com]

Constructs containing either the CDS of *COP1 homolog* or the CDS of *COP1‐like isoform X1 long*, the two tomato COP1 forms able to degrade HY5, were ectopically expressed under the *35S* promoter in Arabidopsis *cop1‐6* plants (Figure 6, 7, and Supporting Information S1: Figure S10A–E). Both *COP1 homolog* and *COP1‐like isoform X1*, when expressed in Arabidopsis *cop1‐6* mutants (Supporting Information S1: Figure S10), complemented the endogenous mutation, resulting in plants with a phenotype similar to *Col‐0* (Figure 6B). Complementation was observed both at 4‐day‐old seedling stage or at 3‐ and 4‐week‐old rosette stage (Figure 6A–D). Under dark at 23°C *cop1‐6* seedlings displayed repression of hypocotyl elongation, green and open cotyledons, while both *Col‐0* and COP1‐complemented lines showed elongated hypocotyls and closed yellowish cotyledons. When grown under light and at 28°C, *cop1‐6* seedlings displayed shorter hypocotyls and higher levels of anthocyanins in both hypocotyl and cotyledons, whereas *Col‐0* and COP1‐complemented lines showed longer hypocotyls and more light‐green tone. When grown at 23°C under light, 3‐ and 4‐week‐old *cop1‐6* plants were very small, while *Col‐0* and COP1‐complemented plants developed as normal.

To prove that the loss of HY5 was responsible for the temperature‐related loss of anthocyanins, we generated stable *HY5* knockout tomato lines*. Aft/atv* plants were stably transformed with a CRISPR/Cas9 vector containing a guide RNA designed to target the *HY5* sequence. The transformants were regenerated and screened for the presence of mutations in *HY5*, identifying a *hy5* knockout line in the *Aft/atv* background with a single nucleotide deletion in the first exon, producing a frameshift and a premature stop codon with the loss of COP1 interaction domain and of the b‐ZIP motif in the corresponding polypeptide (Supporting Information S1: Figure S11A–E). The *Aft/atv/hy5* plants produced pale green fruits with a slower maturation process, and, most interestingly, the loss of HY5 led to an almost complete loss of anthocyanins in the fruit peel even in plants grown at 23° (Figure 7A,B). The few anthocyanins still present mostly accumulated as small purple spots in the upper part of the peel exposed to light. *Aft/atv/hy5* fruits had a final anthocyanin content comparable to that produced by the *Aft/atv* fruits developed at 30°C (Figure 7A,B).

### 3.6 Towards the Development of a Novel Purple Tomato Line Featuring the *hp2* Mutation

**Figure 8:**
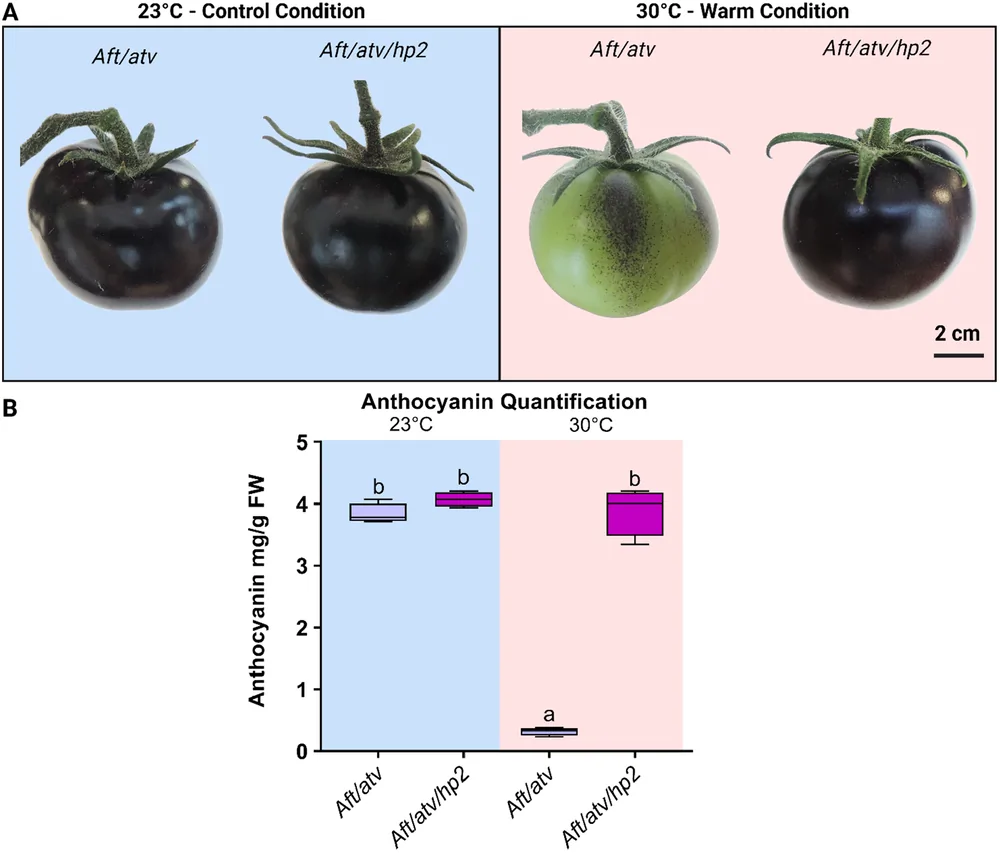
A novel line harbouring the hp2 mutation accumulates anthocyanins even at high temperatures. (A) Phenotypes of Aft/atv and Aft/atv/hp2 fruits in the SunBlack genotype (Mazzucato et al. 2013) grown at control and warm temperatures under a light intensity of 210 µmol photons m^−2^ s^−1^. (B) Anthocyanin quantification in peel from fruits at the mature green stage collected from the plants described in (A). Data are means of four biological replicates ± SD; statistically significant differences were assessed by ANOVA and Tukey's post hoc test, *p* ≤ 0.05. [Color figure can be viewed at wileyonlinelibrary.com]

Finally, the *hp2* mutation was introgressed into the ‘SunBlack’ *Aft*/*atv* genotype of purple tomato previously described (Mazzucato et al. 2013). The fruits of the SunBlack line developed at 23°C under a suitable high light intensity were able to accumulate at the mature green stage a large quantity of anthocyanins, comparable to that of the *Aft/atv/hp2* line, and only when grown at 30°C was the inhibitory effect of temperature on the stimulatory effect of light on the synthesis of anthocyanins prevalent. The novel *Aft*/*atv/hp2* line maintained the characteristics of the parental SunBlack, with pigmented large fruits, but the presence of the *hp2* allele conferred the ability to synthesize high quantities of anthocyanins even at high temperatures (Figure 8A, B, Supporting Information S1: Figure S12).

## 4 Discussion

The consequences of climate change and global warming highlight the importance of studying the possible interplay between increasing temperatures and nutritional value of crops (Srivastava et al. 2021). Rising temperatures indeed pose severe challenges to the production of critical secondary metabolites in plants, such as anthocyanins and other phenolic compounds (Ahmed et al. 2019; Qaderi et al. 2023).

To explore the possible implications of high temperatures, we conducted a case study on tomato anthocyanin‐enriched fruits. High‐anthocyanin tomatoes have been obtained both with transgenic (Butelli et al. 2008; United States Department of Agriculture, 2022; US Food and Drug Administration, 2023) and breeding approaches (Gonzali et al. 2009). In the lines produced by breeding, the synthesis of anthocyanins is affected by the environmental conditions, with light and temperature playing major roles. Confirming that, in our study high temperatures negatively impacted on anthocyanin synthesis in an *Aft/atv* tomato line. Whereas fruits grown at 23°C were almost completely purple, anthocyanins were indeed scarce in fruits grown at 30°C (Figure 1A,B). However, the presence of the extra *hp2* mutation in the same genotype allowed to maintain anthocyanin biosynthesis at high temperatures at levels similar to those observed at 23°C (Figure 1A,B).

In the *Aft/atv* fruits grown at 30°C, the expression of most of the regulatory and biosynthetic genes of the anthocyanin pathway tended to decrease compared with the same fruits grown at 23°C, in parallel with the reduced anthocyanin content. Particularly important was the temperature‐dependent downregulation of the key components of the MBW complex that act in tomato fruit peel: AN2‐like, AN11, JAF13 and AN1 (Kiferle et al. 2015; Colanero et al. 2020) (Figure 1D). The reduced activity of the MBW complex caused the downregulation of the biosynthetic genes, such as *DFR* and *ANS* (Figure 1C). A certain reduction in the expression of these genes was also observed in the *Aft/atv/hp2* fruits grown at 30°C in comparison with those grown under control conditions (Figure 1C,D). However, in this line, the expression of all the components of the MBW complex remained significantly high at 30°C, making transcription of anthocyanin biosynthetic genes still sustained (Figure 1C,D).

Photomorphogenesis and thermomorphogenesis represent two different developmental pathways that share a large number of signalling components (Delker et al. 2014). In the tomato *Aft/atv/hp2* line, exacerbate photomorphogenic traits, due to the mutation in *DET1* gene (Mustilli et al. 1999), resulted in stronger anthocyanin pigmentation of the fruit peel, coupled with a significant tolerance to the inhibition of the anthocyanin biosynthetic pathway exerted by high temperatures (Figure 1). This finding suggests the role of DET1 as necessary in the sensitivity of the anthocyanin pathway to high temperatures in tomato fruit.

Several works carried out in *Arabidopsis thaliana* showed how the activity of the COP1‐SPA E3 ubiquitin ligase, which targets the bZIP transcription factor HY5 for proteasomal degradation under dark resulting in suppression of photomorphogenesis, is enhanced by the CUL4‐based E3 ubiquitin ligase CDD complex (Lau and Deng 2012; Nixdorf and Hoecker 2010; Cañibano et al. 2021), constituted by DET1, COP10 and DDB1 (Jarillo and Cashmore 1998; Yanagawa et al. 2004). DET1 was found to favour the interaction between COP1 and HY5, playing thus an important role in the regulation of HY5 by COP1 (Cañibano et al. 2021). Recent studies have also highlighted how different stimuli induced by elevated temperatures are transduced into altered gene expression through the same DET1‐COP1‐HY5 pathway, which thus constitutes a signalling module that can integrate plant photomorphogenesis and thermomorphogenesis (Delker et al. 2014; Gangappa and Kumar 2018).

Photomorphogenic mutants such as *cop1* and *det1* show increased anthocyanin content as direct consequence of elevated HY5 protein levels (Mayer et al. 1996; Oyama et al. 1997; Lau and Deng 2012). In accordance with this, *Aft/atv/hp2* tomato fruits were characterized by increased anthocyanin content compared to *Aft/atv* fruits and, differently from them, the quantity of anthocyanins remained high when temperatures increased from 23°C to 30°C (Figure 1A,B). In support to this hypothesis tomato HY5 was found to be able to bind to the promoters of several anthocyanin related genes, including biosynthetic (*CHS1, CHS2, F3H, DFR*) and regulatory (*AN2like, AN1*) ones, demonstrating a direct effect on the expression of these gene, ad also an additional indirect activity, as the activation of *AN2like* and *AN1* expression would trigger a cascade converging on the MBW complex and reinforcing in turn the transcription of several anthocyanin related genes (Figure 2).

Coherently with the anthocyanin regulatory and biosynthetic genes, the expression of *HY5* was clearly induced in the *Aft/atv/hp2* line compared with the *Aft/atv* one, but only slightly repressed by high temperatures in both the genotypes (Figure 3B). The decrease in the expression level of *HY5 in Aft/atv* fruits at 30°C cannot fully explain the repression of anthocyanin synthesis under this condition, as *HY5* was still clearly expressed at moderate levels. Hence, high temperature may have affected HY5 post‐transcriptionally. In fact, HY5 protein, clearly present in the fruit peel of the *Aft/atv* lines at 23°C, was barely detectable in the same fruits at 30°C, whereas it was present at high levels in the peel of the *Aft/atv/hp2* fruits regardless of temperature (Figure 5C). The presence of HY5 at 23°C and its almost complete absence at 30°C in *Aft*/*atv* fruits could justify the corresponding differences in the activation of both regulatory and structural anthocyanin‐related genes (Figure 1C,D). Similarly, anthocyanin production was also defective in the *Aft/atv/hy5* fruits, in which *HY5* was knocked out: these fruits did not synthesize anthocyanins at an ambient temperature of 23°C, showing fruit phenotypes similar to those of the *Aft/atv* line grown at 30°C (Figure 6).

To confirm the link between *DET1* mutation and HY5 stabilization in *Aft/atv/hp2* tomato fruits, the attention was directed towards COP1. COP1 proteins in *S. lycopersicum* exist as products of two different genes, *COP1 homolog* and *COP1‐like isoform X1* (Figure 3A), similarly to what occurs in the *Rosaceae* family, where such a duplication was already described and appeared to have evolved with specialized roles and separate targets of the two COP1 factors (Sun et al. 2020b). Interestingly, *COP1‐like isoform X1* mRNA undergoes alternative splicing in tomato, producing two different transcripts: one leads to a complete COP1‐like isoform X1 protein, while the other, originating from alternative splicing, is shorter and contains a premature stop codon (Figure 3A). The polypeptide corresponding to the alternative splicing version should lose almost completely the WD40 domain, which contains the specific amino acids required for the interaction with the targets (Supporting Information S1: Figure S5). *COP1 homolog* gene did not show substantial differences in its expression in fruits grown at different temperatures and in the presence of the *hp2* mutation (Figure 3B), suggesting not to be under HY5 control. On the contrary, *COP1‐like isoform X1*, particularly its canonical long CDS, was more highly expressed in the *hp2* background, and thus may be an HY5 target, as *AtCOP1* is in Arabidopsis (Cañibano et al. 2021). Coherently with this hypothesis, the expression of the long transcript of *COP1‐like isoform X1* increased in the *hp2* line. Interestingly, under the experimental conditions of this study, the expression of the short transcript of *COP1‐like isoform X1* was very low and increased only in the *hp2* line under control temperature conditions (Figure 3B).

Both COP1 homolog and COP1‐like isoform X1 (long) were able to interact with HY5 and trigger its degradation, as evidenced by their ectopic expression in WT protoplasts (Figure 4B,D). In *hp2* protoplasts, in contrast, COP1 homolog and COP1‐like isoform X1 displayed much lower interaction efficiencies with HY5 and were not able to trigger its degradation, confirming that in tomato DET1 is required for HY5 interaction and destabilization through COP1 (Figure 4C,D), as shown in Arabidopsis (Cañibano et al. 2021). Although unable to trigger the degradation of HY5, COP1‐like X1 isoform short was still able to weakly interact with HY5 in WT protoplasts: this might be the result of heterodimers producing between full‐length COP1 proteins and COP1‐like isoform X1 short, with only the full‐length subunits being able to interact with HY5. It is known indeed that COP1 proteins may form both homodimers and heterodimers in plants (Ponnu and Hoecker 2021). Despite the apparent differences in the interaction strength with HY5 and in HY5 degradation assay, both COP1 homolog and COP1‐like isoform X1 (long) were able to fully complement the phenotype of *cop1‐6* Arabidopsis plants, either at the seedlings or at the rosette stage (Figure 6), demonstrating to share similar functions when ectopically expressed.

COP1 levels are known to be regulated by several stimuli, including light and temperature (Subramanian et al. 2004; Lu et al. 2015; Park et al. 2017; Nieto et al. 2022). Very importantly, both COP1 homolog and COP1‐like isoform X1 displayed differential subcellular localization based on temperature. While mainly localized outside the nucleus at 23°C, high temperatures triggered the relocation of these proteins to the nucleus (Figure 5B). The translocation of COP1 proteins into the nucleus was thus likely responsible for the degradation of HY5 at 30°C in *Aft*/*atv* fruits even under light exposure. The same relocation did not have major effects on *Aft*/*atv*/*hp2* fruits, as COP1 proteins are not functional in the *hp2* background.

**Figure 9:**
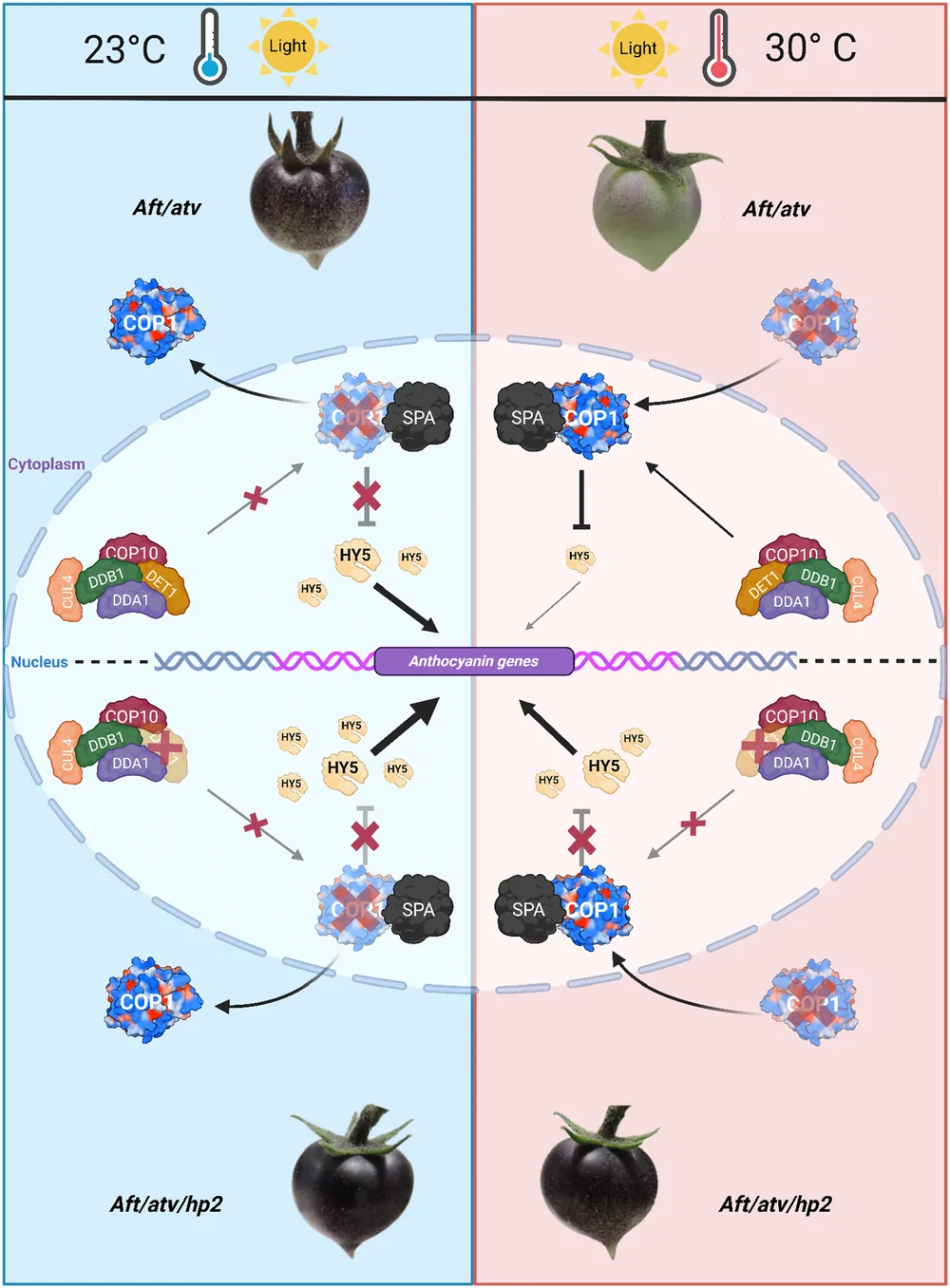
Scheme summarizing the proposed model of temperature‐induced regulation of anthocyanin synthesis in tomato fruits. [Color figure can be viewed at wileyonlinelibrary.com]

A model for anthocyanin biosynthesis in tomato fruits in which HY5, which is induced by light, and COP1, which is differentially localized in the cell according to temperature, act as master regulators is shown in Figure 9. Under light and moderate temperatures, HY5 is present and active in the nucleus and can positively affect as TF anthocyanin synthesis, with COP1 being mainly cytoplasmic, thus preventing HY5 degradation. Higher temperatures instead trigger the relocation of COP1 to the nucleus even under light, leading to its interaction with the SPA proteins and with the CDD complex composed of DET1, CUL4, DDB1 and COP10, which are all nuclear factors. The COP1 network induces HY5 destabilization, with consequent downregulation of anthocyanin biosynthesis. This mechanism is impeded when the *hp2* mutation is present: due to the deactivation of DET1, a key cofactor of COP1 inside the CDD complex, HY5 results more stable, allowing sustained anthocyanin accumulation under light and preventing destabilization at higher temperatures, when COP1 levels are indeed higher in the nucleus but the COP1 network remains inactive because of the nonfunctional DET1 factor.

Exploiting the *hp2* mutation in the *Aft/atv* tomato genotype may thus strongly mitigate the negative impact of high temperatures, maintaining significant anthocyanin production under these conditions. The introgression of the *hp2* mutation in the SunBlack genotype finally resulted in an improved novel tomato line that can sustain prolonged heatwaves without losing the high anthocyanin content of its purple fruits (Figure 8).

In conclusion, the present study elucidates the temperature‐modulated COP1 intracellular dynamics that may regulate anthocyanin production in purple tomato fruits by influencing the stability of HY5. This study also offers insights into the molecular mechanisms driving thermotolerance in this species and, at the same time, a novel strategy to preserve high nutritional value in tomatoes grown under increasing temperature regimens. Future studies aiming at characterizing more in‐depth the molecular mechanisms behind the activity of the different tomato COP1 factors here identified and the regulation exerted by light and temperature on their expression, which appeared to be different in the present study, will allow to understand possible specificities or redundant functions. Analysis of the alternative splicing process of *COP1‐like isoform X1* gene under different environmental conditions and stresses will also help to understand the possible physiological role of the short isoform identified here.

## Conflicts of Interest

The authors declare no conflicts of interest.

## Supporting information

Supplementary material Menconi et al.

## Data Availability

The data that supports the findings of this study are available in the supporting material of this article.

## References

[pce15586-bib-0001] Ahmed, S. , T. S. Griffin , D. Kraner , et al. 2019. “Environmental Factors Variably Impact Tea Secondary Metabolites in the Context of Climate Change.” Frontiers in Plant Science 10: 939. 31475018 10.3389/fpls.2019.00939 PMC6702324

[pce15586-bib-0002] Ang, L.‐H. , S. Chattopadhyay , N. Wei , et al. 1998. “Molecular Interaction Between COP1 and HY5 Defines a Regulatory Switch for Light Control of Arabidopsis Development.” Molecular Cell 1: 213–222. 9659918 10.1016/s1097-2765(00)80022-2

[pce15586-bib-0003] Araguirang, G. E. , and A. S. Richter . 2022. “Activation of Anthocyanin Biosynthesis in High Light—What Is the Initial Signal?” New Phytologist 236: 2037–2043. 36110042 10.1111/nph.18488

[pce15586-bib-0004] Bian, Y. , L. Chu , H. Lin , Y. Qi , Z. Fang , and D. Xu . 2022. “PIFs‐ and COP1‐HY5‐Mediated Temperature Signaling in Higher Plants.” Stress Biology 2: 35. 37676326 10.1007/s44154-022-00059-w PMC10441884

[pce15586-bib-0005] Bienert, S. , A. Waterhouse , T. A. P. de Beer , et al. 2017. The SWISS‐MODEL Repository‐New Features and Functionality. Nucleic Acids Research 45, D313–D319. 10.1093/nar/gkw1132 PMC5210589 27899672

[pce15586-bib-0006] Brugliera, F. , G.‐Q. Tao , U. Tems , et al. 2013. “Violet/Blue Chrysanthemums—Metabolic Engineering of the Anthocyanin Biosynthetic Pathway Results in Novel Petal Colors.” Plant and Cell Physiology 54: 1696–1710. 23926066 10.1093/pcp/pct110

[pce15586-bib-0007] Butelli, E. , L. Titta , M. Giorgio , et al. 2008. “Enrichment of Tomato Fruit With Health‐Promoting Anthocyanins by Expression of Select Transcription Factors.” Nature Biotechnology 26: 1301–1308. 10.1038/nbt.1506 18953354

[pce15586-bib-0008] Cañibano, E. , C. Bourbousse , M. García‐León , et al. 2021. “DET1‐Mediated COP1 Regulation Avoids HY5 Activity Over Second‐Site Gene Targets to Tune Plant Photomorphogenesis.” Molecular Plant 14: 963–982. 33711490 10.1016/j.molp.2021.03.009

[pce15586-bib-0009] Cappellini, F. , A. Marinelli , M. Toccaceli , C. Tonelli , and K. Petroni . 2021. “Anthocyanins: From Mechanisms of Regulation in Plants to Health Benefits in Foods.” Frontiers in Plant Science 12: 748049. 34777426 10.3389/fpls.2021.748049 PMC8580863

[pce15586-bib-0010] Colanero, S. , P. Perata , and S. Gonzali . 2018. “The Atroviolacea Gene Encodes an R3‐MYB Protein Repressing Anthocyanin Synthesis in Tomato Plants.” Frontiers in Plant Science 9: 830. 29971083 10.3389/fpls.2018.00830 PMC6018089

[pce15586-bib-0011] Colanero, S. , P. Perata , and S. Gonzali . 2020. “What's Behind Purple Tomatoes?, Insight Into the Mechanisms of Anthocyanin Synthesis in Tomato Fruits.” Plant Physiology 182: 1841–1853. 31980573 10.1104/pp.19.01530 PMC7140959

[pce15586-bib-0012] Colanero, S. , A. Tagliani , P. Perata , and S. Gonzali . 2020. “Alternative Splicing in the Anthocyanin Fruit Gene Encoding an R2R3 MYB Transcription Factor Affects Anthocyanin Biosynthesis in Tomato Fruits.” Plant Communications 1: 100006. 33404542 10.1016/j.xplc.2019.100006 PMC7747991

[pce15586-bib-0013] Das, S. , V. Garhwal , and S. N. Gangappa . 2021. “DET1 Regulates HY5 Through COP1: A New Paradigm in the Regulation of HY5.” Molecular Plant 14: 864–866. 34048951 10.1016/j.molp.2021.05.023

[pce15586-bib-0014] Delker, C. , L. Sonntag , G. V. James , et al. 2014. “The DET1‐COP1‐HY5 Pathway Constitutes a Multipurpose Signaling Module Regulating Plant Photomorphogenesis and Thermomorphogenesis.” Cell Reports 9: 1983–1989. 25533339 10.1016/j.celrep.2014.11.043

[pce15586-bib-0015] Dereeper, A. , V. Guignon , G. Blanc , et al. 2008. “Phylogeny.Fr: Robust Phylogenetic Analysis for the Non‐Specialist.” Nucleic Acids Research 36: W465–W469. 18424797 10.1093/nar/gkn180 PMC2447785

[pce15586-bib-0016] Florian, A. , Z. Nikoloski , R. Sulpice , et al. 2014. “Analysis of Short‐Term Metabolic Alterations in Arabidopsis Following Changes in the Prevailing Environmental Conditions.” Molecular Plant 7: 893–911. 24503159 10.1093/mp/ssu008

[pce15586-bib-0017] Gangappa, S. N. , and J. F. Botto . 2016. “The Multifaceted Roles of HY5 in Plant Growth and Development.” Molecular Plant 9: 1353–1365. 10.1016/j.molp.2016.07.002. 27435853

[pce15586-bib-0018] Gangappa, S. N. , and S. V. Kumar . 2018. “DET1 and COP1 Modulate the Coordination of Growth and Immunity in Response to Key Seasonal Signals in Arabidopsis.” Cell Reports 25: 29–37.e3. 30282035 10.1016/j.celrep.2018.08.096 PMC6180345

[pce15586-bib-0019] Georgiev, C. 1972. “Anthocyanin Fruit (Aft).” Rep Tomato Genet Coop 22: 10.

[pce15586-bib-0020] Gietz, R. D. , and R. H. Schiestl . 2007. “High‐Efficiency Yeast Transformation Using the LiAc/SS Carrier DNA/PEG Method.” Nature Protocols 2: 31–34. 17401334 10.1038/nprot.2007.13

[pce15586-bib-0021] Glover, B. J. , and C. Martin . 2012. “Anthocyanins.” Current Biology 22: R147–R150. 22401890 10.1016/j.cub.2012.01.021

[pce15586-bib-0022] Gonzali, S. , A. Mazzucato , and P. Perata . 2009. “Purple as a Tomato: Towards High Anthocyanin Tomatoes.” Trends in Plant Science 14: 237–241. 19359211 10.1016/j.tplants.2009.02.001

[pce15586-bib-0023] Gonzali, S. , and P. Perata . 2020. “Anthocyanins From Purple Tomatoes as Novel Antioxidants to Promote Human Health.” Antioxidants 9: 1017. 33092051 10.3390/antiox9101017 PMC7590037

[pce15586-bib-0025] Guex, N. , M. C. Peitsch , and T. Schwede . 2009. “Automated Comparative Protein Structure Modeling With Swiss‐Model and Swiss‐PdbViewer: A Historical Perspective.” Electrophoresis 30, no. Suppl 1: S162–S173. 19517507 10.1002/elps.200900140

[pce15586-bib-0026] Hamedeh, H. 2019. The Response of the Oilseed Crop *Camelina sativa* to Flooding. 10.13140/RG.2.2.23864.42241.

[pce15586-bib-0027] Hoecker, U. 2017. “The Activities of the E3 Ubiquitin Ligase COP1/SPA, a Key Repressor in Light Signaling.” Current Opinion in Plant Biology 37: 63–69. 10.1016/j.pbi.2017.03.015. 28433946

[pce15586-bib-0028] Holm, M. , L.‐G. Ma , L.‐J. Qu , and X.‐W. Deng . 2002. “Two Interacting bZIP Proteins Are Direct Targets of COP1‐Mediated Control of Light‐Dependent Gene Expression in Arabidopsis.” Genes & Development 16: 1247–1259. 10.1101/gad.969702. 12023303 PMC186273

[pce15586-bib-0029] Howarth, C. J. , and H. J. Ougham . 1993. “Gene Expression Under Temperature Stress*.” New Phytologist 125: 1–26. 33874620 10.1111/j.1469-8137.1993.tb03862.x

[pce15586-bib-0030] Jarillo, J. A. , and A. R. Cashmore . 1998. “Enlightenment of the COP1–HY5 Complex in Photomorphogenesis.” Trends in Plant Science 3: 161–163.

[pce15586-bib-0031] Jones, C. M. 2003. “Characterization and Inheritance of the Anthocyanin Fruit (Aft) Tomato.” Journal of Heredity 94: 449–456. 14691311 10.1093/jhered/esg093

[pce15586-bib-0032] Jungeun, L. , H. Kun , S. Viktor , et al. 2007. “Analysis of Transcription Factor HY5 Genomic Binding Sites Revealed Its Hierarchical Role in Light Regulation of Development.” Plant Cell 19, no. Issue 3, (March): 731–749. 10.1105/tpc.106.047688. 17337630 PMC1867377

[pce15586-bib-0033] Kang, C. H. , E. S. Lee , G. M. Nawkar , et al. 2021. “Constitutive Photomorphogenic 1 Enhances ER Stress Tolerance in Arabidopsis.” International Journal of Molecular Sciences 22: 10772. 34639112 10.3390/ijms221910772 PMC8509555

[pce15586-bib-0034] Karimi, M. , D. Inzé , and A. Depicker . 2002. “Gateway™ Vectors for Agrobacterium‐Mediated Plant Transformation.” Trends in Plant Science 7: 193–195. 11992820 10.1016/s1360-1385(02)02251-3

[pce15586-bib-0035] Kato, N. , and J. Jones . 2010. “The Split Luciferase Complementation Assay.” Methods in Molecular Biology 655: 359–376. 20734273 10.1007/978-1-60761-765-5_24

[pce15586-bib-0036] Kiferle, C. , E. Fantini , L. Bassolino , et al. 2015. “Tomato R2R3‐MYB Proteins SlANT1 and SlAN2: Same Protein Activity, Different Roles.” In PLOS ONE, edited by H. Ezura , 10), e0136365. 26308527 10.1371/journal.pone.0136365 PMC4556288

[pce15586-bib-0037] Kiferle, C. , S. Gonzali , H. T. Holwerda , R. R. Ibaceta , and P. Perata . 2013. “Tomato Fruits: A Good Target for Iodine Biofortification.” Frontiers in Plant Science 4: 205. 10.3389/fpls.2013.00205. 23818889 PMC3694224

[pce15586-bib-0038] Larkindale, J. , J. D. Hall , M. R. Knight , and E. Vierling . 2005. “Heat Stress Phenotypes of Arabidopsis Mutants Implicate Multiple Signaling Pathways in the Acquisition of Thermotolerance.” Plant Physiology 138: 882–897. 15923322 10.1104/pp.105.062257 PMC1150405

[pce15586-bib-0039] Lau, O. S. , and X. W. Deng . 2012. “The Photomorphogenic Repressors COP1 and DET1: 20 Years Later.” Trends in Plant Science 17: 584–593. 10.1016/j.tplants.2012.05.004. 22705257

[pce15586-bib-0040] Law, J. A. , J. Du , C. J. Hale , et al. 2013. “Polymerase IV Occupancy at RNA‐Directed DNA Methylation Sites Requires SHH1.” Nature 498: 385–389. 23636332 10.1038/nature12178 PMC4119789

[pce15586-bib-0041] Lee, J.‐H. , J.‐H. Jung , and C.‐M. Park . 2017. “Light Inhibits COP1‐Mediated Degradation of ICE Transcription Factors to Induce Stomatal Development in Arabidopsis.” Plant Cell 29: 2817–2830. 29070509 10.1105/tpc.17.00371 PMC5728130

[pce15586-bib-0042] Van Leeuwen, C. , J.‐P. Roby , and L. De Rességuier . 2018. “Soil‐Related Terroir Factors: A Review.” OENO One 52: 173–188.

[pce15586-bib-0043] Levin, I. , C. de Vos , Y. Tadmor , et al. 2006. “High Pigment Tomato Mutants—More Than Just Lycopene (A Review).” Israel Journal of Plant Sciences 54: 179–190.

[pce15586-bib-0044] Li, Y.‐Y. , K. Mao , C. Zhao , et al. 2012. “MdCOP1 Ubiquitin E3 Ligases Interact With MdMYB1 to Regulate Light‐Induced Anthocyanin Biosynthesis and Red Fruit Coloration in Apple.” Plant Physiology 160: 1011–1022. 10.1104/pp.112.199703. 22855936 PMC3461526

[pce15586-bib-0045] Lieberman, M. , O. Segev , N. Gilboa , A. Lalazar , and I. Levin . 2004. “The Tomato Homolog of the Gene Encoding Uv‐Damaged DNA Binding Protein 1 (DDB1) Underlined as the Gene That Causes the High pigment‐1 Mutant Phenotype.” Theoretical and Applied Genetics 108: 1574–1581. 14968305 10.1007/s00122-004-1584-1

[pce15586-bib-0046] Liu, C.‐C. , C. Chi , L.‐J. Jin , J. Zhu , J.‐Q. Yu , and Y.‐H. Zhou . 2018. “The bZip Transcription Factor HY5 Mediates CRY1a‐Induced Anthocyanin Biosynthesis in Tomato.” Plant, Cell & Environment 41: 1762–1775. 10.1111/pce.13171 29566255

[pce15586-bib-0047] Liu, Y. , S. Roof , Z. Ye , et al. 2004. “Manipulation of Light Signal Transduction as a Means of Modifying Fruit Nutritional Quality in Tomato.” Proceedings of the National Academy of Sciences 101: 9897–9902. 10.1073/pnas.0400935101 PMC470770 15178762

[pce15586-bib-0048] Lowder, L. G. , D. Zhang , N. J. Baltes , et al. 2015. “A CRISPR/Cas9 Toolbox for Multiplexed Plant Genome Editing and Transcriptional Regulation.” Plant Physiology 169: 971–985. 26297141 10.1104/pp.15.00636 PMC4587453

[pce15586-bib-0049] Lu, X.‐D. , C.‐M. Zhou , P.‐B. Xu , Q. Luo , H.‐L. Lian , and H.‐Q. Yang . 2015. “Red‐Light‐Dependent Interaction of phyB With SPA1 Promotes COP1–SPA1 Dissociation and Photomorphogenic Development in Arabidopsis.” Molecular Plant 8: 467–478. 25744387 10.1016/j.molp.2014.11.025

[pce15586-bib-0050] Ma, L. , Y. Gao , L. Qu , et al. 2002. “Genomic Evidence for COP1 as a Repressor of Light‐Regulated Gene Expression and Development in Arabidopsis[W].” Plant Cell 14: 2383–2398. 12368493 10.1105/tpc.004416 PMC151224

[pce15586-bib-0101] Ma, Y. , X. Ma , X. Gao , W. Wu , and B. Zhou . 2021. “Light Induced Regulation Pathway of Anthocyanin Biosynthesis in Plants.” International Journal of Molecular Sciences 22: 11116. 10.3390/ijms222011116. 34681776 PMC8538450

[pce15586-bib-0052] Maier, A. , and U. Hoecker . 2015. “COP1/SPA Ubiquitin Ligase Complexes Repress Anthocyanin Accumulation Under Low Light and High Light Conditions.” Plant Signaling & Behavior 10: e970440. 10.4161/15592316.2014.970440. 25482806 PMC4622049

[pce15586-bib-0053] Mayer, R. , D. Raventos , N.‐H. Chua , R. Mayer , D. Raventos , and N. H. Chua . 1996. “det1, cop1, and cop9 Mutations Cause Inappropriate Expression of Several Gene Sets.” Plant Cell 8: 1951–1959. 8953766 10.1105/tpc.8.11.1951 PMC161326

[pce15586-bib-0054] Mazzucato, A. , D. Willems , R. Bernini , et al. 2013. “Novel Phenotypes Related to the Breeding of Purple‐Fruited Tomatoes and Effect of Peel Extracts on Human Cancer Cell Proliferation.” Plant Physiology and Biochemistry 72: 125–133. 23769702 10.1016/j.plaphy.2013.05.012

[pce15586-bib-0056] Mes, P. J. , P. Boches , J. R. Myers , and R. Durst . 2008. “Characterization of Tomatoes Expressing Anthocyanin in the Fruit.” Journal of the American Society for Horticultural Science 133: 262–269.

[pce15586-bib-0057] Mustilli, A. C. , F. Fenzi , R. Ciliento , F. Alfano , and C. Bowler . 1999. “Phenotype of the Tomato *High Pigment‐2* Mutant Is Caused by a Mutation In the Tomato Homolog of *DEETIOLATED1* .” Plant Cell 11: 145–157. 9927635 10.1105/tpc.11.2.145 PMC144164

[pce15586-bib-0058] Nieto, C. , P. Catalán , L. M. Luengo , et al. 2022. “COP1 Dynamics Integrate Conflicting Seasonal Light and Thermal Cues in the Control of Arabidopsis Elongation.” Science Advances 8: eabp8412. 10.1126/sciadv.abp8412. 35984876 PMC9390991

[pce15586-bib-0059] Nixdorf, M. , and U. Hoecker . 2010. “SPA1 and DET1 Act Together to Control Photomorphogenesis Throughout Plant Development.” Planta 231: 825–833. 10.1007/s00425-009-1088-y. 20041285

[pce15586-bib-0060] Osterlund, M. T. , C. S. Hardtke , N. Wei , and X. W. Deng . 2000. “Targeted Destabilization of HY5 During Light‐Regulated Development of Arabidopsis.” Nature 405: 462–466. 10.1038/35013076. 10839542

[pce15586-bib-0061] Oyama, T. , Y. Shimura , and K. Okada . 1997. “The *Arabidopsis HY5* Gene Encodes a bZIP Protein That Regulates Stimulus‐Induced Development of Root and Hypocotyl.” Genes & Development 11: 2983–2995. 10.1101/gad.11.22.2983. 9367981 PMC316701

[pce15586-bib-0062] Park, Y. J. , H. J. Lee , J. H. Ha , J. Y. Kim , and C. M. Park . 2017. “ cop 1 Conveys Warm Temperature Information to Hypocotyl Thermomorphogenesis.” New Phytologist 215: 269–280. 28418582 10.1111/nph.14581

[pce15586-bib-0063] Ponnu, J. , and U. Hoecker . 2021. “Illuminating the COP1/SPA Ubiquitin Ligase: Fresh Insights Into Its Structure and Functions During Plant Photomorphogenesis.” Frontiers in Plant Science 12: 662793. 10.3389/fpls.2021.662793. 33841486 PMC8024647

[pce15586-bib-0064] Povero, G. , S. Gonzali , L. Bassolino , A. Mazzucato , and P. Perata . 2011. “Transcriptional Analysis in High‐Anthocyanin Tomatoes Reveals Synergistic Effect of Aft and atv Genes.” Journal of Plant Physiology 168: 270–279. 20888667 10.1016/j.jplph.2010.07.022

[pce15586-bib-0065] Qaderi, M. M. , A. B. Martel , and C. A. Strugnell . 2023. “Environmental Factors Regulate Plant Secondary Metabolites.” Plants 12: 447. 36771531 10.3390/plants12030447 PMC9920071

[pce15586-bib-0066] Qiu, Z. , H. Wang , D. Li , et al. 2019. “Identification of Candidate HY5‐Dependent and ‐Independent Regulators of Anthocyanin Biosynthesis in Tomato.” Plant and Cell Physiology 60: 643–656. 30597099 10.1093/pcp/pcy236

[pce15586-bib-0067] Qiu, Z. , X. Wang , J. Gao , Y. Guo , Z. Huang , and Y. Du . 2016. “The Tomato *Hoffman's Anthocyaninless* Gene Encodes a bHLH Transcription Factor Involved in Anthocyanin Biosynthesis That Is Developmentally Regulated and Induced by Low Temperatures.” PLoS One 11: e0151067. 26943362 10.1371/journal.pone.0151067 PMC4778906

[pce15586-bib-0068] Rick, C. M. , A. F. Reeves , and R. W. Zobel . 1968. “Inheritance and Linkage Relations of Four New Mutants.” Rep Tomato Genet Coop 18: 34–35.

[pce15586-bib-0069] Rienth, M. , N. Vigneron , P. Darriet , et al. 2021. “Grape Berry Secondary Metabolites and Their Modulation by Abiotic Factors in a Climate Change Scenario–A Review.” Frontiers in Plant Science 12: 643258. 33828576 10.3389/fpls.2021.643258 PMC8020818

[pce15586-bib-0070] Sestari, I. , A. Zsögön , G. G. Rehder , et al. 2014. “Near‐Isogenic Lines Enhancing Ascorbic Acid, Anthocyanin and Carotenoid Content in Tomato (*Solanum lycopersicum* L. cv Micro‐Tom) as a Tool to Produce Nutrient‐Rich Fruits.” Scientia Horticulturae 175: 111–120.

[pce15586-bib-0071] Shi, X. , S. Gupta , and A. M. Rashotte . 2012. “ *Solanum lycopersicum* Cytokinin Response Factor (SlCRF) Genes: Characterization of CRF Domain‐Containing ERF Genes in Tomato.” Journal of Experimental Botany 63: 973–982. 22068146 10.1093/jxb/err325 PMC3254692

[pce15586-bib-0072] Shin, D. H. , M. Choi , K. Kim , et al. 2013. “HY5 Regulates Anthocyanin Biosynthesis by Inducing the Transcriptional Activation of the MYB75/PAP1 Transcription Factor in Arabidopsis.” FEBS Letters 587: 1543–1547. 10.1016/j.febslet.2013.03.037. 23583450

[pce15586-bib-0073] Shukla, V. , L. Lombardi , S. Iacopino , et al. 2019. “Endogenous Hypoxia in Lateral Root Primordia Controls Root Architecture by Antagonizing Auxin Signaling in Arabidopsis.” Molecular Plant 12: 538–551. 30641154 10.1016/j.molp.2019.01.007

[pce15586-bib-0074] Song, Y. H. , C. M. Yoo , A. P. Hong , et al. 2008. “DNA‐Binding Study Identifies C‐Box and Hybrid C/G‐Box or C/A‐Box Motifs as High‐Affinity Binding Sites for STF1 and Long HYPOCOTYL5 Proteins.” Plant Physiology 146, no. 4, (April): 1862–1877. 10.1104/pp.107.113217. 18287490 PMC2287355

[pce15586-bib-0075] Srivastava, A. K. , P. Mishra , and A. K. Mishra . 2021. Effect of Climate Change on Plant Secondary Metabolism: An Ecological Perspective. Evolutionary Diversity as a Source for Anticancer Molecules, 47–76. Elsevier.

[pce15586-bib-0077] Stacey, M. G. , O. R. Kopp , T.‐H. Kim , and A. G. Von Arnim . 2000. “Modular Domain Structure of Arabidopsis COP1. Reconstitution of Activity by Fragment Complementation and Mutational Analysis of a Nuclear Localization Signal in Planta.” Plant Physiology 124: 979–990. 11080276 10.1104/pp.124.3.979 PMC59198

[pce15586-bib-0078] Subramanian, C. , B.‐H. Kim , N. N. Lyssenko , X. Xu , C. H. Johnson , and A. G. Von Arnim . 2004. “The *Arabidopsis* Repressor of Light Signaling, COP1, Is Regulated by Nuclear Exclusion: Mutational Analysis by Bioluminescence Resonance Energy Transfer.” Proceedings of the National Academy of Sciences 101: 6798–6802. 10.1073/pnas.0307964101 PMC404125 15084749

[pce15586-bib-0079] Sun, C. , L. Deng , M. Du , et al. 2020a. “A Transcriptional Network Promotes Anthocyanin Biosynthesis in Tomato Flesh.” Molecular Plant 13: 42–58. 31678614 10.1016/j.molp.2019.10.010

[pce15586-bib-0080] Sun, Y.‐B. , X.‐J. Zhang , M.‐C. Zhong , et al. 2020b. “Genome‐Wide Identification of WD40 Genes Reveals a Functional Diversification of COP1‐like Genes in Rosaceae.” Plant Molecular Biology 104: 81–95. 32621166 10.1007/s11103-020-01026-7

[pce15586-bib-0081] Vandesompele, J. , K. De Preter , F. Pattyn , et al. 2002. “Accurate Normalization of Real‐Time Quantitative RT‐PCR Data by Geometric Averaging of Multiple Internal Control Genes.” Genome Biology 3, no. 7: research0034. 10.1186/gb-2002-3-7-research0034. 12184808 PMC126239

[pce15586-bib-0082] Varadi, M. , S. Anyango , M. Deshpande , et al. 2022. “Alphafold Protein Structure Database: Massively Expanding the Structural Coverage of Protein‐Sequence Space With High‐Accuracy Models.” Nucleic Acids Research 50: D439–D444. 34791371 10.1093/nar/gkab1061 PMC8728224

[pce15586-bib-0083] Vu, A. T. , and J. M. Lee . 2019. “Genetic Variations Underlying Anthocyanin Accumulation in Tomato Fruits.” Euphytica 215: 196.

[pce15586-bib-0084] Waterhouse, A. , M. Bertoni , S. Bienert , et al. 2018. “SWISS‐Model: Homology Modelling of Protein Structures and Complexes.” Nucleic Acids Research 46: W296–W303. 29788355 10.1093/nar/gky427 PMC6030848

[pce15586-bib-0085] Weits, D. A. , B. Giuntoli , M. Kosmacz , et al. 2014. “Plant Cysteine Oxidases Control the Oxygen‐Dependent Branch of the N‐End‐Rule Pathway.” Nature Communications 5: 3425. 10.1038/ncomms4425 PMC3959200 24599061

[pce15586-bib-0086] Winkel‐Shirley, B. 2002. “Biosynthesis of Flavonoids and Effects of Stress.” Current Opinion in Plant Biology 5: 218–223. 11960739 10.1016/s1369-5266(02)00256-x

[pce15586-bib-0087] Xiao, Y. , L. Chu , Y. Zhang , Y. Bian , J. Xiao , and D. Xu . 2022. “HY5: A Pivotal Regulator of Light‐Dependent Development in Higher Plants.” Frontiers in Plant Science 12: 800989. 10.3389/fpls.2021.800989. 35111179 PMC8801436

[pce15586-bib-0088] Xu, D. 2019. “COP1 and BBXs‐HY5‐Mediated Light Signal Transduction in Plants.” New Phytologist 228: 1748–1753. 10.1111/nph.16296. 31664720

[pce15586-bib-0089] Yan, S. , N. Chen , Z. Huang , et al. 2019. “ *Anthocyanin Fruit* Encodes an R2R3‐MYB Transcription Factor, SlAN2‐Like, Activating the Transcription of *SlMYBATV* to Fine‐Tune Anthocyanin Content in Tomato Fruit.” New Phytologist 225: 2048–2063. 31625612 10.1111/nph.16272

[pce15586-bib-0090] Yanagawa, Y. , J. A. Sullivan , S. Komatsu , et al. 2004. “Arabidopsis COP10 Forms a Complex With DDB1 and DET1 in Vivo and Enhances the Activity of Ubiquitin Conjugating Enzymes.” Genes & Development 18: 2172–2181. 15342494 10.1101/gad.1229504 PMC515294

[pce15586-bib-0091] Yang, G. , C. Zhang , H. Dong , et al. 2022. “Activation and Negative Feedback Regulation of *SlHY5* Transcription by the SlBBX20/21–SlHY5 Transcription Factor Module in UV‐B Signaling.” Plant Cell 34: 2038–2055. 35188198 10.1093/plcell/koac064 PMC9048894

[pce15586-bib-0092] Yoo, S.‐D. , Y.‐H. Cho , and J. Sheen . 2007. “Arabidopsis Mesophyll Protoplasts: A Versatile Cell System for Transient Gene Expression Analysis.” Nature Protocols 2: 1565–1572. 17585298 10.1038/nprot.2007.199

[pce15586-bib-0094] Zhang, Q. , J. Zhai , L. Shao , W. Lin , and C. Peng . 2019. “Accumulation of Anthocyanins: An Adaptation Strategy of *Mikania micrantha* to Low Temperature in Winter.” Frontiers in Plant Science 10: 1049. 31555311 10.3389/fpls.2019.01049 PMC6726734

[pce15586-bib-0095] Zhang, X. , R. Henriques , S.‐S. Lin , Q.‐W. Niu , and N.‐H. Chua . 2006. “Agrobacterium‐Mediated Transformation of *Arabidopsis thaliana* Using the Floral Dip Method.” Nature Protocols 1: 641–646. 17406292 10.1038/nprot.2006.97

[pce15586-bib-0096] Zhang, Y. , E. Butelli , and C. Martin . 2014. “Engineering Anthocyanin Biosynthesis in Plants.” Current Opinion in Plant Biology 19: 81–90. 24907528 10.1016/j.pbi.2014.05.011

[pce15586-bib-0097] Zhang, Y. , S. Zheng , Z. Liu , L. Wang , and Y. Bi . 2011. “Both HY5 and HYH Are Necessary Regulators for Low Temperature‐Induced Anthocyanin Accumulation in Arabidopsis Seedlings.” Journal of Plant Physiology 168: 367–374. 10.1016/j.jplph.2010.07.025. 20932601

[pce15586-bib-0098] Zhu, D. , A. Maier , J.‐H. Lee , et al. 2008. “Biochemical Characterization of Arabidopsis Complexes Containing Constitutively PHOTOMORPHOGENIC1 and Suppressor of PHYA Proteins in Light Control of Plant Development.” Plant Cell 20: 2307–2323. 10.1105/tpc.107.056580. 18812498 PMC2570740

[pce15586-bib-0099] Zuluaga, D. L. , S. Gonzali , E. Loreti , et al. 2008. “ *Arabidopsis thaliana* MYB75/PAP1 Transcription Factor Induces Anthocyanin Production in Transgenic Tomato Plants.” Functional Plant Biology 35: 606. 32688816 10.1071/FP08021

