# Supplementary material for: Loss of DET1 in High Pigment 2 Tomato Prevents High Temperature Repression of Anthocyanin Biosynthesis in Fruit Through HY5 Stabilization
Source: Plant Cell Environ. 2025 May 11;49(10):7449–69. doi: 10.1111/pce.15586 (PMC13551638; doi:10.1111/pce.15586)
Supplement: Supplementary file 1 [file PCE-49-7449-s001.docx]

Supplementary Material for

**Loss of DET1 in *high pigment 2* tomato prevents high temperature repression of anthocyanin biosynthesis in fruit through HY5 stabilization**

**Authors:** Jacopo Menconi^1^, Noemi La Monaca^1^, Irene Cataldo^2^, Pietro Maria Niccolini^1^, Pierdomenico Perata^1^ and Silvia Gonzali^1^*

*Author for correspondence

**Affiliations:**

^1^PlantLab, Institute of Plant Sciences, Scuola Superiore Sant'Anna, Via Guidiccioni 8/10, 56017, San Giuliano Terme, Pisa, Italy

^2^Biology Department, University of Pisa, Via L. Ghini 13, 56126, Pisa, Italy

**Supplementary data**

Table S1. List of oligonucleotide primers used for CDS cloning.

Table S2. List of oligonucleotide primers used for qPCR analysis.

Table S3. List of oligonucleotide primers used for *HY5* CRISPR/Cas9-mediated silencing.

Table S4. List of oligonucleotide primers used for genotyping *HY5* knockout lines.

Table S5. List of oligonucleotide primers used for genotyping Arabidopsis transgenic lines overexpressing *COP1 homolog* or *COP1-like isoform X1*.

Table S6. List of oligonucleotide primers and restriction enzymes used for modification of “Grow ‘N’ Glow” vectors

Fig. S1. Anthocyanin synthesis in tomato fruit is repressed by high temperatures.

Fig. S2. Gene expression analysis in fruit peel under different temperatures.

Fig. S3. Promoter sequences of anthocyanin related genes used in the Yeast One-Hybrid (Y1H) assay.

Fig. S4. Phylogenetic analysis of tomato COP1 factors.

Fig. S5. Alignment of Arabidopsis and tomato COP1 protein sequences.

Fig. S6. Alignment of the gene sequence of *COP1-like isoform X1* with the two alternative transcripts identified.

Fig. S7. Additional negative controls for the split-luciferase assay in tomato protoplasts.

Fig. S8. Tomato COP1 proteins localize in the nucleus under dark.

Fig. S9. Representative Western Blot probed with specific antibodies for HY5 and histone H3.

Fig. S10. Complementation assay of *Arabidopsis thaliana cop1-6* knockout mutants with tomato COP1 proteins.

Fig. S11. Screening of *hy5* knockout lines.

Fig. S12. A line harbouring the *hp2* mutation in a SunBlack tomato genotype accumulates anthocyanins at high temperatures.

**Figure legends**

***Figure S1. Anthocyanin synthesis in tomato fruit is repressed by high temperatures.*** *Phenotypes of Aft/atv and Aft/atv/hp2 fruits in the MicroTom background at control and warm temperatures. The full original images, cropped to produce Fig. 1B, are shown here.*

***Figure S2.*** ***Gene expression analysis in fruit peel under different temperatures.*** *Bar plots showing gene expression levels of biosynthetic genes (ANS, DFR), regulatory genes (AN2, AN2-like, AN11, JAF13, AN1, MYB-ATV) of the anthocyanin pathway, and photomorphogenesis-related genes (HY5, COP1 homolog, COP1-like isoform X1) in peel from fruits at the mature green stage of Aft/atv and Aft/atv/hp2 lines grown at warm (30°C) and control temperatures (23°C). For each gene, expression data were normalized by dividing each value by the global mean of all samples for the gene, followed by Log2(x+1) transformation. Data are means of 6 biological replicates ± SD. Letters indicate statistically significant differences as assessed by ANOVA and Tukey’s post-hoc test, P ≤ 0.05.*

***Figure S3. Promoter sequences of anthocyanin related genes used in the Yeast One-Hybrid (Y1H) assay.*** *Promoter sequences of biosynthetic (CHS1, CHS2, F3H, DFR) and regulatory (AN1, AN2like) genes used in the Y1H assay. Known HY5 binding motifs are highlighted in different colours: ACE elements in red, G-BOX in purple.*

***Figure S4.*** ***Phylogenetic analysis of tomato COP1 factors.*** *Phylogenetic tree analysing similarities among the Arabidopsis thaliana WD40 proteins AtCOP1, AtRUP1, and AtRUP2 and tomato WD40 proteins, including SlCOP1 homolog, SlCOP1-like, and SlCOP1-like isoform X1. In the tree, the tomato proteins encoded by the Solyc12g013840, Solyc07063120, Solyc10g011690, and Solyc08g081990 genes encode other members of the WD40 family.*

***Figure S5. Alignment of Arabidopsis and tomato COP1 protein sequences.*** *ClustalW alignment of the tomato proteins COP1-like isoform X1 long, COP1-like isoform X1 short, COP1 homolog and of Arabidopsis thaliana AtCOP1. The symbol * indicates perfect alignment. Red characters represent the cellular localization signal. The nuclear localization signals are highlighted in purple, the ring finger domain is in yellow, the coiled coil domain is in pale blue, and the WD40 domain is in green. The histidine (G) in the AtCOP1 sequence at position 524 (also present in the sequences of COP1-like isoform X1 long and COP1 homolog), which is essential for the interaction with HY5 (Stacey et al., 2000), is in grey.*

***Figure S6. Additional negative controls for the split-luciferase assay in tomato protoplasts****. Data are expressed as relative luciferase activity (RLU) with the Renilla level normalized on the total content of proteins and with the value of the negative control set to 1, and are means of four biological replicates ± SD.*

***Figure S7. Alignment of the gene sequence of COP1-like isoform X1 with the two alternative transcripts identified.*** *MUSCLE alignment of the genomic sequences of COP1-like isoform X1, COP1-like isoform X1 long and COP1-like isoform X1 short. The symbol * indicates perfect alignment. The red symbol represents the portion of the CDS exclusive of COP1-like isoform X1 long, which is spliced out of COP1-like isoform X1 short. The exons are highlighted in grey. The premature STOP codon of COP1-like isoform X1 short, as a result of an alternative splicing event, is in red. Highlighted in purple is the STOP codon of COP1-like isoform X1 long.*

***Figure S8.*** ***Additional tomato COP1 proteins localizations.*** *GFP-COP1 homolog and GFP-COP1-like isoform X1 fusion proteins were co-expressed with the nuclear localization signal (NLS)-mCherry protein in tomato protoplasts incubated in the dark (A, B) or under 6 hours of light exposure (C, D). In C and D, alternative localizations compared to those shown in Fig. 4B are shown. Images of protoplasts stained with GFP, RFP, and merged bright field + GFP + RFP are shown.*

**Figure S9. Representative Western Blots probed with specific antibodies for HY5 and histone H3**. Uncropped unedited membranes, as detected with the ChemiDoc system and used for Fig. 4, probed with specific antibodies for HY5 and histone H3.

***Figure S10. Complementation assay of Arabidopsis thaliana cop1-6 knockout mutants with tomato COP1 proteins.*** *(A) PCR screening for the presence of the transgene in putative SlCOP1 homolog overexpression lines. Different numbers represent different transgenic lines. The pO7WG2_COP1 homolog vector used for transformation was used as a positive control (C+). (B) PCR screening for the presence of the transgene in putative SlCOP1-like isoform X1 overexpression lines. Different numbers represent independent transgenic lines. The pO7WG2_COP1 homolog vector used for transformation was used as a positive control (C+). Expression analysis of the endogenous AtCOP1 (C), SlCOP1-like isoform X1 (D) or SlCOP1 homolog (E) in Col-0, cop1-6 and SlCOP1-like isoform X1 or SlCOP1 homolog overexpression lines. For the overexpression lines, samples were obtained by pooling one sample from each different overexpression line.*

***Figure S11. Screening of the hy5 knockout line.*** *(A, B) Results of the analysis performed with SYNTHEGO ICE software on a putative hy5 knockout line. (C) Sequence alignment of the genomic fragment containing the 200 bp flanking the cut site for the chosen guide RNA on HY5 gene sequence. The 1 nt deletion site is highlighted in red inside a red box. (D) Alignment of the wild-type HY5 control protein with the putative sequence derived from the translation of the mutated HY5 gene in the hy5 knockout line. Due to the presence of a premature stop codon in the relative CDS, the edited sequence is only 89 amino acids long, compared to the 149 amino acids wild-type protein. (E) Graphical representation of the functional domains of HY5 protein in wild type and edited sequences. In the wild-type sequence the COP1 interacting domain is represented in yellow; the Basic Motif is represented in purple, the Leucine Zipper is represented in blue. In the edited sequence the first 17 amino acids shared with the wild-type protein are represented in white, the following 72 amino acids different from the corresponding ones of the wild protein, as a result of the sequence frameshift in the edited HY5 CDS, are represented in pink.*

***Figure S12. A line harbouring the hp2 mutation in a SunBlack tomato genotype accumulates anthocyanins at high temperatures.*** *Phenotypes of Aft/atv and Aft/atv/hp2 fruits in a SunBlack tomato genotype (Mazzucato et al., 2013) at control and warm temperatures. The full original pictures, cropped to produce Fig. 7A, are shown here.*

**Supplementary Material**

***Table S1.*** *List of oligonucleotide primers used for CDS cloning.*

| **Gene Name** | **Primers_ID** | **Forward primer** | **Reverse primer** |
| --- | --- | --- | --- |
| *Solyc12g005950* | *COP1 homolog* | CACCATGGTGGAAAGTTCAGTTGG | TCAAGCTGCAAGGACTAACACTTTTATAG |
| *Solyc12g005950* | *COP1 homolog*  *(without STOP codon)* | CACCATGGTGGAAAGTTCAGTTGG | TTTAGCTGCAAGGACTAACACTTTTATAG |
| *Solyc11g011980* | *COP1-like isoform X1* | CACCATGGGAGGAGGAGAGG | TGATCAAGCAGCAAGAACAAGT |
| *Solyc11g011980* | *COP1-like isoform X1*  *(without STOP codon)* | CACCATGGGAGGAGGAGAGG | TTTAGCAGCAAGAACAAGTGCTTTTA |
| *Solyc08g061130* | *HY5 (without STOP codon)* | CACCATGCAAGAGCAAGCGACGAGTT | CTTCCTCCCTTCCTGTGCAC |
| *Solyc09g091510* | *CHS1* | CACCCCTTGTTTTCCACACGTTAAAGT | TTTCGCCGGAAAAAATGGTGGATA |
| *Solyc05g053550* | *CSH2* | CACCTGTCCAAATATGAGACTAATAT | TCGCGGTTTTTTTTTTTTTTTTACTAAG |
| *Solyc02g083860* | *F3H* | CACCCTTAAAGGATAAAGTAATTGAGAA | GTTTTGTTCAAAAGAGTTATGAGGTGC |
| *Solyc09g065100* | *AN1* | CACCGTTATATGTAATAAGGATTGTAC | CATTAATCTACTAGATTTAATTATTTGATTTTAAAC |

***Table S2.*** *List of oligonucleotide primers used for qPCR analysis.*

| **Gene Name** | **Primers_ID** | **Forward primer** | **Reverse primer** |
| --- | --- | --- | --- |
| *Solyc12g005950* | *COP1 homolog* | AGAGCATGAGAAACGGGCTTGG | TCGTGCACCAAACTTTGACCTTAC |
| *Solyc11g011980* | *COP1-like isoform X1* | TGAGCTAGCTTCTGCATCAACCG | TGTCCTTTAAACGTACGCAGTGG |
| *Solyc11g011980* | *COP1-like isoform X1 long* | TGAACCTTCTATGCTTGTATCTGG | TGGTAAGAACGCTAGCTTCCTG |
| *Solyc11g011980* | *COP1-like isoform X1 short* | AGGTCTTCGAGTTTTCATTAGTGA | CTGAAAATGTATAGTGGCTGGC |
| *Solyc08g061130* | *HY5* | GGACAACGTCAGCTTCTGGAAGAG | TGAGTCCCAGCTGATGGTTGAG |
| *Solyc03g097340* | *AN11* | ATGAAGTGGAGCCGAGAAGA | TCCATCAGCAGAAACAGA |
| *Solyc08g081140* | *JAF13* | TCAGGGGATCACTACCGAAC | TCCCATCAAGGTTGGAAGAC |
| *Solyc09g065100* | *AN1* | CTAAGAGTGCCCGCATACAGAC | ATCCGAAGTGGAGTGCTCAGATA |
| *Solyc07g052490* | *MYB-ATV* | GATTGGAATAGATCAAGCACATCA | TTCGTTGGTAGTCTCTAATGCAAC |
| *Solyc10g086250* | *AN2* | TTCCAGGAAGGACAGCAAAC | AACGAGGACGAGAATGAGGA |
| *Solyc10g086290* | *AN2-like^Aft^* | ATATTGCCAAGACATTGGGAGTG | CCATACTTGTCAATACATTTCCTCA |
| *Solyc06g005060* | *EF1a* | GCTGCTGTAACAAGATGGATGC | GGGGATTTTGTCAGGGTTGTAA |
| *Solyc11g005330* | *ACTIN2* | TTGCTGACCGTATGAGCAAG | GGACAATGGATGGACCAGAC |
| *Solyc08g080040* | *ANS* | GAACTAGCACTTGGCGTCGAA | TTGCAAGCCAGGCACCATA |
| *Solyc02g085020* | *DFR* | TCCGAAGACGACAACGGTTT | TGACAAGCCAAGAGCCGATAA |
| *AT2G32950* | *AtCOP1* | TGTGGGTCTCACAGTGAACAGC | CACGGGTCTCGTGATTTCCTTG |

***Table S3.*** *List of oligonucleotide primers used for HY5 CRISPR/Cas9-mediated silencing.*

| **Gene Name** | **Forward primer** |
| --- | --- |
| gRNA_top | GATTGATGATCTCTCACTACTTGA |
| gRNA_bottom | AAACTCAAGTAGTGAGAGATCATC |

***Table S4.*** *List of oligonucleotide primers used for genotyping HY5 knockout lines.*

| **Gene Name** | **Primers_ID** | **Forward primer** | **Reverse primer** |
| --- | --- | --- | --- |
| *pK7WG2* | *pK7WG2_CAS9* | GCCATCATTGCGATAAAGGAAAGG | GTAGCCTCAGCGGTCTCTCCAGAA |
| *Solyc08g061130* | *HY5_CRISPR* | TCATGTCCCGCTATTTCTTTTCT | CAAAATCTCAACCATACCTAACC |

***Table S5.*** *List of oligonucleotide primers used for genotyping Arabidopsis transgenic lines overexpressing COP1 homolog or COP1-like isoform X1.*

| **Transgene** | **Forward primer** | **Reverse primer** | **Amplicon** |
| --- | --- | --- | --- |
| *pO7WG2_COP1 homolog* | ACAAGTTTGTACAAAAAAGCAGGCTC | GGCAATCACTCTTGTTGTGAAGAT | 301 bp |
| *pO7WG2_COP1-like isoform X1* | TGAACCTTCTATGCTTGTATCTGGTA | TGTCCTTTAAACGTACGCAGTGG | 766 bp |

***Table S6.*** *List of oligonucleotide primers and restriction enzymes used for modification of “Grow ‘N’ Glow” vectors*

| **Target ID** | **Restriction sites** | **Forward primer** | **Reverse primer** |
| --- | --- | --- | --- |
| **Gateway cassette for pGNG2** | **SPEI + RRUI** | **ACTAGT**CCACTTTGTACAAGAAAGCTGAA | **TCGCGA**CAAGTTTGTACAAAAAAGCTGAA |
| **1^st^ Overlapping PCR on Gateway cassette for pJG4-5** |  | **GAATTCA**CAAGTTTGTACAAAAAAGCTG | TACGGAACTCCGGATGAGCATT |
| **2^nd^ Overlapping PCR on Gateway cassette for pJG4-5** |  | ATCCGGAGTTCCGTATGGCAAT | **CTCGAGA**CCACTTTGTACAAGAAA |
| **Gateway cassette for pJG4-5** | **ECORI + XHOI** | **GAATTCa**CAAGTTTGTACAAAAAAGCTG | **CTCGAGa**CCACTTTGTACAAGAAA |

***Figure S1. Anthocyanin synthesis in tomato fruit is repressed by high temperatures.*** *Phenotypes of Aft/atv and Aft/atv/hp2 fruits in the MicroTom background at control and warm temperatures. The full original images, cropped to produce Fig. 1B, are shown here.*

***Figure S2.*** ***Gene expression analysis in fruit peel under different temperatures.*** *Bar plots showing gene expression levels of biosynthetic genes (ANS, DFR), regulatory genes (AN2, AN2-like, AN11, JAF13, AN1, MYB-ATV) of the anthocyanin pathway, and photomorphogenesis-related genes (HY5, COP1 homolog, COP1-like isoform X1) in peel from fruits at the mature green stage of Aft/atv and Aft/atv/hp2 lines grown at warm (30°C) and control temperatures (23°C). For each gene, expression data were normalized by dividing each value by the global mean of all samples for the gene, followed by Log2(x+1) transformation. Data are means of 6 biological replicates ± SD. Letters indicate statistically significant differences as assessed by ANOVA and Tukey’s post-hoc test for each single gene, P ≤ 0.05.*

**Consensus sequences**

CACGTG G-BOX

ACGT ACE-BOX

>*pCHS1_ Solyc09g091510*

CCTTGTTTTCCACACGTTAAAGTCAATCTTCAAACACCACACAACATACCTACTTAACAATCAATACAAATACAGTACATTCTCCTAATGGAACCAAACCAAAGAAATGTTTAAATTTTTGTCACATTAGATGTTTTCCTATATATGTTGTGTACATTCTACTTACCTTTCATTTTACCAAACATCCGTCCGAAATTTATTGACCCGATTAATTTGAATTTAATTAAACAATAGAAAATTTACGATGAAATATTTTAGTGTGTTGATAGTTAATGATAAAATAACTATTTCACGAATTAAATGTATGATATCTAACTAAAAATAATTTATTTTTTTTACCGTTTGATCTCTATATGTCCATCAAATTTTACTTCATTCATGTACTTCCCCCATATATTAGGTAAAACTCTAACTTAAATAATATATGACCAAGCATATTTAACATTTAACCTTTAGGTTATGAACATATTCTACAAATTTTGTAATTACTTGTGTATTATAAAAATGTATAGTAGCTATAGGTTGTTAATTACGTCATATTTAAAAGTAATTAATGTAGTCTAAAAATTAGTTTGAATACAACATTTTTCGACATCAAAGAATATTCAACATATATTTTTTACAAATTGAAGTTTAAATATATTGAATCATGGAAATTAAAATTAAAATTATTTTGTCAAATTATAAATAAATCAAAAGTTCTATTATCTCCTTAAAAAACAAATAACTATCTGTTATTTGAAACTCTTTCAAATGCTCTTGAAATTTTTAAAATTTAAATTCGATATATCTAATTCTACACATAATTCATTTCATTCATGCATCTATCACTTTTGCTAATAATTTTTTTTGTGGCCACCTCATAAGACTCAAAATGTGAAAACATATGATATGTTTTTTTCTGCATTCATTGTTTTCCTAAAAAAGATGAGTTAGAATTATTATTTTGGAAAGTGCTAAATGATTTAGAAAATTTAATCAAATTTTGGCTAGAAAATTAAGAAAATAATACTATTTTTTAAAAAAGAATAAATTAATATTTTTTTTCAAAAAGTAAAATAATATAGATAAAATATTATTTTGATCAAATTTTCTGGCCAAAAAGGTTTGCCTATTATATTTTTAAGAAAGAAAAAAATCTACTTAATTTATGTGTAAATATACATATTGACTATTTATTTAATTTGGTAATGACAAACTTATGTTCAAAATTTCTGTAAAATGTGCTGTAAATTGTTAAATTCTACAGAATTAAATTCTCACCTCATAAGTCAAAATCTCATAATTAACTTACATGTCCAAAATTTCTTGTAAAATATTAAATTCCACAAAATTAAATTCATAACACATAGGTTATAATGTCATAATTAAATAAGAAATATATTTGATTTTTCAAAGAATATTACGGTTCGAAGTTGAGGTATATCAGTTTTAAAGTCTCTTTTATCATAAAATTATGCTATATATACAATAACGACACATGTAATATATATAATCCCACAAATAAATATAGATCTTGCTCCTACCTTATGAACCATTTCGAAACTCCGAACTTATAAAGATTGAGAAGTTTAATCCGTATCGAATTTACCCTAAAAAAGTTAAAATTCTTCATATAGTGTTACGTGTCAACTTCTTGTCCAAAAGCCAAATGTATATCAAAGGTGATGCAATAAAATAACAGAAATCTTATGTAAGATTTCATCAACACCGTCAATTTTTATTTCAATATAATTAACAATATTTTATGATCGTTGATCTTGATAAAGTAGGTAGCTGTCCAATATTTATTATTATTTATCTTAAATTTAGAAAGGAAAACCAAAAAAAAAAACACAAAGTCACGTGACATTAGTTGTTGAAGCACGTGATCTCTAGCTACCATTCTTTTTCTTTTGGACTTCATATATAAATATATAGTTCATACAACCCCCATGCAACAAAAATACACCAAGACAATTATCACTCTTTCATTCACGTAGTCCTAAACACAAAAAAACCTAGCATATCCACCATTTTTTCCGGCGAAA

>*pCHS2_Solyc05g053550*

TGTCCAAATATGAGACTAATATTAAATTTAATTTATTTTAGATTTATCGTATCAAAATAGACTTGCATGTATCTAGGATACATACACAAATCTCGTTCCTCTCGTTCGATTTTGCTTTCCACTTTCCTATGTATTTAATATCTCAAATGTATCTAGTTCGATTCATATGTATTTGGAATACATGATTAACTCCCTCCATATCTCCTTGACCTCTCTCCCTATTTCTTGTATATGGTAAAAAAAATAAAAATACATGTATATATTTAGATGAATCTTCCTAAATATGTGACATAAAACTCTTAATTAATGATAAATATGTAATTATTAAAAATTATAAATAGAATTAGTATATATATAATATAAATATATGTCTAATTAATGAGATAGTTTTTTCCGGTGGTTACGTCTTAAATTGGATATTACGATGTTAATTAAATTATTGTATCTAACTATATTAGAACATGTTTTATGTATAATATATGTCCAAGTTTAGCATATAAAATTTAAAGGTACTTTTTTACTTACCACATTAACTAAAAAAACTTATATTATACGTGTTATACTCTTGTTGTGACGTTGATTTTTTGTTATCATGTATAAAAATGATTTAAAAAATTAAATATAATTCATTGGATTTTTATAAACATATTGACCAAAAATAGTGTAACGATTTTATTTTGAAAAGTAGGTAGCATTAGTGGCAAATAGAGCTTTTCGATAAAAGTGCAATGAATATTCATTATATAAGTTATAATTTTTTTACTCAAAATTGTATATAATAGCAAACTAATAACCTAAAACAAATGGAGTAGCTAGAGTTTGATTTAATTGTGCTCCATAGCAAACGTTTGCAAAAAATTGTCAGGCGCCTCTCTCCCAAATATCTCGCTCGCCACTCTCCTCTAATCTCTCGCTCACTTCCTCACTTTTTATACAAACACAAGTGTATAAAAATTATTTCTAATTGTATAAAACAGAGAAAATTGTATAAGTACATATATTTTTGTTCCCCTCTTCCAGATCTCGCTCGCCACTCTCCCAAATCTCGCTCGCTACCCTCGCCTTTCTCACTTATACAAACAGAAGTGAAATGTATAAATTGTGTTTTTATTTGTATAAAGCGTGAGAAAATTGTATATACACATGCAACTACATATATTTTCGTCCTATACACTTATAATTATACAATAAAAATACTCCCCTGCCCAGTTTCTTTTGCTTTTCTCTCTTTCTCGTTTTAGACAATTCGATTCAATTGTATATTCCTTGTCAAGTCTCTTTTGTCTTTCTCTCTTTCTCATTTTATACAAATTCAAATTGTATATAATCATTCTATACACTTATAATAATACAATTCGTTTTATACACTTCGTTTTTATACAGTTCTCTGCCCAAGTGTCTTTCTCTTTCTTGTTTTATATACTTCGTTTTATACAATTTGCTTCAACTGTATATGTATAACGAATTATACAGTTTCTATGTTTGCTATGGAGCGCTATTATGCAAACTTTGCTATAGCATACAAATATGATTTTTTTATTTGCTATATGCTAAAGTTACCCTTTTTTATTATCAATTAGATTAATTTTACCCGTTATATAATAATGTGATTCGATTTATTTATGTAATATTAACAATGTATAAAAGGTAAATTTTATTTTCTCGTTATCAAATTTAAATGTATTGAATAATTTAATTGATCAATAATTTTAAATTGAAGATAAAAAATATTTATCATCCGAACAACTCCGCTCGTCGATTACGAAAAATAAGAGTTGGGGAAATATTATCTGAAAATATTATGTGACAGATGGGCCTAAAAAAGTTGCTCAAACACGTGAAGACTAGCTACCGCCTACCAGTTTGTTATGAAAATTTCTTCTATAAATATCAAAATCTCCATGCAAACTTAACACAAGCCATTTTCTCTTCTTCACTTGCTAAAATTTGTATAATATTTTTGTATTTTCATATACATAAATCTTAGTAAAAAAAAAAAAAAAACCGCGA

>*pF3H_Solyc02g083860*

CTTAAAGGATAAAGTAATTGAGAAGAAATAAATCTGAAAATTACTCTTGGAGTAATAAAATTGAAATTTACTCATATAATAGTAAATCAGAAATTTACTAAAGCAAAAGACACATGACGTAGTAAACTTGGAGTTTACGAATACTAATAATTTTATTAGTTGGCATGAATTATTTGGTCATCCCAAAGACGTGCATACTAAGTAATGGACATACATTGAAAAACTAGAAGATTCTTCAAGAATTTTTTACGTTGCTTGTTCTCGTGATAAAGTTGATTGGATAAACTAAAGTTGGGACTAAATCTCTAAATTCTGAAAAGTATAAAAGGTGAATATGGGCCCGTTCACCTATCTTGTGATATGATAAAAAGATGCATCAAAAAGATAATCACATGTGCATTTGTTGTCAACTTGCAGTTTGACATTCGCAAAATTGTTTCTGCAAGATAATTGAGCTAAAATCACAACTTTCAGAATATGAAATCAAGATAATTCATCTTGATAATATTGATAAATATATATAAGATGGTGTGACATTAAATCTCTCATATAACGAATCGTTGCTTATGAAAATAAAGATTCATATGTTGATCTGAAATATGATATTACATACAAACATAATTGTATGAATCAAACCAATAAGTTATGATCAATTTTTCTGTTAATTGGTTTATGGTCAGGGACCAAATAATTTTAATGTGCAATATATGATTAATGAATATACTATGATGCACAAAGATAGATTCTTCAAAAGATTGAGATATATGTTAGTTTGTCTAACATAAGGGGGAGACTGGAAGCAACAAAAGAGTTGTAAATACTCCAATTGAATGTCCCTTAAGGATAAAGTCTATGATAAAGAATCAAAATGATCATAATAAGAAGATAATGTGCTCTTGGAGAGCCTACGACATAACACTTTATGAAACCTCATGAGAGGGGTAGCTACCTAAAAATAATGAAGTGATGAGATCTCAATAAGTTCTGTCGTATTGTGAATCGATACAAAATGATTTATCGTTGACGATATCTTTGATACAATAACGCTCAATATTGTAAAATATAATGAGGATATACATTATACATCTATTAAAGCATGCTTGTGTAGAAATAATTATCAAGTGAAAAATGGAATGATGTATCTTGGTAAGCGTAAAGCTTATTTGACTTGCAATCTAGGCACTAGAAGATGTCATACATCTAATATTGAATGTTGTTACTTGACAAAATTGATATGAAAATATCCGATGGGTTCAAAATATAAAGCATATACAAGTTTTTGGAAAACTTGTTTATCATCCTCATAAGGATTAAATAGATTCAAAATGCATAGAGCATATACAAGTATTATTATGCAAGTTGATGACTAGAATTGAAACTCTTGGCAATTATTTGCTTAAAGAAGTTGAAGTAAGGAACTTGCTAAAATCTTTGACCCTGAAGGGAAGATTTATAAAAGCAGATAGAAGAAAACATATTTCGTCAAGGTTTTTCTACACTCATAAGCTCCCAAGAATGGTGAGATCAACATGCAAGAGGTCTGTTCAAGTAATATTATTGTTGATTTATTCACCAAGTCTCTATCAACTACAACTTTCAAGAAGATGGTGCACAAGTTTGGAAAACGAAGATTCTAGTCTCTGAATTGATGTTGTCGTTAGGGGGAGTTAATACGCGATGTACTCTTTTTTCCTCATAAGGTTTTGTCCCACTGGGTTTTCCTTGTAAGATTTTTAATGAGGCATCCGTAATGCGTATTATTAGATATGTGTACTTTTTTTCCTTCACTAGATTTTTTCCCATTGGGTTTTATCTAGTAAGGTTTTAACGAGTCACGTAATCTGACATTCAAGGGGGAGTGTTATAAACACTTTGTATTATAGTGAATGTCTAATTATGTGGAGTCCTTGTAGGATATGGTTAGGAATTCTACTTGGAGACCAAGTAAGATTTTCCCCATTTTCCTTCATTGTAAATAAGATTTGTGAAAAATCTATGAATCCTGAATATATTTCAAGAGAAATAAAAAGTCTTTTCTCTTTCTCCCTACTTTCTTCTCTTCTAAATTTATAGAATTTTATAGCAATATACATGTTTTTTGTTTTAGTTAGGCAACGCTGGTTGGTAATAGTCACGTGATTTGTCCTAACTCCTAACTAACATTCCATTTCATTCACCCTCCGTTTAATAGTTGAAAAGGTGAATTTTTCATATTAAACAATTATCGTGTGATGTAAAGAAAATATTGTTAGCAATTGGTGGTAACATTATTATAGTGGAAAAACAATTTCTTCGTTGCGATTATTTTTCTTCAAGAAAAAAAAACAAATTACTTAGTAGGAACTACTTTTCAACAAAGCAACTACGCTTTTCCCCTAAAATAGTACTAGTACTTAATTTTTTTTAACCCGTCCCAATAAAAATAACACATTTTTATATTAAGTAAACTCATAATAAACTGATATTAAAACTCATAATAAACTGATATACAACTACATAAATATTTATCACTTATTTTATTTCATAAATCTTAAAAAAATAATTCTATTTTAAATCGTGTCAAATTAAACTAACTTACACATATATCATATAAAATAAAATGGGTGTAAAATGGAACAGAATACAAGAGTACATAAGTACCTTTTTACGAAGTTAAACAATATTCATTTTGAGTGTTACACTTTACATTTTTAATGTATTTTACAAGAAATAAAAATAAGATAATCAATATCAAAATAAATATTTAACATAATTTTCAAACCAAGTATGGTGGTACCTACCCTACAATTGGTAAGCAAAATACAATTCTAGAAAGAGAACACGTGAAATGTTGTTAGTTGGCACGAAGGGTAGTAATAGCTCCACGCAAACACTCCCTTAAAAATCTCCACCCCTTATTTCACCTTACTACTTACCGTTCTCTCAACATTTAAAAAGGGCACCTCATAACTCTTTTGAACAAAAC

>*pDFR_Solyc02g085020*

TTAGTGAAAGACCAACGTGTTTGAATGGATCCAGTTCGTTTTAAAGAAAGAAGGAGAAGTGATGAAAAGTTCGTTAAATTATAATATAAAAAAATATTGACGGATTATAGATAATTGTCATCCACTTGACGGAATCAATCACCACTTCCAAGGACCTGAATGCTGTTATAGTTTTTTTATCCTTATATAAATGGAATACATTATGAAATTTTTAAATAAATAAAAAAACACATTGGTAATGTGAAGAAAATGTAGCAATATTATCGCACTACTACAACTTGTCAAATATTATTTGTCAAGTTTCTATCCAGGAGTCTTAATTAACAGCACATGGTTAATTTTTCTTCTGAAAGATTTCATCAGCTGTTGAAATTTTCTTTTCTTTTTTAAGAACTTGAAATCTTGTTAAAGTCTATGATATTCATCACTATTTGGATACTTTTATAGGTTAAAAAAAACACAAGTTACATATTTGACATAGGACGAAAATAGTTATATCCACTAATTAAATATACATAATTTATACACTAATTATGTATAGTTTGTGCATATTATATACCTGCAAGCTGTTTTGAGTGAATAGTGATGACTATATTAAAACTGCGACAAGCATTGCAAAGTACTGAAGTACATATAAACAAACATCTCTAGATCGATTCAAATTCAAATTTGTAAACAGGTAAAGAAAATGTTTAGCATGCCTGCAAAACAAAAAGGTTCGTCCATTTTATACTTTATTTTATTAATTTGATTTTCTAACTTTAACTTTATACGAGCTGAAAAAAAGCTAAAAATGGCTTTAAATCTTGTAAACTTAATGTACCAAATTGAAATTAAAAAGTTGTGTCAAAACAGAAAAGATATATCCTTTTTTAAAATAGATTAAAAATGAAAGTATGATAAACAAATTGAAACATAGAGAATAATTATTTTTAAATTTCAAAAGCATTAACGTGAAATTGATTCATTATTAACTTTAGATGTAATGAGCTTTCGAACAAAATAATAACATACTCGATATAATTCAACAAGCTAAGTATAGAGAAGATATATTGAATGCATACAAACTTTGTATTGAGAAGCTGTATCTAATAGAATCTTCGGCGTAAAAAAGTGAAATCAAATACCATACTACATAGTAATTCTTTATTATAGGGAAAAAGAATAAATATATCCCAAACTATTACCATTTTATCCTTAAAAAAGAAAAGATTTCAATCGCATGTATTCAATATTACCATTTACGATAGTTTTAAGATATATTTGTCCTTATTCCTTTTATTATATGTGCTGATAAGAAAGTGGCCAGACACTTCACACTAGCCCTTCTTCTCTCACCAACCAGTCACATTTGGTTAATCCAACCAAGTTATTGAACGGTATGTTGAGCACGTGCTTACCATCTAACATTACCACTCCTAATACCTCTACGTGCAAGCAGTATAAAAACAAAATACATAAACATAACTAAAATCTCATATCGTTGTAATTGTCATATACTAGTGGTAATTAAGTCCAGTTACACTTAAGGTTAAATTTGCTGACTCTTTTTTACCTTTCATTTCTGAAA

>*pAN2like_Solyc10g086290*

ACCAAGCTACCACAAAGTTATGAAGCATCAAATAAGAACTAACAAAATTTAAAAATTAGAATCTAAGAAAAGTTATTAAAAATTGTAGGTTACCGTTGAAAGTCAACAACGTGATTTATACATATTTAAAGAGAGACGAATAAAAATGCTGACCTTTATGACCGATAAATGCAATCAAAGAAATAAAATTTTGCATCTATTTAAGTAGTAACTATTGTAACTTATAAGTGACTTAACTTCTCTTTTTGAAATATATTTATACTATTTTTATGTTATTTAAATTATATAAATTATTTTCTTTATTTCATTTTACGTGAATTGATAAAAATACAAGATTTTATCAATCGAGTTCTTTAAATGTTTTTGAAAATATTTTAAATTATTAATTATTAAAATTTTATATTACATTTATTATTTTTAAATAATATATATTATTTCCTCAATCTCATTTTTATGTGTCATGGATTAAAAATTGAGAGTCAAGTAAACTTCTTAATTATTAAATATTCTATGTTGCTAATTATTGTAACTTACCTATAACATTCTTAAGAAATATTTAAATATATAATTATTTTTTAAAACAAGATAAATAAGTTAAATTTTAAAAATATAAAGATTTTGTAAATACTCTAAGCTAGAAATAAACATGTCAATAAAGAAATGTTTGTTTCTAGCTACTTCTATAATGATCAAATTATTCTACTTCTATAATGAAAATTATTCTAGTCTAAAAAGCAATCACGTAGACAGATTTATTTAAGACACTTTTATATAGTAGTAATAATGTAATGTAATTTATTTATTATTTATTATTATCTTTTTAATTAGAATACTTATTTTTATTTTTCAAAAAATACTTTTAATTATTTATGTATTTTTTCACTTCATTTTAAAATCATTATTTCGCCCATCAAATGCAACTGAACTTGTATTCGAAATAACTCTTCTTTTTCTTTTTTATTTTTATCTTCTTTTTTTTCAGAAAACTTTTTTTCCATTCAAATTATATTTAATGTTTAAATATGCTTCAGCATAAGAAAAAGTATATTCAAACAGTTAAACATAATTTGTTAAAATTATAATCATCGCCACTCTCCGTCTTCAACTTCAATTTTTTGAATTTTAAAAAAAATTAAAATAACATATAATAGCCCATATCTATGATCTAAATTTATGACATTATATTATTAATTATTTTGCAAAATCAGAGGAACCATTATGAAACATTACGGCACATTTGGTTGATTACAATCCATCCACGTCCCTTCTATACTTTACTATTGTTCTTAATATCTATATAGATCGATAGATTTTCACTCATTTTGTATAACAAATTTGTGGAAAGAGCAAAAGAAAAAAAAAACACCACCAAAGTACAAAAGGGTTTCTAAAAGGTCTTTTTTCTATATAGTTGTGTTTTTAGTATTATTTCGTTATCATCCCCTTGTTTTAACTTTACTTTCTTACCTCTTTTTATTCATTTTAAAATTTCACTTCACATTATTAAATCATAGTTTAATATGTAAGATTATAAAATTTTGAAGTGTTTTGAATTTAGGGGTAGAGTTATTAGCATGTATACATGTTTGATTGAATTCAATACATTTTATTAAAATAATATATGTATGTTAATAAATTTATTAATATATACAAATATTATATTATTTTTACTTAAAATCGTTATTGTAGGCCTTAAACTCAATAGTTGCT

>*pAN1_Solyc09g065100*

GTTATATGTAATAAGGATTGTACTATAAAAGGTAATCCTTGTTTAACTATAATATCCTTACCTTATCTGATTAGGATTCAAGGCAAAAGAAAACCCTATAAGAAGAGGACAATCGCAGATGAAGAAGATCACGACTTCACATAGAGAGAAAAGTGAGAAGTATTCATCGAGTAGAAGTCTTCAAGATCGATAGGTTTGCTAAGTTTAAAAAGTTCTTGTGTGAGAAAGTTTTAAACGTGTAAAACTATCTTCTTGTTTCGTTCTTGATTGTAAGTTACGAGTTATTGTACAAGGAGTGGGTTTGGCTTCTTGTAGGGTTGAGTTTCAGTGAGGGTTGTAACAAAAGGTGAGTTTGACCTTTTGGAGAGTAGAAGTAATCGATTGTAGTCAATCGAGAGAGTTAGTAGTGGTAAGGCTTTTTGATTATTGAGTTGTAATCACAAAATCTTATAGTTGAATTAATATAACGAGGTTTTTTCTTCCTTGAGTGAGGAAGGTTTTTAATTTAATAAGTGTTTGTGTCTTTACTTAAAAGCAACTTACAAGAACCTGGTTCTTGTTCTAGGGGATAGGTTTCTTCAGTGACCTTTTCGATATGGGTTTTTGTTAAGCCAAGATATATCTATTTGTATTTACTGAGTGTAATGCCTAATTGGATTGGCTCAAGGTCAAGTGTGGTACAAATATTAGTTGTGAAGGACATATTTATTAAGGTACGAGGAGTATCACGTGACACTGCTTCACATCAAAAACATTGCAGATATATATTTGAACAAGTGACACTTCGAGACACAAAGGTTTGTGCAACATAGTGGTTAGGTTCTTCATATTCATGGTATGTTCTTCTTGGTGTTGTTTTGGTACATTTTTTGTTCACTATTTTGAATACATTTGAAAAAGAAGAAGTGAACTCATAAGAAAAAGTTCTAATATATGGATCGAATCCCTTTAACTTGAAACAAGGGTTTAATCTAGCTTATTGTTATATAAATTGAAATATCTTATTAAAATCTCTGACTCCGATCTACCATTCTTCGATAGTGACACCCCTACTTAAATCATTTAGGGAAGCTTAGTCCTCTCATCTACTTAATTTCCTAACAATGATAAAATTTATAATTAAACATAGATGTTGACATATAGCCATAGATAGTAAGCAAACATAGGGTCAATAGTATGCGTGGTTAAATATCATTCTAATAATAATATAATTCCTAAATAATGGACCATATCTCTAATATAAAAAAATAAGTGATTTGTTAGAAAACATGGGTTATAATAGTTACAAAATCTATTAAATGAAGATATCAATACCCATGACAAAAAAAATAAAAAGACTAAGTCCATAACCATCTGGTAGTCACCAACCAGTCAAAAAAAAATCAACCACCTTCTATATTCTTTTCATCAATATCTCTCTCAAGAAAATTAAAAGATCTCTTCAGTCTTCTTTAAAAAATTCCAAAAAAAAAAAATACATTTATTTTTCTCTGCTAATTTTGTTGATTAGTTTTTTAGTGTAAGGTTTAAAATCAAATAATTAAATCTAGTAGATTAATG

***Figure S3. Promoter sequences of anthocyanin related genes used in the Yeast One-Hybrid (Y1H) assay.*** *Promoter sequences of biosynthetic (CHS1, CHS2, F3H, DFR) and regulatory (AN1, AN2like) genes used in the Y1H assay. Known HY5 binding motifs are highlighted in different colours: ACE elements in red, G-BOX in purple.*

***Figure S4.*** ***Phylogenetic analysis of tomato COP1 factors.*** *Phylogenetic tree analysing similarities among the Arabidopsis thaliana WD40 proteins AtCOP1, AtRUP1, and AtRUP2 and tomato WD40 proteins, including SlCOP1 homolog, SlCOP1-like, and SlCOP1-like isoform X1. In the tree, the tomato proteins encoded by the Solyc12g013840, Solyc07063120, Solyc10g011690, and Solyc08g081990 genes encode other members of the WD40 family.*

```
COP1-like isoform X1 long      MGGGEGNLVGVLVPTVNA-------GET--S-APAAGTE-----VVVVKTEEEEVDKDIL	45
COP1-like isoform X1 short     MGGGEGNLVGVLVPTVNA-------GET--S-APAAGTE-----VVVVKTEEEEVDKDIL	45
COP1_HOMOLOG                   --MVE-SSVGGVVPAVKGEVMRR-MG------DKEEGGSVTLRDEEVGTVTEWELDRELL	50
AT_COP1                        --MEE-ISTDPVVPAVKPDPRTSSVGEGANRHENDDGGS------GGSEIGAPDLDKDLL	51
                                   *   .. :**:*:        *          * .              ::*:::*

COP1-like isoform X1 long      CPICMQIIKDAFLTACGHSFCYMCIVTHLQNKSDCPCCSHYLTTNHLYPNFLLNKLLMKK	105
COP1-like isoform X1 short     CPICMQIIKDAFLTACGHSFCYMCIVTHLQNKSDCPCCSHYLTTNHLYPNFLLNKLLMKK	105
COP1_HOMOLOG                   CPICMQIIKDAFLTACGHSFCYMCIVTHLHNKSDCPCCSHYLTTSQLYPNFLLDKLLKKT	110
AT_COP1                        CPICMQIIKDAFLTACGHSFCYMCIITHLRNKSDCPCCSQHLTNNQLYPNFLLDKLLKKT	111
                               *************************:***:*********::**..:*******:*** *.

COP1-like isoform X1 long      SARQMAKSATPVEQLRQAIQQGCEVSVKELDSLLSLLMDKKRKMEQEEAETNLQILLEFL	165
COP1-like isoform X1 short     SARQMAKSATPVEQLRQAIQQGCEVSVKELDSLLSLLMDKKRKMEQEEAETNLQILLEFL	165
COP1_HOMOLOG                   SARQISKTASPVEQFRHSLEQGSEVSIKELDALLLMLSEKKRKLEQEEAERNMQILLDFL	170
AT_COP1                        SARHVSKTASPLDQFREALQRGCDVSIKEVDNLLTLLAERKRKMEQEEAERNMQILLDFL	171
                               ***:::*:*:*::*:*.::::*.:**:**:* ** :* ::***:****** *:****:**

COP1-like isoform X1 long      QCLKKQKVEELNEIQNDLQYIKEDINAVEKRRIELHRTRGRYSAKMRTLVDDSSAMTVRP	225
COP1-like isoform X1 short     QCLKKQKVEELNEIQNDLQYIKEDINAVEKRRIELHRTRGRYSAKMRTLVDDSSAMTVRP	225
COP1_HOMOLOG                   QMLRKQKVDELNEVQHDLQYIKEDLNSVERHRIDLYRARDRYSMKLRMLADDPIGKKPWS	230
AT_COP1                        HCLRKQKVDELNEVQTDLQYIKEDINAVERHRIDLYRARDRYSVKLRMLGDDPSTRNAWP	231
                               : *:****:****:* ********:*:**::**:*:*:*.*** *:* * **    .

COP1-like isoform X1 long      PLRDKGSGAIVSSPVNLQEQGRAGTSQTRNTDTRAPRNSQIVQRKDDNHGGSDSQDLNQP	285
COP1-like isoform X1 short     PLRDKGSGAIVSSPVNLQEQGRAGTSQTRNTDTRAPRNSQIVQRKDDNHGGSDSQDLNQP	285
COP1_HOMOLOG                   SSTDRNFGGLFSTSRNAPGGLPTGNLTFKKVDSKAQISSPGPQRKDTSISELNSQHMSQS	290
AT_COP1                        HEK--NQIGFNSNSLSIRGGNFVGNYQNKKVEGKAQGSSHGLPKKD-ALSGSDSQSLNQS	288
                                    .  .: *.  .      .*.   ::.: :*  .*    :**   .  :** :.*

COP1-like isoform X1 long      GKSLARKRRVHAQFNELQDCYLQKRRYWARQSQKQGERVANIKNREGYSAGLEDFQSVLS	345
COP1-like isoform X1 short     GKSLARKRRVHAQFNELQDCYLQKRRYWARQSQKQGERVANIKNREGYSAGLEDFQSVLS	345
COP1_HOMOLOG                   GLAVVRKKRVNAQFNDLQECYLQKRRQLANKSRVKEEKDADVVQREGYSEGLADFQSVLS	350
AT_COP1                        TVSMARKKRIHAQFNDLQECYLQKRRQLADQPNSKQENDKSVVRREGYSNGLADFQSVLT	348
                                 ::.**:*::****:**:*******  * : . : *.  .: .***** ** ******:

COP1-like isoform X1 long      TFTRYSRLRVVAELRHSDIFHSANIVSSIEFDRDDELFATAGVSRRIKVFEFSSVVNKPA	405
COP1-like isoform X1 short     TFTRYSRLRVVAELRHSDIFHSANIVSSIEFDRDDELFATAGVSRRIKVFEFSLVMIARS	405
COP1_HOMOLOG                   TFTRYSRLRVIAELRHGDLFHSANIVSSIEFDRDDELFATAGVSRRIKVFDFSSVVNEPA	410
AT_COP1                        TFTRYSRLRVIAEIRHGDIFHSANIVSSIEFDRDDELFATAGVSRCIKVFDFSSVVNEPA	408
                               **********:**:**.*:************************** ****:**

COP1-like isoform X1 long      DAQCPVVEMSTRSKLSCLSWNNYTKNHLASSDYEGIVTVWDVTTRQSVMEYEEHEKRAWS	465
COP1-like isoform X1 short     KFGVRSRKLAFLPLT---------------------------------------------	420
COP1_HOMOLOG                   DAHCPVVEMSTRSKLSCLSWNKYTKNHIASSDYDGIVTVWDVTTRQSVMEYEEHEKRAWS	470
AT_COP1                        DMQCPIVEMSTRSKLSCLSWNKHEKNHIASSDYEGIVTVWDVTTRQSLMEYEEHEKRAWS	468


COP1-like isoform X1 long      VDFCRTEPSMLVSGSDDCKVKVWCTKQEASVLTIDMKANICSVKYNPGSSAHIAVGSADH	525
COP1-like isoform X1 short     ------------------------------------------------------------	420
COP1_HOMOLOG                   VDFSRTEPSMLVSGSDDCKVKVWCTKQEASVLNIDMKANICCVKYNPGSSVHIAVGSADH	530
AT_COP1                        VDFSRTEPSMLVSGSDDCKVKVWCTRQEASVINIDMKANICCVKYNPGSSNYIAVGSADH	528


COP1-like isoform X1 long      HVHYYDLRNISQPLYIFSGHRKAVSYVKFLSNYELASASTDSTLRLWDVNENVPLRTFKG	585
COP1-like isoform X1 short     ------------------------------------------------------------	420
COP1_HOMOLOG                   HIHYYDLRNTSQPVHIFSGHRKAVSYVKFLSNNELASASTDSTLRLWDVKDNLPVRTLRG	590
AT_COP1                        HIHYYDLRNISQPLHVFSGHKKAVSYVKFLSNNELASASTDSTLRLWDVKDNLPVRTFRG	588


COP1-like isoform X1 long      HTNEKNFVGLTVNSEYLACGSETNEVFVYHKAISKPAARYRFVSDDQNDPDGDMGSFFIS	645
COP1-like isoform X1 short     ------------------------------------------------------------	420
COP1_HOMOLOG                   HTNEKNFVGLSVNNEFLSCGSETNEVFVYHKAISKPVTWHRFGSPDIDEADEDAGSYFIS	650
AT_COP1                        HTNEKNFVGLTVNSEYLACGSETNEVYVYHKEITRPVTSHRFGSPDMDDAEEEAGSYFIS	648


COP1-like isoform X1 long      AVCWKRDSPTIVTANSRGTIKALVLAA	672
COP1_HOMOLOG                   AVCWKSDSPTMLAANSQGTIKVLVLAA	677
AT_COP1                        AVCWKSDSPTMLTANSQGTIKVLVLAA	675
```

***Figure S5. Alignment of Arabidopsis and tomato COP1 protein sequences.*** *ClustalW alignment of the tomato proteins COP1-like isoform X1 long, COP1-like isoform X1 short, COP1 homolog and of Arabidopsis thaliana AtCOP1. The symbol * indicates perfect alignment. Red characters represent the cellular localization signal. The nuclear localization signals are highlighted in purple, the ring finger domain is in yellow, the coiled coil domain is in pale blue, and the WD40 domain is in green. The histidine (G) in the AtCOP1 sequence at position 524 (also present in the sequences of COP1-like isoform X1 long and COP1 homolog), which is essential for the interaction with HY5 (Stacey et al., 2000), is in grey.*

```
COP1-like isoform X1_short        ATGGGAGGAGGAGAGGGTAATTTAGTGGGAGTTTTAGTGCCGACTGTGAACGCCGGTGAA
COP1-like isoform X1_genomic      ATGGGAGGAGGAGAGGGTAATTTAGTGGGAGTTTTAGTGCCGACTGTGAACGCCGGTGAA
COP1-like isoform X1_long         ATGGGAGGAGGAGAGGGTAATTTAGTGGGAGTTTTAGTGCCGACTGTGAACGCCGGTGAA
                                  ************************************************************

COP1-like isoform X1_short        ACTTCAGCTCCGGCGGCTGGGACGGAGGTGGTGGTGGTGAAGACGGAGGAAGAGGAGGTG
COP1-like isoform X1_genomic      ACTTCAGCTCCGGCGGCTGGGACGGAGGTGGTGGTGGTGAAGACGGAGGAAGAGGAGGTG
COP1-like isoform X1_long         ACTTCAGCTCCGGCGGCTGGGACGGAGGTGGTGGTGGTGAAGACGGAGGAAGAGGAGGTG
                                  ************************************************************

COP1-like isoform X1_short        GATAAGGATATATTGTGTCCGATATGTATGCAAATTATCAAGGATGCGTTTCTAACGGCG
COP1-like isoform X1_genomic      GATAAGGATATATTGTGTCCGATATGTATGCAAATTATCAAGGATGCGTTTCTAACGGCG
COP1-like isoform X1_long         GATAAGGATATATTGTGTCCGATATGTATGCAAATTATCAAGGATGCGTTTCTAACGGCG
                                  ************************************************************

COP1-like isoform X1_short        TGTGGACATAGTTTTTGTTATATGTGTATTGTTACTCATTTGCAGAATAAAAGTGATTGT
COP1-like isoform X1_genomic      TGTGGACATAGTTTTTGTTATATGTGTATTGTTACTCATTTGCAGAATAAAAGTGATTGT
COP1-like isoform X1_long         TGTGGACATAGTTTTTGTTATATGTGTATTGTTACTCATTTGCAGAATAAAAGTGATTGT
                                  ************************************************************

COP1-like isoform X1_short        CCTTGTTGTTCTCATTATTTGACCACGAATCATCTTTATCCTAATTTCCTTCTTAATA--
COP1-like isoform X1_genomic      CCTTGTTGTTCTCATTATTTGACCACGAATCATCTTTATCCTAATTTCCTTCTTAATAAG
COP1-like isoform X1_long         CCTTGTTGTTCTCATTATTTGACCACGAATCATCTTTATCCTAATTTCCTTCTTAATA--
                                  **********************************************************

COP1-like isoform X1_short        ------------------------------------------------------------
COP1-like isoform X1_genomic      GTGAATTTTTTATTTTTTTTAAAGCTTTTTTCATGATTTTGAATTTTAGCTAGATCGGTG
COP1-like isoform X1_long         ------------------------------------------------------------


COP1-like isoform X1_short        ------------------------------------------------------------
COP1-like isoform X1_genomic      AAGGTTATACTACAGTGATACCCGTGCTTTAGCTGAATAATTACACCTATAGGGTGTAAT
COP1-like isoform X1_long         ------------------------------------------------------------


COP1-like isoform X1_short        ------------------------------------------------------------
COP1-like isoform X1_genomic      AATCACATTTGTGCTCATATTCATGAATTTAAATGTTTGATTTTGAATTTCACGTATATA
COP1-like isoform X1_long         ------------------------------------------------------------


COP1-like isoform X1_short        ------------------------------------------------------------
COP1-like isoform X1_genomic      TGAGTGTGATAATCATTTTTGATTGGAATGTATATGAATGAAGGTACTAAATGTGTTGTA
COP1-like isoform X1_long         ------------------------------------------------------------


COP1-like isoform X1_short        ------------------------------------------------------------
COP1-like isoform X1_genomic      TTGTGTTGATCGGTGAGCTGTGTAGTTTGTTTAGGGCAGTAGGGGAGTAGTATTTTTTGA
COP1-like isoform X1_long         ------------------------------------------------------------


COP1-like isoform X1_short        ------------------------------------------------------------
COP1-like isoform X1_genomic      ATTTGGGGGAATTTTTAAAGCAGATGAGATGAATTTTGGAATTCTGGTGCTTCTTTTGTT
COP1-like isoform X1_long         ------------------------------------------------------------


COP1-like isoform X1_short        ------------------------------------------------------------
COP1-like isoform X1_genomic      TTAGTAGAATTGCAATTGTTCTTCGTTTAGCAATGTAGATTTACATGCCTTGGAGGAATG
COP1-like isoform X1_long         ------------------------------------------------------------


COP1-like isoform X1_short        ------------------------------------------------------------
COP1-like isoform X1_genomic      TGGTTTTGCCCTGTTTTTGTTTATATTACTGAGAAATGCGCTGGATTTGTTGAAGTTTTT
COP1-like isoform X1_long         ------------------------------------------------------------


COP1-like isoform X1_short        --------------AGTTATTGATGAAGAAATCTGCTCGCCAAATGGCCAAAAGTGCTAC
COP1-like isoform X1_genomic      TTTTTCCATTGTGTAGTTATTGATGAAGAAATCTGCTCGCCAAATGGCCAAAAGTGCTAC
COP1-like isoform X1_long         --------------AGTTATTGATGAAGAAATCTGCTCGCCAAATGGCCAAAAGTGCTAC
                                                **********************************************

COP1-like isoform X1_short        ACCTGTGGAACAACTGCGCCAAGCAATACA------------------------------
COP1-like isoform X1_genomic      ACCTGTGGAACAACTGCGCCAAGCAATACAGCAGGTCAGATATGCATGAAAAGTTAAACA
COP1-like isoform X1_long         ACCTGTGGAACAACTGCGCCAAGCAATACA------------------------------
                                  ******************************

COP1-like isoform X1_short        ------------------------------------------------------------
COP1-like isoform X1_genomic      AGCTTTCTTCGTATCGTTGTACTCTTGTAGATGAACTAGTGAATGTTAATATGATGCTAT
COP1-like isoform X1_long         ------------------------------------------------------------
COP1-like isoform X1_short        ------------------------------------------------------------
COP1-like isoform X1_genomic      ATGTTGTAGGCTGATGTTGGTCTATTTCATTACAATCACTTTTTAGGTTGAGGGAAAGAT
COP1-like isoform X1_long         ------------------------------------------------------------


COP1-like isoform X1_short        ------------------------------------------------------------
COP1-like isoform X1_genomic      ATTATGAAGTCGTGAATTGTGAGAAACTAAACGAAAATCGAATATCTGAGCCTGTATGTC
COP1-like isoform X1_long         ------------------------------------------------------------


COP1-like isoform X1_short        ------------------------------------------------------------
COP1-like isoform X1_genomic      TTGAAAATGAATACAATTCACCTGCAAGTATTAGCAAAGATGTTGGTTAAAATACAAGGG
COP1-like isoform X1_long         ------------------------------------------------------------


COP1-like isoform X1_short        ------------------------------------------------------------
COP1-like isoform X1_genomic      CTGGTTCTTTAGGTAGAAAACTTGTAGGTTTTTATTCAACTTAGTTTTTGTGGAGAGCGT
COP1-like isoform X1_long         ------------------------------------------------------------


COP1-like isoform X1_short        ------------------------------------------------------------
COP1-like isoform X1_genomic      CCCTAACATTCCATTTCAGCAGCTCTTTTAGTTCTATAATCTCCGATGATTTTGCCTTTT
COP1-like isoform X1_long         ------------------------------------------------------------


COP1-like isoform X1_short        ------------------------------------------------------------
COP1-like isoform X1_genomic      AACACTCTCTGTCATACTCTCCGGGATCATTTCCATATAGCTGTTCAGGATTCTCATTCT
COP1-like isoform X1_long         ------------------------------------------------------------


COP1-like isoform X1_short        ---------GCAGGGATGCGAGGTGTCTGTTAAAGAGCTGGATAGTCTATTATCGCTTCT
COP1-like isoform X1_genomic      GATTCTTAGGCAGGGATGCGAGGTGTCTGTTAAAGAGCTGGATAGTCTATTATCGCTTCT
COP1-like isoform X1_long         ---------GCAGGGATGCGAGGTGTCTGTTAAAGAGCTGGATAGTCTATTATCGCTTCT
                                           ***************************************************

COP1-like isoform X1_short        TATGGACAAGAAAAGAAAAATGGAACAAGAAGAAGCTGAAACAAATCTTCAGATTCTGCT
COP1-like isoform X1_genomic      TATGGACAAGAAAAGAAAAATGGAACAAGAAGAAGCTGAAACAAATCTTCAGATTCTGCT
COP1-like isoform X1_long         TATGGACAAGAAAAGAAAAATGGAACAAGAAGAAGCTGAAACAAATCTTCAGATTCTGCT
                                  ************************************************************

COP1-like isoform X1_short        CGAGTTTCTCCAGTGTTTAAAGAAGCAAAAAGTTGAAGAGCTTAATG-------------
COP1-like isoform X1_genomic      CGAGTTTCTCCAGTGTTTAAAGAAGCAAAAAGTTGAAGAGCTTAATGAGGTTTTTTCTTG
COP1-like isoform X1_long         CGAGTTTCTCCAGTGTTTAAAGAAGCAAAAAGTTGAAGAGCTTAATG-------------
                                  ***********************************************

COP1-like isoform X1_short        ------------------------------------------------------------
COP1-like isoform X1_genomic      AACTTCTACCGACTTTACACATTGCCATTTTTGGTTTGAATTCATCTGTTGTCATTTGGA
COP1-like isoform X1_long         ------------------------------------------------------------


COP1-like isoform X1_short        ------------------------------------------------------------
COP1-like isoform X1_genomic      TACTTATTGTACTCTATTCTACTTAATTCACATCCCTCCGACAGGTAGAATTTGCTTTCC
COP1-like isoform X1_long         ------------------------------------------------------------


COP1-like isoform X1_short        ------------------------------------------------------------
COP1-like isoform X1_genomic      TTGTACTATATCTTTGAAGTCTAACTAGAGAAAGTGTGTTCAATAGAATATTATAACAAG
COP1-like isoform X1_long         ------------------------------------------------------------


COP1-like isoform X1_short        ------------------------------------------------------------
COP1-like isoform X1_genomic      GAAAGGAACATGATTTCGTTAGTAGATACCTGGAGCTTGCCTTTTCCCTTGCAAAGAAAG
COP1-like isoform X1_long         ------------------------------------------------------------


COP1-like isoform X1_short        ------------------------------------------------------------
COP1-like isoform X1_genomic      GGAGTTTGCATAATTACATATTTGTCTATCATCTAAAAAGATTAAACTCATTGAAGTATC
COP1-like isoform X1_long         ------------------------------------------------------------


COP1-like isoform X1_short        ------------------------------------------------------------
COP1-like isoform X1_genomic      TTCCTTGTGGACGTATTTCTTTTCATTTTTTATCCATTCAAGAGAATGCTGTCATCTTTT
COP1-like isoform X1_long         ------------------------------------------------------------


COP1-like isoform X1_short        ------------------------------------------------------------
COP1-like isoform X1_genomic      ATGTTTAATTTCTTCAAACTATAGGCTCCTTAGATGCTGGCAATTTTATAAACCTGTGGT
COP1-like isoform X1_long         ------------------------------------------------------------
COP1-like isoform X1_short        --------------------------------------------AGATCCAAAATGATCT
COP1-like isoform X1_genomic      ATAAAATAGACTATCCTAATCTCACCTCTCTCTGCACTTCTTCTAGATCCAAAATGATCT
COP1-like isoform X1_long         --------------------------------------------AGATCCAAAATGATCT
                                                                              ****************

COP1-like isoform X1_short        CCAGTACATTAAAGAAGACATAAATGCTGTGGAGAAGCGTAGAATAGAGTTGCATAGGAC
COP1-like isoform X1_genomic      CCAGTACATTAAAGAAGACATAAATGCTGTGGAGAAGCGTAGAATAGAGTTGCATAGGAC
COP1-like isoform X1_long         CCAGTACATTAAAGAAGACATAAATGCTGTGGAGAAGCGTAGAATAGAGTTGCATAGGAC
                                  ************************************************************

COP1-like isoform X1_short        AAGAGGTAGATATTCTGCGAAAATGAGAACGCTAGTAGATGATTCATCTGCAATGACAGT
COP1-like isoform X1_genomic      AAGAGGTAGATATTCTGCGAAAATGAGAACGCTAGTAGATGATTCATCTGCAATGACAGT
COP1-like isoform X1_long         AAGAGGTAGATATTCTGCGAAAATGAGAACGCTAGTAGATGATTCATCTGCAATGACAGT
                                  ************************************************************

COP1-like isoform X1_short        CAGGCCTCCATTGAGAGATAAGGGAAGTGGTGCAATTGTTTCTAGTCCTGTCAATTTACA
COP1-like isoform X1_genomic      CAGGCCTCCATTGAGAGATAAGGGAAGTGGTGCAATTGTTTCTAGTCCTGTCAATTTACA
COP1-like isoform X1_long         CAGGCCTCCATTGAGAGATAAGGGAAGTGGTGCAATTGTTTCTAGTCCTGTCAATTTACA
                                  ************************************************************

COP1-like isoform X1_short        AGAACAAGGTCGTGCTGGCACTTCACAGACCAGGAATACTGATACAAGAGCTCCTCGGAA
COP1-like isoform X1_genomic      AGAACAAGGTCGTGCTGGCACTTCACAGACCAGGAATACTGATACAAGAGCTCCTCGGAA
COP1-like isoform X1_long         AGAACAAGGTCGTGCTGGCACTTCACAGACCAGGAATACTGATACAAGAGCTCCTCGGAA
                                  ************************************************************

COP1-like isoform X1_short        TTCTCAAATTGTGCAACGGAAGGATGACAACCACGGTGGATCAGATTCACAGGATCTCAA
COP1-like isoform X1_genomic      TTCTCAAATTGTGCAACGGAAGGATGACAACCACGGTGGATCAGATTCACAGGATCTCAA
COP1-like isoform X1_long         TTCTCAAATTGTGCAACGGAAGGATGACAACCACGGTGGATCAGATTCACAGGATCTCAA
                                  ************************************************************

COP1-like isoform X1_short        TCAACCTGGAAAGTCATTAGCAAGGAAACGGCGCGTTCATGC------------------
COP1-like isoform X1_genomic      TCAACCTGGAAAGTCATTAGCAAGGAAACGGCGCGTTCATGCACAGGTTAATTCATTAAC
COP1-like isoform X1_long         TCAACCTGGAAAGTCATTAGCAAGGAAACGGCGCGTTCATGC------------------
                                  ******************************************

COP1-like isoform X1_short        ------------------------------------------------------------
COP1-like isoform X1_genomic      TTATTGTTGTGCTTGTTGATGGTCGTGTTAATGTTCGTATGCTCTCTCTTAACATGTTCA
COP1-like isoform X1_long         ------------------------------------------------------------


COP1-like isoform X1_short        -------ACAGTTCAATGAGCTTCAAGACTGTTACCTACAAAAGAGGCGCTATTGGGCAA
COP1-like isoform X1_genomic      ATTGTCAACAGTTCAATGAGCTTCAAGACTGTTACCTACAAAAGAGGCGCTATTGGGCAA
COP1-like isoform X1_long         -------ACAGTTCAATGAGCTTCAAGACTGTTACCTACAAAAGAGGCGCTATTGGGCAA
                                         *****************************************************

COP1-like isoform X1_short        GACAGTCGCAGAAACAGGGAGAAAGGGTAGCAAATATCAAGAATAGAGAAGGTTATTCTG
COP1-like isoform X1_genomic      GACAGTCGCAGAAACAGGGAGAAAGGGTAGCAAATATCAAGAATAGAGAAGGTTATTCTG
COP1-like isoform X1_long         GACAGTCGCAGAAACAGGGAGAAAGGGTAGCAAATATCAAGAATAGAGAAGGTTATTCTG
                                  ************************************************************

COP1-like isoform X1_short        CGGGACTTGAGGATTTTCAATCTGTTTTGTCTACCTTTACGCGATAC-------------
COP1-like isoform X1_genomic      CGGGACTCGAGGATTTTCAATCTGTTTTGTCTACCTTTACGCGATACAGGTACATCAGTT
COP1-like isoform X1_long         CGGGACTCGAGGATTTTCAATCTGTTTTGTCTACCTTTACGCGATAC-------------
                                  ******* ***************************************

COP1-like isoform X1_short        ------------------------------------------------------------
COP1-like isoform X1_genomic      CATCTTATAATGGTAGTATTGGCTGCAAATTATCCTAAATATTTGGGCTTAACTTTCTTT
COP1-like isoform X1_long         ------------------------------------------------------------


COP1-like isoform X1_short        -------------------AGTCGTCTGCGTGTTGTTGCTGAACTCAGGCATTCTGACAT
COP1-like isoform X1_genomic      CGACTGTGCTTGTTGAAATAGTCGTCTGCGTGTTGTTGCTGAACTCAGGCATTCTGACAT
COP1-like isoform X1_long         -------------------AGTCGTCTGCGTGTTGTTGCTGAACTCAGGCATTCTGACAT
                                                     *****************************************

COP1-like isoform X1_short        TTTTCACTCAGCCAATATTGTTTCG-----------------------------------
COP1-like isoform X1_genomic      TTTTCACTCAGCCAATATTGTTTCGAGGTAAGAAGTTCATTCTCAGTACTAGGGATAATC
COP1-like isoform X1_long         TTTTCACTCAGCCAATATTGTTTCG-----------------------------------
                                  *************************

COP1-like isoform X1_short        ------------------------------------------------------------
COP1-like isoform X1_genomic      GGTTATGAGAAACAGGACAGGTTCTACTCTTATTAGTAGTTTTTTCCTGGATTCATTTTA
COP1-like isoform X1_long         ------------------------------------------------------------


COP1-like isoform X1_short        -----------------------------------------------------AGTATTG
COP1-like isoform X1_genomic      GTCAAAACCTTTTTTGTGTGTTCTGCAAAGCAACTAAATTTCCTCTTCTTGACAGTATTG
COP1-like isoform X1_long         -----------------------------------------------------AGTATTG
                                                                                       *******

COP1-like isoform X1_short        AATTTGACCGAGATGATGAATTATTTGCTACTGCTGGGGTATCACGAAGAATTAAGGTCT
COP1-like isoform X1_genomic      AATTTGACCGAGATGATGAATTATTTGCTACTGCTGGGGTATCACGAAGAATTAAGGTCT
COP1-like isoform X1_long         AATTTGACCGAGATGATGAATTATTTGCTACTGCTGGGGTATCACGAAGAATTAAGGTCT
                                  ************************************************************

COP1-like isoform X1_short        TCGAGTTTTCAT------------------------------------------------
COP1-like isoform X1_genomic      TCGAGTTTTCATCAGTAAGTTCATGCTTCTTGTGAGCTTCTTAATTTCTTCTAAGCTTCC
COP1-like isoform X1_long         TCGAGTTTTCATCA----------------------------------------------
                                  ************

COP1-like isoform X1_short        ------------------------------------------------------------
COP1-like isoform X1_genomic      CACATGAAAAATTTCTGCTAGTAGTTGAATAATTGTTCTATTAATTAAACTTGATTTCTA
COP1-like isoform X1_long         ------------------------------------------------------------


COP1-like isoform X1_short        ------------------------------------------------------------
COP1-like isoform X1_genomic      GCTCCCTTTTTCTTCTCATATAGGATTTTGAGATTTTTGCATGCTAGATGTTTTCGGATA
COP1-like isoform X1_long         ------------------------------------------------------------


COP1-like isoform X1_short        ------------------------------------------------------------
COP1-like isoform X1_genomic      AATTTAACTGTTTGTAGTTGAATTTTTCAATAATTTGCTAGATGTGAGATTCTTATGAGT
COP1-like isoform X1_long         ------------------------------------------------------------


COP1-like isoform X1_short        ------------------------------------------------------------
COP1-like isoform X1_genomic      TCTGCTTTTCTTTTGGCTGTAATGTTTTTGTTATTTCAAAAGATACAATTTGCTAATCAT
COP1-like isoform X1_long         ------------------------------------------------------------


COP1-like isoform X1_short        ------------------------------------------------------------
COP1-like isoform X1_genomic      TGTATGTTCTCTTTATCAGGTGGTAAACAAACCAGCTGATGCGCAATGTCCTGTTGTTGA
COP1-like isoform X1_long         -------------------GTGGTAAACAAACCAGCTGATGCGCAATGTCCTGTTGTTGA


COP1-like isoform X1_short        ------------------------------------------------------------
COP1-like isoform X1_genomic      AATGTCTACCCGATCTAAACTAAGTTGTTTGAGTTGGAACAACTACACTAAAAACCACTT
COP1-like isoform X1_long         AATGTCTACCCGATCTAAACTAAGTTGTTTGAGTTGGAACAACTACACTAAAAACCACTT


COP1-like isoform X1_short        ------------------------------------------------------------
COP1-like isoform X1_genomic      AGCAAGCAGTGACTATGAGGGCATAGTAACGGTTTGGGATGTTACAACTCGTCAGGTAGT
COP1-like isoform X1_long         AGCAAGCAGTGACTATGAGGGCATAGTAACGGTTTGGGATGTTACAACTCG---------


COP1-like isoform X1_short        ------------------------------------------------------------
COP1-like isoform X1_genomic      ATCATCTTATATCTAGATAATGAAAAACACCTTCATATGAATCTGCAGTTTATATCTGAT
COP1-like isoform X1_long         ------------------------------------------------------------


COP1-like isoform X1_short        ------------------------------------------------------------
COP1-like isoform X1_genomic      GGGATAAGATTTAACACTCTCTTTTTCAGAGTGTGATGGAGTACGAGGAGCATGAAAAAC
COP1-like isoform X1_long         -------------------------TCAGAGTGTGATGGAGTACGAGGAGCATGAAAAAC


COP1-like isoform X1_short        ----------------------------------------------------TAGTGATG
COP1-like isoform X1_genomic      GAGCATGGAGTGTTGACTTTTGTCGTACTGAACCTTCTATGCTTGTATCTGGTAGTGATG
COP1-like isoform X1_long         GAGCATGGAGTGTTGACTTTTGTCGTACTGAACCTTCTATGCTTGTATCTGGTAGTGATG
                                                                                      ********

COP1-like isoform X1_short        ATTGCA------------------------------------------------------
COP1-like isoform X1_genomic      ATTGCAAGGTAAAAAGAAATTCGGAACATGTGTTTTCTCAAGTTTACGTGCTTCCTAATT
COP1-like isoform X1_long         ATTGCA------------------------------------------------------
                                  ******

COP1-like isoform X1_short        ------------------------------------------------------------
COP1-like isoform X1_genomic      TGGTTTTCGGAAAGAAAATCATAGTGTTTCTGAATGTAAAAAAAATTTGACAAATGAGGT
COP1-like isoform X1_long         ------------------------------------------------------------


COP1-like isoform X1_short        ------------------------------------------------------------
COP1-like isoform X1_genomic      AAGATAAAATTGGGATTTATCTAATATCTTTCTGCTTGGAGATCTGTAGTCAGTGTACTA
COP1-like isoform X1_long         ------------------------------------------------------------
COP1-like isoform X1_short        ------------------------------------------------------------
COP1-like isoform X1_genomic      AGTCGAAAGAACAGAACTTGTTTTGGCGAGGCGCTATACTTGGAATGTGCTAATGAATTT
COP1-like isoform X1_long         ------------------------------------------------------------


COP1-like isoform X1_short        ------AGGTCAAAGTTTGGTGTACGAAGCAGGAAGCTAGCGTTCTTACCATTGACATGA
COP1-like isoform X1_genomic      CTCAACAGGTCAAAGTTTGGTGTACGAAGCAGGAAGCTAGCGTTCTTACCATTGACATGA
COP1-like isoform X1_long         ------AGGTCAAAGTTTGGTGTACGAAGCAGGAAGCTAGCGTTCTTACCATTGACATGA
                                        ******************************************************

COP1-like isoform X1_short        AAGCTAACATTTGCTCGGTGAAGTATAATCCTGGATCCAGTGCTCACATAGCT-------
COP1-like isoform X1_genomic      AAGCTAACATTTGCTCGGTGAAGTATAATCCTGGATCCAGTGCTCACATAGCTGTAAGTT
COP1-like isoform X1_long         AAGCTAACATTTGCTCGGTGAAGTATAATCCTGGATCCAGTGCTCACATAGCT-------
                                  *****************************************************

COP1-like isoform X1_short        ------------------------------------------------------------
COP1-like isoform X1_genomic      CACATTGAAGGTTCATGAGTATTTCATGTTAAACGATATATCTTCAAACTAAGGCGTGAA
COP1-like isoform X1_long         ------------------------------------------------------------


COP1-like isoform X1_short        --------------------------GTGGGTTCTGCAGATCATCACGTTCACTATTACG
COP1-like isoform X1_genomic      CCTATATATATATATATATATTGCAGGTGGGTTCTGCAGATCATCACGTTCACTATTACG
COP1-like isoform X1_long         --------------------------GTGGGTTCTGCAGATCATCACGTTCACTATTACG
                                                            **********************************

COP1-like isoform X1_short        ACTTGAGAAATATTAGCCAGCCACTATACATTTTCAGTGGACACCGTAAAGCTGTTTCAT
COP1-like isoform X1_genomic      ACTTGAGAAATATTAGCCAGCCACTATACATTTTCAGTGGACACCGTAAAGCTGTTTCAT
COP1-like isoform X1_long         ACTTGAGAAATATTAGCCAGCCACTATACATTTTCAGTGGACACCGTAAAGCTGTTTCAT
                                  ************************************************************

COP1-like isoform X1_short        ATGTAAAATTTCTATCAAACTATGAGCTAGCTTCTGCATCAACCGATAGTACACTGCGTT
COP1-like isoform X1_genomic      ATGTAAAATTTCTATCAAACTATGAGCTAGCTTCTGCATCAACCGATAGTACACTGCGTT
COP1-like isoform X1_long         ATGTAAAATTTCTATCAAACTATGAGCTAGCTTCTGCATCAACCGATAGTACACTGCGTT
                                  ************************************************************

COP1-like isoform X1_short        TGTGGGACGTCAATGAGAATGTTCCA----------------------------------
COP1-like isoform X1_genomic      TGTGGGACGTCAATGAGAATGTTCCAGTAAGTAATCATGCTCCCATCATTTTCTCAATCA
COP1-like isoform X1_long         TGTGGGACGTCAATGAGAATGTTCCA----------------------------------
                                  **************************

COP1-like isoform X1_short        ------------------------------------------------------------
COP1-like isoform X1_genomic      GAAAGACAATAATCTAATTTCTAAGAGCCAGTAATATGTGAAAAACCAATTCATACTGAA
COP1-like isoform X1_long         ------------------------------------------------------------


COP1-like isoform X1_short        ---------------------------------CTGCGTACGTTTAAAGGACATACAAAC
COP1-like isoform X1_genomic      CCAGCATTTTCCTTGTTCTGTTTCTACTCTCAGCTGCGTACGTTTAAAGGACATACAAAC
COP1-like isoform X1_long         ---------------------------------CTGCGTACGTTTAAAGGACATACAAAC
                                                                   ***************************

COP1-like isoform X1_short        GAGAAGAACTTTGTCGGTCTTACAGTAAACAGCGAATACCTTGCTTGTGGAAGTGAGACA
COP1-like isoform X1_genomic      GAGAAGAACTTTGTCGGTCTTACAGTAAACAGCGAATACCTTGCTTGTGGAAGTGAGACA
COP1-like isoform X1_long         GAGAAGAACTTTGTCGGTCTTACAGTAAACAGCGAATACCTTGCTTGTGGAAGTGAGACA
                                  ************************************************************

COP1-like isoform X1_short        AATGAAGTCTTTGTCTACCACA--------------------------------------
COP1-like isoform X1_genomic      AATGAAGTCTTTGTCTACCACAAGGTTAAGAAACTATTTGAGAAAATACGATTTAACATC
COP1-like isoform X1_long         AATGAAGTCTTTGTCTACCACA--------------------------------------
                                  **********************

COP1-like isoform X1_short        --------------------------------------AGGCGATTTCTAAACCAGCAGC
COP1-like isoform X1_genomic      GTTTTCCAGTGCTTAATCTGTTATTGAACTGTATGTGCAGGCGATTTCTAAACCAGCAGC
COP1-like isoform X1_long         --------------------------------------AGGCGATTTCTAAACCAGCAGC
                                                                        **********************

COP1-like isoform X1_short        TCGGTACAGATTTGTTTCTGATGATCAGAATGATCCAGACGGGGACATGGGATCATTTTT
COP1-like isoform X1_genomic      TCGGTACAGATTTGTTTCTGATGATCAGAATGATCCAGACGGGGACATGGGATCATTTTT
COP1-like isoform X1_long         TCGGTACAGATTTGTTTCTGATGATCAGAATGATCCAGACGGGGACATGGGATCATTTTT
                                  ************************************************************

COP1-like isoform X1_short        CATCAGTGCTGTTTGTTGGAAACGTGACAGCCCAACCATTGTTACAGCAAACAGTCGAGG
COP1-like isoform X1_genomic      CATCAGTGCTGTTTGTTGGAAACGTGACAGCCCAACCATTGTTACAGCAAACAGTCGAGG
COP1-like isoform X1_long         CATCAGTGCTGTTTGTTGGAAACGTGACAGCCCAACCATTGTTACAGCAAACAGTCGAGG
                                  ************************************************************


COP1-like isoform X1_short        GACAATAAAAGCACTTGTTCTTGCTGCTTGA
COP1-like isoform X1_genomic      GACAATAAAAGCACTTGTTCTTGCTGCTTGA
COP1-like isoform X1_long         GACAATAAAAGCACTTGTTCTTGCTGCTTGA
                                  *******************************
```

***Figure S6. Alignment of the gene sequence of COP1-like isoform X1 with the two alternative transcripts identified.*** *MUSCLE alignment of the genomic sequences of COP1-like isoform X1, COP1-like isoform X1 long and COP1-like isoform X1 short. The symbol * indicates perfect alignment. The red symbol represents the portion of the CDS exclusive of COP1-like isoform X1 long, which is spliced out of COP1-like isoform X1 short. The exons are highlighted in grey. The premature STOP codon of COP1-like isoform X1 short, as a result of an alternative splicing event, is in red. Highlighted in purple is the STOP codon of COP1-like isoform X1 long.*

***Figure S7. Additional negative controls for the split-luciferase assay in tomato protoplasts****. Data are expressed as relative luciferase activity (RLU1) with the Renilla level normalized on the total content of proteins and with the value of the negative control set to 1, and are means of four biological replicates ± SD.*

***Figure S8.*** ***Additional tomato COP1 proteins localizations.*** *GFP-COP1 homolog and GFP-COP1-like isoform X1 fusion proteins were co-expressed with the nuclear localization signal (NLS)-mCherry protein in tomato protoplasts incubated in the dark (A, B) or under 6 hours of light exposure (C, D) at 23°C. In C and D, alternative localizations compared to those shown in Fig. 4B are shown. Images of protoplasts stained with GFP, RFP, and merged bright field + GFP + RFP are shown.*

**Figure S9. Representative Western Blots probed with specific antibodies for HY5 and histone H3**. Uncropped unedited membranes, as detected with the ChemiDoc system and used for Fig. 4, probed with specific antibodies for HY5 and histone H3.

***Figure S10. Complementation assay of Arabidopsis thaliana cop1-6 knockout mutants with tomato COP1 proteins.*** *(A) PCR screening for the presence of the transgene in putative SlCOP1 homolog overexpression lines. Different numbers represent different transgenic lines. The pO7WG2_COP1 homolog vector used for transformation was used as a positive control (C+). (B) PCR screening for the presence of the transgene in putative SlCOP1-like isoform X1 overexpression lines. Different numbers represent independent transgenic lines. The pO7WG2_COP1 homolog vector used for transformation was used as a positive control (C+). Expression analysis of the endogenous AtCOP1 (C), SlCOP1-like isoform X1 (D) or SlCOP1 homolog (E) in Col-0, cop1-6 and SlCOP1-like isoform X1 or SlCOP1 homolog overexpression lines. For the overexpression lines, samples were obtained by pooling one sample from each different overexpression line.*

***Figure S11.*** ***Screening of the hy5 knockout line.*** *(A, B) Results of the analysis performed with SYNTHEGO ICE software on a putative hy5 knockout line. (C) Sequence alignment of the genomic fragment containing the 200 bp flanking the cut site for the chosen guide RNA on HY5 gene sequence. The 1 nt deletion site is highlighted in red inside a red box. (D) Alignment of the wild-type HY5 control protein with the putative sequence derived from the translation of the mutated HY5 gene in the hy5 knockout line. Due to the presence of a premature stop codon in the relative CDS, the edited sequence is only 89 amino acids long, compared to the 149 amino acids wild-type protein. (E) Graphical representation of the functional domains of HY5 protein in wild type and edited sequences. In the wild-type sequence the COP1 interacting domain is represented in yellow; the Basic Motif is represented in purple, the Leucine Zipper is represented in blue. In the edited sequence the first 17 amino acids shared with the wild-type protein are represented in white, the following 72 amino acids different from the corresponding ones of the wild protein, as a result of the sequence frameshift in the edited HY5 CDS, are represented in pink.*

***Figure S12.*** ***A line harbouring the hp2 mutation in a SunBlack tomato genotype accumulates anthocyanins at high temperatures.*** *Phenotypes of Aft/atv and Aft/atv/hp2 fruits in a SunBlack tomato genotype (Mazzucato et al., 2013) at control and warm temperatures. The full original pictures, cropped to produce Fig. 7A, are shown here.*
